# Isolation and Structure Determination of Echinochrome A Oxidative Degradation Products

**DOI:** 10.3390/molecules25204778

**Published:** 2020-10-18

**Authors:** Natalia P. Mishchenko, Elena A. Vasileva, Andrey V. Gerasimenko, Valeriya P. Grigorchuk, Pavel S. Dmitrenok, Sergey A. Fedoreyev

**Affiliations:** 1 G.B. Elyakov Pacific Institute of Bioorganic Chemistry, Far Eastern Branch of the Russian Academy of Sciences, Vladivostok 690022, Russia; vasilieva_el_an@mail.ru (E.A.V.); paveldmitrenok@mail.ru (P.S.D.); fedoreev-s@mail.ru (S.A.F.); 2 Institute of Chemistry, Far Eastern Branch of the Russian Academy of Sciences, Vladivostok 690022, Russia; gerasimenko@ich.dvo.ru; 3 Federal Scientific Center of the East Asia Terrestrial Biodiversity, Far-Eastern Branch of Russian Academy of Sciences, Vladivostok 690022, Russia; kera1313@mail.ru

**Keywords:** histochrome, echinochrome A, oxidative degradation, HPLC–DAD–MS, NMR

## Abstract

Echinochrome A (Ech A, **1**) is one of the main pigments of several sea urchin species and is registered in the Russian pharmacopeia as an active drug substance (Histochrome^®^), used in the fields of cardiology and ophthalmology. In this study, Ech A degradation products formed during oxidation by O_2_ in air-equilibrated aqueous solutions were identified, isolated, and structurally characterized. An HPLC method coupled with diode-array detection (DAD) and mass spectrometry (MS) was developed and validated to monitor the Ech A degradation process and identify the appearing compounds. Five primary oxidation products were detected and their structures were proposed on the basis of high-resolution electrospray ionization mass spectrometry (HR-ESI-MS) as 7-ethyl-2,2,3,3,5,7,8-heptahydroxy-2,3-dihydro-1,4-naphthoquinone (**2**), 6-ethyl-5,7,8-trihydroxy-1,2,3,4-tetrahydronaphthalene-1,2,3,4-tetraone (**3**), 2,3-epoxy-7-ethyl-2,3-dihydro-2,3,5,6,8-pentahydroxy-1,4-naphthoquinone (**4**), 2,3,4,5,7-pentahydroxy-6-ethylinden-1-one (**5**), and 2,2,4,5,7-pentahydroxy-6-ethylindane-1,3-dione (**6**). Three novel oxidation products were isolated, and NMR and HR-ESI-MS methods were used to establish their structures as 4-ethyl-3,5,6-trihydroxy-2-oxalobenzoic acid (**7**), 4-ethyl-2-formyl-3,5,6-trihydroxybenzoic acid (**8**), and 4-ethyl-2,3,5-trihydroxybenzoic acid (**9**). The known compound 3-ethyl-2,5-dihydroxy-1,4-benzoquinone (**10**) was isolated along with products **7**–**9**. Compound **7** turned out to be unstable; its anhydro derivative **11** was obtained in two crystal forms, the structure of which was elucidated using X-ray crystallography as 7-ethyl-5,6-dihydroxy-2,3-dioxo-2,3-dihydrobenzofuran-4-carboxylic acid and named echinolactone. The chemical mechanism of Ech A oxidative degradation is proposed. The in silico toxicity of Ech A and its degradation products **2** and **7**–**10** were predicted using the ProTox-II webserver. The predicted median lethal dose (LD_50_) value for product **2** was 221 mg/kg, and, for products **7**–**10**, it appeared to be much lower (≥2000 mg/kg). For Ech A, the predicted toxicity and mutagenicity differed from our experimental data.

## 1. Introduction

Histochrome^®^ is a solution of the sodium salt of naturally occurring quinone echinochrome A (7-ethyl-2,3,5,6,8-pentahydroxy-1,4-naphthoquinone) for intravenous injections and infusions, manufactured in ampoules (Supplementary Materials, Figure S1). Histochrome is registered in Russia as an antioxidant drug. It is used in cardiology for the treatment of coronary heart disease and for restriction of the necrosis zone in myocardial infarction (state registration number P N002363/01), and in ophthalmology for the treatment of dystrophic diseases of the retina and cornea, macular degeneration, primary open-angle glaucoma, diabetic retinopathy, hemorrhages to vitreous humor, retina, and anterior chamber, and discirculatory disorders in the central artery and retinal vein (state registration number P N002363/02). Histochrome has no known analogues, and it simultaneously blocks a number of free-radical reactions, neutralizes reactive oxygen species (ROS), nitric oxide, and peroxide radicals, chelates metal ions, inhibits lipid peroxidation, and regulates antioxidant enzyme levels [1].

**Figure 1:**
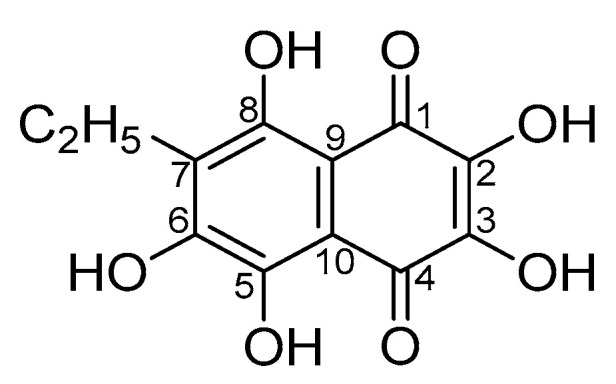
Chemical structure of echinochrome A (7-ethyl-2,3,5,6,8-pentahydroxy-1,4-naphthoquinone, Ech A).

Echinochrome A (Ech A, **1**) is one of the main pigments of various sea urchin species [2,3,4] (Figure 1). Ech A isolated from the sand dollar *Scaphechinus mirabilis* (purity >98%) is registered in the Russian pharmacopeia as an active drug substance under the international non-patented name pentahydroxyethylnaphthoquinone (state registration number P N002362/01).

Currently, more and more papers focused on elucidating the mechanisms underlying the diverse biological effects of Ech A are being published. Ech A was found to protect rat cardiomyoblasts and isolated cardiomyocytes from the effects of cardiotoxic compounds doxorubicin, *tert*-butyl hydroperoxide, and sodium nitroprusside, which cause an increase in ROS formation and depolarization of mitochondrial membranes [5]. In rat cardiomyoblast cells, Ech A dose-dependently increased the mass of mitochondria and the content of mitochondrial DNA and activated mitochondrial biogenesis, increasing the expression of the main regulators of the metabolic function of mitochondria [6]. Ech A was also found to activate mitochondrial biogenesis in skeletal muscle, increasing the endurance of rats during physical activity by increasing the number of mitochondria [7]. Being an inhibitor of the sarcoplasmic/endoplasmic reticulum Ca^2+^ ATPase 2a (SERCA2A) receptor, which is responsible for pumping calcium ions from the cytosol into the sarcoplasmic reticulum, Ech A prevented ischemic damage of the myocardium, reducing the area of myocardial infarction [8]. By reducing the level of intracellular ROS and regulating the expression of pro- and antiapoptotic proteins, Ech A protected human cardiac progenitor cells against oxidative stress [9]. This may be the basis for a simple and effective strategy to enhance myocardial regeneration by increasing the survival of transplanted cardiac cells under oxidative stress induced by ischemic damage. Ech A was found to be an effective agent for promoting cell proliferation and maintaining the stemness of hematopoietic stem and progenitor cells [10]. Ech A is also beneficial for human CD34+ progenitor cells from peripheral blood to maintain their self-renewal potential and function during ex vivo expansion. The efficacy of Ech A in a model of hemorrhagic and ischemic stroke in rats has been demonstrated [11,12]. It was found that the drug can cross the blood–brain barrier into the cerebrospinal fluid. Ech A also exhibited an antidiabetic effect due to antioxidant and hypoglycemic activities [13,14]. A study demonstrated that Ech A inhibits acetylcholinesterase and exhibits dose-dependent antiradical activity against nitric oxide, which opens up its possible use in the treatment of neurodegenerative diseases [15]. The therapeutic potential of Ech A in the treatment of various inflammatory diseases has been demonstrated in numerous studies: in a model of experimental colitis in mice [16], in an experimental model of bleomycin-induced pneumonia in immature rats [17], in children aged 7–12 years old with chronic inflammatory lung diseases [18,19], and in adolescents with erosive gastroduodenitis [20,21].

Since the search for active compounds and the creation of new drugs is a long, expensive, and risky process, one of the main modern pharmaceutical strategies is the use of registered drugs for a new medical application. Considering the above variety of activities possessed by Ech A with an established mechanism of action, the development of new dosage forms based on this substance seems promising.

To ensure the quality of active pharmaceutical substances and finished drug products, impurities must be monitored carefully during process development, optimization, and changeover. The isolation, characterization, and control of impurities in pharmaceutical substances are being reviewed with a greater focus on national regulatory and international guidelines [22]. According to International Conference on Harmonization (ICH) guidance Q3A(R2) and Q3B(R2), degradation products are impurities resulting from a chemical change in the drug substance during manufacture and/or storage of the drug product due to the effect of light, temperature, pH, water, or reaction with an excipient and/or the immediate container closure system. Due to the presence of a large number of phenolic hydroxyls, Ech A readily undergoes oxidative decomposition. The aim of this study was to isolate and determine the structure of Ech A degradation products formed during the oxidation of its preparation (Histochrome) by O_2_ in air-equilibrated aqueous solutions.

## 2. Results and Discussion

### 2.1. Isolation and Structure Elucidation of Echinochrome A Oxidative Degradation Products

According to our observations, in dry form and in the absence of oxygen in solution, the pharmaceutical substance Ech A remains stable for several years. This was confirmed by us after a 3 year stability study of the Ech A substance and Histochrome preparation in ampoules closed under inert conditions. In aqueous solutions, the Ech A sodium derivative (Histochrome) readily hydrolyzes and oxidizes (Figure S2, Supplementary Materials). Therefore, to provide the opportunity to establish primary oxidation products, we did not use the onerous conditions recommended in the ICH guidelines for degradation product studies. In this work, we studied the oxidation of Ech A sodium derivative in air-equilibrated aqueous solutions.

An HPLC method coupled with diode-array detection (DAD) and mass spectrometry (MS) was developed and validated to monitor the degradation of Ech A and to support the peak identification procedure. The eluent system consisting of H_2_O (A)/MeCN (B) with the addition of 0.2% AcOH in a gradient mode provided acceptable separation of Ech A and its oxidation products. The developed LC method demonstrated good linearity, and the correlation coefficient for Ech A was found to be 0.9987. The limit of detection (LOD) and limit of quantification (LOQ) of Ech A were found to be 22 and 72 ng/mL, respectively. The analytical area of this method was established by the range of experimental data satisfying the linear model. For Ech A (**1**), the corresponding range was determined to be 72–600 ng/mL. The accuracy and reproducibility of the quantification procedure was evaluated according to the results obtained for Ech A, shown in Table S1 (Supplementary Materials). The detection wavelength 254 nm was chosen on the basis of the fact that all target compounds display intense absorption in the region of 230–270 nm.

**Figure 2:**
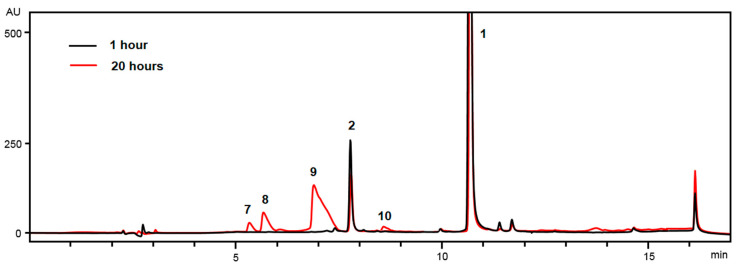
HPLC profiles of Histochrome (Ech A) oxidation products. Unmarked peaks are natural impurities of the Ech A substance [24].

A solution of Ech A sodium derivative from an ampoule was diluted approximately 50-fold with distilled water saturated with atmospheric oxygen, pH 7.2. In this solution, the molar ratio of Ech A to dissolved O_2_ was 3:1. HPLC–DAD–MS analysis showed that, after 1 h in the air-equilibrated aqueous solution, the first oxidation product of Ech A (**1**) was compound **2,** with a retention time of 7.79 min (Figure 2). The high-resolution electrospray ionization mass spectrum (HR-ESI-MS) of compound **2** presented a peak at *m/z* [M − H]^−^ 299.0399 (calculated for [C_12_H_11_O_9_]^−^ 299.0409). The increase in molecular weight of 34 Da compared to Ech A indicates that compound **2** contained two additional hydroxyl groups in the molecule. In its absorption spectrum, there was no absorption band at 468 nm that is characteristic of Ech A, and absorption bands at 256, 321, and 391 nm were present, indicating a decrease in the length of the conjugation chain in the molecule and, therefore, that oxidative changes affected the quinonoid ring of Ech A. According to NMR data, the structure of compound **2** was previously established by us as 7-ethyl-2,2,3,3,5,6,8-heptahydroxy-2,3-dihydro-1,4-naphthoquinone (Figures S12–S17, Supplementary Materials) [23].

The presence of four aliphatic hydroxyl groups was confirmed by the preparation of tetramethyl ether of compound **2** (*m/z* [M − H]^−^ 355.1035, calculated for C_16_H_19_O_9_^−^ 355.1029) by methylation with methyl iodide according to the procedure in [25] (Figure S3, Table S2, Supplementary Materials), which also confirmed the structure of bis-*gem*-diol for compound **2**.

**Figure 3:**
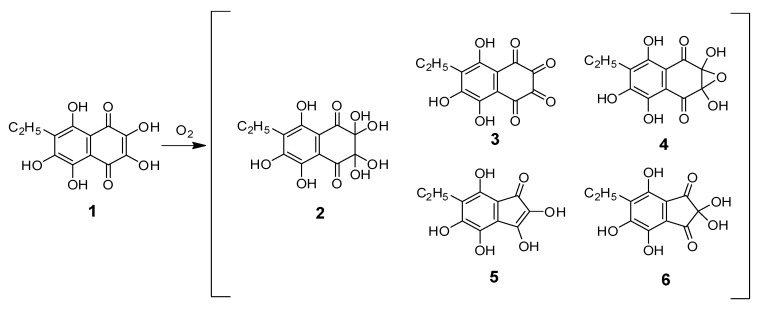
Primary oxidation products of Ech A (**1**); structures **3**–**6** were proposed on the basis of HR-ESI-MS data.

It is interesting to note that, in the mass spectrum of the chromatographic peak with a retention time of 7.79 min, along with the main peak at *m/z* [M − H]^−^ 299, there were low-intensity peaks with mass values *m/z* [M − H]^−^ 263, 281, 237, and 253. These peaks were observed in all cases when the ESI mass spectrum of compound **2** was obtained. On the basis of HR-ESI-MS (Table 1), structures of compounds **3**–**6** were predicted (Figure 3).

**Figure 4:**
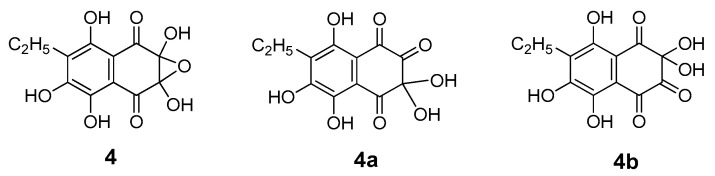
Structures of Ech A oxidation products with brutto-formula C_12_H_10_O_8_.

Prior to our studies, according to published data, the first oxidation product of Ech A was attributed to structures such as the dihydrate of 5,6,8-trihydroxy-7-ethyl-1,2,3,4-tetrahydronaphthalene-1,2,3,4-tetraone (**3**) [26] or monohydrate of 2,3-dihydro-2,3,5,6,8-pentahydroxy-2,3-epoxy-7-ethyl-1,4-naphthoquinone (**4**) [27]. These compounds were present in combination with compound **2**. A compound with the brutto-formula C_12_H_10_O_8_ can exist both in the form of structure **4** and in the form of keto-*gem*-diols **4a** and **4b** (Figure 4). The presence of those compounds in the mixture of oxidation products was previously recorded by us using ^1^H- and ^13^C-NMR spectroscopy [23].

For compounds **5** and **6**, structures 2,3,4,5,7-pentahydroxy-6-ethylinden-1-one and 2,2,4,5,7-pentahydroxy-6-ethylindane-1,3-dione were predicted, respectively. It is known that di- and polycarbonyl vicinal compounds are prone to hydration; therefore, they are often isolated in the form of *gem*-diols, and, in our case, compound **2** was predominant.

Additionally, structures of compounds **3**–**6** were confirmed by obtaining their methyl derivatives by methylation with both methyl iodide and diazomethane, the MS data for which are provided in Tables S2 and S3 (Supplementary Materials).

Compounds **3**–**6** were extremely reactive, as their formation was accompanied by the formation of an intermediate ene-diol radical, superoxide anion radical, hydroxyl radical, and hydrogen peroxide. It was not possible to isolate them from the mixture of Ech A oxidation products, and they continued the chain reaction of oxidation of bis-*gem*-diol **2**, even if O_2_ was removed from the reaction medium. In the process of developing technology for the preparation of a Histochrome for injections (0.2 mg/mL Ech A), we observed that even a small amount of O_2_ entering the drug solution in sealed ampoules led to the appearance of product **2**. Even the subsequent use of an inert medium (argon) did not stop the process of Ech A oxidation, which led to the formation of products **2**–**10** and continued until the complete discoloration of the red-brown Histochrome solution.

**Figure 5:**
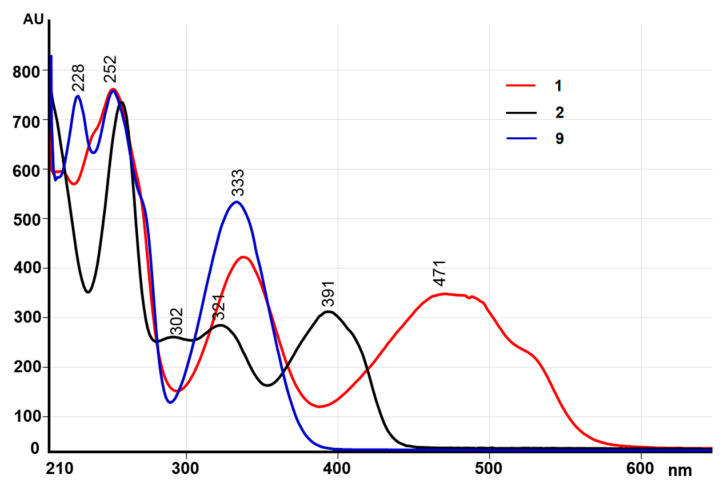
Absorption spectra of Ech A (**1**) and its oxidation products **2** and **9**.

Twenty hours after the start of the reaction, the opaque dark red solution became transparent yellow red. HPLC–MS analysis showed that approximately 50% of Ech A was consumed during this time (Figure 2 and Figure S2, Supplementary Materials). Unreacted Ech A was removed from the aqueous solution by extraction with chloroform, and the oxidation products were extracted with ethyl acetate. Low-pressure reversed-phase chromatography on Toyopearl HW-40 gel of ethyl acetate extraction revealed five oxidation products of Ech A with retention times of 7.79 (**2**), 5.32 (**7**), 5.67 (**8**), 6.89 (**9**), and 8.56 (**10**) min (Table 2). The absorption spectra of compounds **7**–**9** contained absorption bands due to π → π* transitions in the benzenoid core in the region of 310–370 nm, but there were no absorption bands associated with π → π* transitions in the quinonoid core in the region of 460–540 nm, which indicated a cleavage of the quinonoid ring (Table 2, Figure 5).

**Figure 6:**
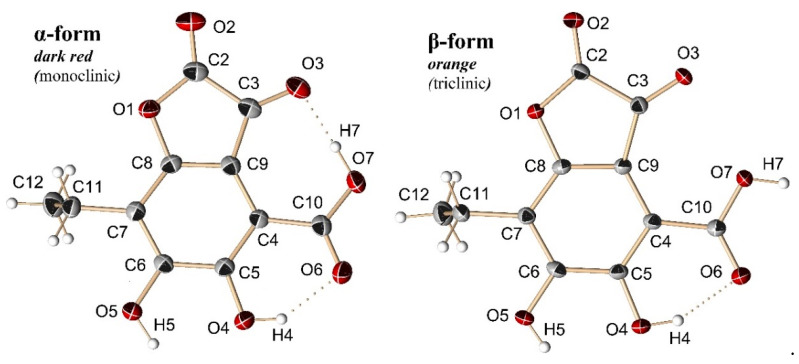
Molecular structure of echinolactone (**11**) in different crystal types (hydrogen bonds are shown as dotted lines).

It turned out that compound **7** was quite unstable in an acidic environment, and, after vacuum evaporation in fractions with compound **7**, product **11** was formed. According to the ESI-MS spectrum, product **11** had an *m/z* 251 [M − H]^−^, which was 18 Da less than mass of compound **7**. The absorption band at 385 nm in the absorption spectrum of compound **11** indicated a longer π → π* transition chain in its molecule compared to compound **7**. In the ^1^H-NMR spectrum of compound **11**, we observed a triplet (δ_H_ 1.24) and a quartet (δ_H_ 2.78) of an ethyl substituent, a broadened singlet of two hydroxyl groups (δ_H_ 5.33), and a singlet of the hydroxyl group bound to carbonyl (δ_H_ 12.84) (Figure S18, Supplementary Materials). The ^13^C-NMR spectrum of compound **11** contained 11 carbon signals: two signals for the ethyl group (δ_C_ 12.8 and 17.2), three quaternary carbons (δ_C_ 106.3, 108.2, and 121.4), and seven quaternary carbons bound to oxygen (δ_C_ 151.2, 158.0, 159.9, 161.6, 171.0, and 177.8) (Figure S19, Supplementary Materials). In the HMBC spectrum of compound **11**, protons of the ethyl group (δ_H_ 2.78) showed correlations with δ_C_ 121.4, 159.0, and 161.6 (Figure S20, Supplementary Materials). These spectral data were insufficient to establish the structure of **11**; however, it turned out that compound **11** easily formed crystals and, as such, X-ray analysis was used to establish its structure. Compound **11** showed polymorphism, with two types of crystals obtained from the same system of solvents (EtOH–CHCl_3_ = 1:5 *v*/*v*). Recrystallization from these solvents simultaneously provided crystals as dark-red plates (α-form) and as orange prisms (β-form) (Figure 6). Red crystals were predominant (about 90%). Both crystal forms were a crystalline hydrate of **11** (Tables S4–S6, Supplementary Materials).

The α-form of **11** crystallized as monoclinic system with the space group *P*2_1_/*c* and cell parameters *a* = 4.7823(6) Å, *b* = 7.9520(9) Å, *c* = 14.4705(17) Å, and *Z* = 4, and the final *R*-value was found to be 0.0512 (Table S4, Supplementary Materials). The β-form of **11** crystallized as a triclinic system with the space group *P*ī and cell parameters *a* = 4.7823(6) Å, *b* = 7.9520(9) Å, *c* = 14.4705(17) Å, and *Z* = 2, and the final *R*-value was found to be 0.0407 (Table S4, Supplementary Materials).

In the α- and β-forms of molecule **11**, all atoms with the exception of the CH_3_ carbon atom of the ethyl group C12 were located in the same plane (Figure 6). The deviation from the plane did not exceed 0.138(2) Å. The main difference between the two forms was the dimensional orientation of the hydrogen atom H7 of the carboxyl group, which allowed us to consider the molecules of α- and β-forms as stereoisomers (Figure 6); in the α-form, this atom participates in the formation of an intramolecular hydrogen bond, while, in the β-form, it participates in an intermolecular hydrogen bond. In both crystalline forms of **11**, all the corresponding C–C and C–O bonds had close values (Table S5, Supplementary Materials). The torsion angles of C6–C7–C11–C12 in the α- and β-forms were 96.3(3)° and 83.4(2)°, respectively. The intermolecular hydrogen bonds of molecule **11** with H_2_O molecules played a decisive role in the formation of crystalline structures. In the α-form, the H_2_O molecules were linked to each other by O*w*–H∙∙∙O*w* hydrogen bonds in an infinite chain along the [0 0 1] direction, and they combined isolated **11** molecules into a three-dimensional framework (Figure S21, Supplementary Materials). The coordination number of O atoms of H_2_O molecules in α-C_11_H_8_O_7_∙H_2_O was 4. In the β-form, molecules of **11** were joined by O7–H7∙∙∙O3 bonds in pairs into a centrosymmetric bimolecular associate, and H_2_O molecules distributed their hydrogen bonds only between molecules of **11**, combining pairs into flat ribbons that were infinite along the [1 –1 0] direction (Figure S22, Supplementary Materials). Ribbons were packed in corrugated layers parallel to the plane [0 0 1] (Figure S23, Supplementary Materials). The coordination number of O atoms of H_2_O molecules in β-C_11_H_8_O_7_∙H_2_O was 3. The triclinic β-form of C_11_H_8_O_7_∙H_2_O had a slightly higher density (1.664 g/cm^3^) at a temperature of T = 173(2) K than the monoclinic α-form (1.642 g/cm^3^) and could formally be considered as more stable.

On the basis of X-ray data, **11** was assigned the structure of 7-ethyl-5,6-dihydroxy-2,3-dioxo-2,3-dihydrobenzofuran-4-carboxylic acid, and this compound was named echinolactone.

Compounds **7** and **8** turned out to be unstable under conditions of repeated chromatographic separation; therefore, to establish their structures, their stable methyl derivatives with *m*/*z* [M − H]^−^ 297 and 239, respectively, were obtained by methylation with diazomethane.

**Figure 7:**
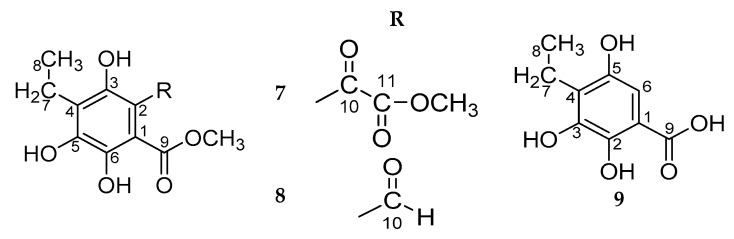
Structures of dimethyl ether of compound **7**, methyl ether of compound **8**, and of compound **9**.

On the basis of the ^1^H- and ^13^C-NMR spectra of the dimethyl ether of compound **7**, methyl ether of compound **8,** and of compound **9** (Table 3, Figure 7), it could be concluded that these compounds retained the same substituents as the Ech A benzenoid ring: an ethyl substituent, a free hydroxyl group, and hydroxyl groups, the protons of which were bound by an intramolecular hydrogen bond with the corresponding carbonyl groups, but the carboxyl group appeared. The differences in the NMR spectra of the Ech A oxidation products consisted of the chemical shifts of the substituents next to the carboxyl group (Table 3); therefore, to establish the structure of compounds **7**–**9**, it was necessary to establish the nature of these substituents.

In the dimethyl ether of compound **7**, the proton of the hydroxyl group at C-6 (δ_H_ 10.49) was hydrogen-bonded to the carbonyl of the ester group at C-9 (δ_H_ 169.1), and the proton of the hydroxyl group at C-3 (δ_H_ 11.30) was hydrogen-bonded to the carbonyl of the methylcarboxy group at C-10 (δ_C_ 186.8) (Figures S24–S26, Supplementary Materials). The signal at δ_C_ 162.9 ppm in the ^13^C-NMR spectrum corresponded to the carbonyl of the ester group of the methylcarboxy fragment. Two singlets with an integrated intensity of 3H each at δ_H_ 3.82 and 3.88 in the ^1^H-NMR spectrum corresponded to the protons of methoxy groups at C-9 and C-11. In the ^13^C-NMR spectrum of compound **7** dimethyl ether, there were two corresponding signals at δ_C_ 52.2 and 53.0 ppm. According to an analysis of the NMR spectra of the dimethyl derivative, the structure of compound **7** was established as 4-ethyl-3,5,6-trihydroxy-2-oxalobenzoic acid.

The ^13^C-NMR spectrum of methyl ether of compound **8** contained a signal in a low field at δ_C_ 195.2, the chemical shift of which was characteristic for the aldehyde carbon atom (Table 3, Figure S28, Supplementary Materials). The singlet at δ_H_ 10.43 in the ^1^H-NMR spectrum of this compound corresponded to the proton of the aldehyde group. According to NMR data of its methyl derivative (Figures S27–S34, Supplementary Materials), the structure of compound **8** was established as 4-ethyl-2-formyl-3,5,6-trihydroxybenzoic acid.

The ^1^H-NMR spectrum of compound **9** contained a singlet signal of the aromatic proton at C-6 (δ_H_ 6.88), which corresponded to a signal at δ_C_ 104.8 ppm in the ^13^C-NMR spectrum (Table 3, Figures S35 and S36, Supplementary Materials). The chemical shift of the proton of the hydroxyl group at C-5 (δ_H_ 7.54) indicated that it was not bound by an intramolecular hydrogen bond as in compound **8**. Thus, the structure of compound **9** was established as 4-ethyl-2,3,5-trihydroxybenzoic acid (Figures S35–S39, Supplementary Materials).

On the basis of the NMR data of compound **10** and its methyl derivative (Figures S40–S48, Supplementary Materials), the structure of **10** was established as 3-ethyl-2,5-dihydroxy-1,4-benzoquinone. This compound was previously described by Moore et al. as a natural pigment of sea urchins of the genus *Echinothrix* [28]. However, it is likely that compound **10** was one of the most stable oxidation products of Ech A obtained by the authors during the storage and repeated chromatographic separation of sea urchin extracts. According to the conditions of our experiment, the oxidation process was stopped when half of the Ech A was oxidized; thus, a very small amount of **10** was isolated.

### 2.2. Proposed Mechanism of Echinochrome A Oxidative Degradation

**Figure 8:**
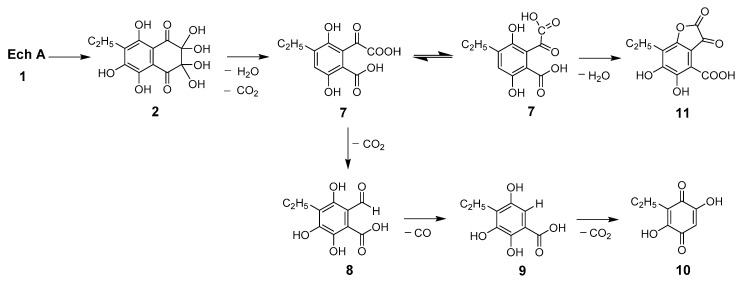
Proposed scheme of the Ech A oxidative degradation process.

Many bioactive natural and pharmaceutical compounds that are α-hydroxyketones such as oxolin, ascorbate, glyoxal, and cyclic ketones are susceptible to autooxidation [29]. It has been shown that α-hydroxyketones auto-oxidize under physiological conditions via the enediol tautomer [30,31]. One condition which favors the formation of the enediol is the presence of a vicinal carbonyl group. The equilibrium is generally displaced in favor of the more thermodynamically stable ketol tautomer. The autooxidation of such enediols, as well as tetrahydroxy-l,4-benzoquinone, ascorbate, and Ech A, has been shown to involve the generation of intermediates such as carbon-centered free radicals and ROS including the superoxide radical anion, hydroxyl radical, and hydrogen peroxide [23,30,32,33]. The mechanisms of the primary attack of triplet and singlet oxygen molecules on Ech A (**1**), the result of which is bis-*gem*-diol **2** formation, were described in detail in previous studies [23,34]. Here, according to the structures of the isolated products, we propose the scheme of the further Ech A oxidative degradation process (Figure 8).

Pracht et al. showed that splitting of the aromatic system of phenolic substances occurs only if the first oxidation stage includes the formation of *o*-quinone [35]. Subsequent cleavage of the ring structure can occur between two keto groups, as in our case, for example, in compounds **3**, **4**, **4a**, and **4b**, which always presented together with compound **2** (Figure 3 and Figure 4). It can be assumed that ring rupture in the primary oxidation product **2** with loss of H_2_O and CO_2_ led to the formation of phthalonic acid derivative **7**. Compound **7**, resulting from keto–enol tautomerism, gave echinolactone **11** upon dehydration in an acidic medium and heating during the evaporation process. Establishment of the structure of **11** using X-ray analysis played an important role in the elucidation of the structure of the labile compound **7**. Further sequential decarboxylation of **7** resulted in the formation of **8**, a benzoic acid derivative with an aldehyde group, and a benzoic acid derivative **9**. Decarboxylation of **9** and subsequent hydration and oxidation led to the formation of stable benzoquinone **10**.

Thus, it was shown that the oxidative destruction of Ech A did not affect the benzenoid fragment of its molecule. All transformations occurred only in the quinonoid ring with the formation of bis-*gem*-diol **2**, further oxidation of which occurred upon cleavage of the dihydroquinonoid ring and led to the formation of derivatives of phthalonic (**7**) and benzoic (**8**, **9**) acids, as well as benzoquinone **10**.

### 2.3. Predicted Toxicity of Echinochrome A and Its Oxidation Products

Obtaining information on the toxicity of compounds and their impurities is an important part of the drug design development process. However, for impurities in particular, this information cannot be obtained experimentally. In this case, in silico studies assist in evaluating the results.

The potential toxicity of Ech A and its oxidative degradation products was assessed with the webserver ProTox-II. This virtual lab predicts the toxicity of small molecules on the basis of a total of 33 models for the prediction of various toxicity endpoints such as acute toxicity, hepatotoxicity, cytotoxicity, carcinogenicity, mutagenicity, immunotoxicity, adverse outcome (Tox21) pathways, and toxicity targets [36].

The results of the predicted toxicity of the original Ech A molecule and its oxidative degradation products are shown in Table 4.

The predicted LD_50_ value for the first major oxidation product **2** was 221 mg/kg (toxicity class III), and the predicted acute toxicity for other oxidation products **7**–**10** was much lower (≥2000 mg/kg, toxicity class IV–V), suggesting they cannot lead to serious toxic effects.

**Figure 9:**
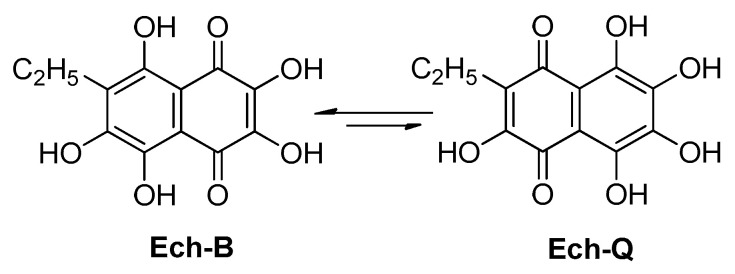
Tautomeric forms of Ech A: Ech-B (ethyl in the benzene ring) and Ech-Q (ethyl in the quinonoid ring).

As shown above, structures **2** and **7**–**10** were unambiguously defined as nontoxic and there was no doubt about the predicted toxicity results for these compounds. However, for derivatives of the naphthazarin (5,8-dihydroxy-1,4-naphthoquinone) structure, to which Ech A belongs, it is not so simple. Under various conditions, Ech A (**1**) can exist as a mixture of four tautomeric forms; however, only the 1,4-naphthoquinonoid forms Ech-B (ethyl in the benzene ring) and Ech-Q (ethyl in the quinonoid ring) are energetically favorable (Figure 9) [23,37,38].

As seen in Table 4, the Ech-B and Ech-Q formulas loaded in ProTox-II showed dramatically different results for acute toxicity. For the Ech-B form, a toxic LD_50_ of 16 mg/kg and toxicity class II were predicted; for Ech-Q, the LD_50_ was 487 mg/kg and the toxicity class IV was predicted. According to our experimental data for the determination of intraperitoneal acute toxicity of the Ech A substance in outbred mice, the LD_50_ was found to be 153.7 mg/kg (Table S7, Supplementary Materials) and toxicity class III was determined, which was somewhere between the predicted values for Ech-B and Ech-Q, confirming the benzenoid-quinonoid equilibrium of Ech A. However, the structure of bis-*gem*-diol **2** and of other products **3**–**6** indicated that only one of the possible tautomeric forms (Ech-B) of Ech A was involved in the oxidation process.

We established the experimental cytotoxicity for two compounds, Ech A (**1**) and bis-*gem*-diol **2**. The cytotoxicity of **1** and **2** was estimated by methylthiazolyltetrazolium bromide (MTT) assay using pig embryo kidney (PK) cells and African green monkey kidney (Vero) cells. For Ech A (**1**), 50% inhibition of cell viability was observed at 54.4 mkg/mL and 60.5 mkg/mL in PK and Vero cell lines, respectively [39]. For compound **2**, this value was found to be 140 mkg/mL in Vero cells; thus, it had a weaker toxic effect on normal cells.

With confidence scores of 0.77 and 0.82 for Ech-B and Ech-Q, respectively, it was predicted that these compounds had mutagenic activity (Table 4). However, as shown in a comprehensive study of the mutagenic properties of the Histochrome drug carried out in accordance with the requirements of the Pharmacological Committee of the Russian Ministry of Health, Histochrome in the range of 1.0–10 mg/kg does not have the ability to induce chromosomal damage in the bone marrow cells of C57BL/6 mice, nor does it lead to an increase in the level of spontaneous gene mutations in *Drosophila* or induce gene mutations in *Salmonella typhimurium* (Tables S8–S10, Supplementary Materials). These results allowed us to conclude that Ech A does not exhibit mutagenic activity, at least in the range of therapeutic doses. However, there is published evidence of mutagenic activity of Ech A [33]. In this publication, the source of the drug was not clearly indicated, and neither were its preparation method or purity. This once again confirms that the standardization of drug substances is very important. For the implementation of its medicinal properties, not only the structure of the active substance is important, but also the properties of the drug, determined by the technological process used for its production.

## 3. Materials and Methods

### 3.1. Materials

Drug substance echinochrome A and drug product Histochrome were produced by G.B. Elyakov Pacific Institute of Bioorganic Chemistry (Vladivostok, Russia). TSKgel Toyopearl HW-40 (TOYO SODA, Tokyo, Japan), and Sephadex LH-20 (GE Healthcare Bio-Sciences AB, Uppsala, Sweden) were used for column chromatography. HPLC-grade water and acetic acid were purchased from Panreac Quimica (Barcelona, Spain). MeCN grade 0 was sourced from Cryochrom (Saint Petersburg, Russia). Other solvents used in this study were of analytical grade. Deuterated solvents acetone-*d*_6_, CDCl_3_, CD_3_CN, and Bruker^®^ SampleJet NMR tubes WIMWG10007SJ (178 mm, cap, O.D. 5.0 mm) for NMR experiments were purchased from Sigma (St. Louis, MO, USA).

### 3.2. Instruments

Ultraviolet (UV) spectra were recorded using a UV 1800 spectrophotometer (Shimadzu USA Manufacturing Inc., Canby, OR, USA) and infrared (IR) spectra were obtained on an Equinox 55 Fourier-transform (FT) IR spectrophotometer (Bruker, Rheinstetten, Germany). HR-ESI-MS experiments were carried out using a Shimadzu hybrid ion trap time-of-flight mass spectrometer (Kyoto, Japan). The operating settings of the instrument were as follows: electrospray ionization (ESI) source potential, −3.8 and 4.5 kV for negative and positive polarity ionization, respectively; drying gas (N_2_) pressure, 200 kPa; nebulizer gas (N_2_) flow, 1.5 L/min; temperature for the curved desolvation line (CDL) and heat block, 200 °C; detector voltage, 1.5 kV; range of detection, 100–900 *m/z*. The mass accuracy was below 4 ppm. The data were acquired and processed using Shimadzu LCMS Solution software (v.3.60.361). The ^1^H-, ^13^C-, and two-dimensional (2D) NMR spectra were recorded using NMR Bruker AVANCE III DRX-700, AVANCE DRX-500, and AVANCE DPX-300 instruments (Bruker, Karlsruhe, Germany). The chemical shift values (δ) and the coupling constants (*J*) are given in parts per million and in Hz, respectively. HMBC spectra were optimized for 5 Hz coupling. The key NMR acquisition parameters are shown in the Supplementary Materials.

The X-ray experiments for single crystals were performed using a Bruker SMART-1000 Charge-Coupled Detector (CCD) diffractometer (MoK_α_-radiation, graphite monochromator). Intensity data were corrected for absorption using the multi-scan method. The structures were solved using the direct methods and refined by the least-squares calculation in anisotropic approximation for non-hydrogen atoms. Hydrogen atoms of ethyl groups after checking their presence in a difference map were placed in geometrically idealized positions and refined in the riding-model approximation. Hydrogen atoms of water molecules and hydroxyl groups were located in the difference Fourier maps and refined with U_iso_(H) = 1.5U_eq_ (O). Data collection and editing, as well as refinement of unit cell parameters, were performed with the SMART [40] and SAINT [41] program packages. All calculations on the determination and refinement of the structures were carried out using the SHELXTL/PC software [42,43].

### 3.3. HPLC–DAD–MS Analysis

HPLC–DAD–MS was performed using a system consisting of a CBM-20A system controller (Shimadzu USA Manufacturing Inc., Canby, OR, USA), two LC-20 CE pumps (Shimadzu USA Manufacturing Inc., Canby, OR, USA), a DGU-20A3 degasser (Shimadzu Corp., Kyoto, Japan), an SIL-20A autosampler (Shimadzu USA Manufacturing Inc., Canby, OR, USA), a diode-matrix SPD-M20A (Shimadzu USA Manufacturing Inc., Canby, OR, USA), and a mass-spectrometric detector LCMS-2020 (Shimadzu Corp., Kyoto, Japan). The separation was carried out on a Discovery HS C18 column (150 × 2.1 mm, 3 µm particle size, Supelco, Bellefonte, PA, USA) with a Supelguard Ascentis C18 pre-column (2 × 2.1 mm, 3 µm particle size, Supelco, Bellefonte, PA, USA) using a binary gradient of H_2_O (A)/MeCN (B) with the addition of 0.2% AcOH at a flow rate of 0.2 mL/min and column temperature of 40 °C. The gradient was as follows: 0–6 min, 10–40% (B); 6–11 min, 40–100% (B); 11–12 min, 100% (B), 12–13 min, 100–10% (B); 13–17 min, 10% (B). The chromatograms were recorded at 254 nm. Mass spectra were taken in the electrospray ionization (ESI) mode at atmospheric pressure, recording negative ions (1.50 kV) in the *m/z* range of 100–900 with the following settings: drying gas, N_2_ (10 L/min); nebulizer gas N_2_ flow, 1.5 L/min; temperature for the curved desolvation line (CDL), 200 °C; temperature of heat block, 250 °C; interface voltage, 3.5 kV. Before analysis, samples were filtered through a 0.2 µm polytetrafluoroethylene (PTFE) syringe filter. The injection volume was 5 µL.

### 3.4. HPLC Method Validation

Linearity of the method was established by using methanolic Ech A solutions containing 50–1500 ng/mL. Each linearity sample was injected in triplicate. The calibration curve was constructed as linear regression analysis of the peak area versus concentration. The limits of detection (LOD) and quantification (LOQ) of Ech A were calculated as concentrations at which the signal-to-noise ratio was below 3 and 10, respectively. The accuracy of the method was established by recovery studies of Ech A samples (100, 250, 500 ng/mL); data are provided in the Supplementary Materials (Table S1). Accuracy was expressed as relative standard deviations (RSDs) and recoveries (%). Selectivity was confirmed through peak purity studies using a DAD detector.

### 3.5. Oxidation Products Preparation and Isolation

Histochrome from an ampoule (10 mg/mL Ech A, 5 mL) was diluted with distilled water (250 mL), balanced with atmospheric oxygen, to obtain a solution with an Ech A concentration of 0.2 mg/mL, pH 7.2. The oxidation was carried out in the light without stirring at ambient temperature. The process was monitored by HPLC–DAD–MS. When approximately 50% of the Ech A was consumed (after 20 h), the reaction was stopped by adding hydrochloric acid to pH 2. Unreacted Ech A was removed from solution by extraction with chloroform. The oxidation products were extracted with ethyl acetate, the solvent was removed under reduced pressure, and the residue was chromatographed on a Toyopearl HW-40 TSKgel column in a gradient of 10–50% ethanol containing 0.5% formic acid. From fractions eluted with 10–30% ethanol compounds were extracted with ethyl acetate and the solvent was evaporated. As a result, compounds **2**, **9**, and **10** were isolated. Fractions eluted with 30–50% ethanol were concentrated in vacuo at 50 °C; as a result, echinolactone **11** was obtained from the fraction of compound 7.

A small portion of oxidation products (10 mg) was dissolved in cold MeOH and treated with diazomethane in diethyl ether on ice as described [44]. The reaction process was controlled by HPLC–DAD–MS. After 1 h, the solvent was removed in vacuo, and the residue was subjected to column chromatography on Sephadex LH-20 eluting with CHCl_3_/EtOH 8:1. As a result, dimethyl ether of **7** and methyl ether of **8** were obtained.

*7-ethyl-2,2,3,3,5,6,8-heptahydroxy-2,3-dihydro-1,4-naphthoquinone* (**2**): C_12_H_12_O_9_; UV (ethanol) λ_max_ 256, 320, 391 nm; ESI-MS *m/z*: 299 [M − H]^−^; HR-ESI-MS *m/z*: 299.0399 [M − H]^−^ (calculated for [C_12_H_11_O_9_]^−^ 299.0409).

*4-ethyl-3,5,6-trihydroxy-2-oxalobenzoic acid* (**7**); C_11_H_10_O_8_; UV (CH_3_CN–H_2_O) λ_max_ 219, 271, 320 nm; ESI-MS *m/z*: 269 [M − H]^−^; HR-ESI-MS *m/z*: 269.0304 [M − H]^−^ (calculated for [C_11_H_9_O_8_]^−^ 269.0297).

*Dimethyl ether of compound***7**: light-yellow powder; C_13_H_14_O_8_; UV (CH_3_CN–H_2_O) λ_max_ 224, 250, 324, 384 nm; IR (CDCl_3_) ν_max_ 3520, 3156, 2975, 2956, 2937, 2878, 2855, 1734, 1683, 1632, 1595 cm^−1^; ESI-MS *m/z*: 297 [M − H]^−^; ^1^H- and ^13^C-NMR (see Table 3).

*4-ethyl-2-formyl-3,5,6-trihydroxybenzoic acid* (**8**); C_10_H_10_O_6_; light-yellow powder; UV (CH_3_CN–H_2_O) λ_max_ 270, 320 nm. ESI-MS *m*/*z*: 225 [M − H]^−^; HR-ESI-MS *m/z*: 225.0405 [M − H]^−^ (calculated for [C_10_H_9_O_6_]^−^ 225.0399).

*Methyl ether of compound***8**: C_11_H_12_O_6_; light-yellow powder; UV (CH_3_CN–H_2_O) λ_max_ 238, 303, 369 nm; IR ν_max_ (CDCl_3_) 3011, 2959, 2935, 2878, 2865, 1668, 1634, 1611 cm^−1^. ESI-MS *m*/*z*: 239 [M − H]^−^; HR-ESI-MS *m/z*: 239.0570 [M − H]^−^ (calculated for [C_11_H_11_O_6_]^−^ 239.0555); ^1^H- and ^13^C-NMR (see Table 3).

*4-ethyl-2,3,5-trihydroxybenzoic acid* (**9**); C_9_H_10_O_5_; light-yellow powder; UV (CH_3_CN–H_2_O) λ_max_ 228, 251, 333 nm; IR ν_max_ (CCl_4_) 3352, 2960, 2928, 2875, 2855, 1642 cm^−1^. ESI-MS *m*/*z*: 197 [M − H]^−^; HR-ESI-MS *m/z*: 197.0452 [M − H]^−^ (calculated for [C_9_H_9_O_5_]^−^ 197.0450); ^1^H- and ^13^C-NMR (see Table 3).

*3-ethyl-2,5-dihydroxy-1,4-benzoquinone* (**10**); C_8_H_8_O_4_; light-yellow powder; UV (CH_3_CN–H_2_O) λ_max_ 228 nm, literature 228 nm [28]; IR ν_max_ (CDCl_3_) 3366, 2976, 2935, 2878, 1731, 1641 cm^−1^. ESI-MS *m*/*z*: 167 [M − H]^−^; HR-ESI-MS *m*/*z*: 167.0343 [M − H]^−^ (calculated for [C_8_H_7_O_4_]^−^ 167.0344). ^1^H-NMR (700 MHz, CDCl_3_) δ, ppm (*J*, Hz): 1.21 (3H, t, *J* = 7.5, CH_3_), 2.49 (2H, q, *J* = 7.5, CH_2_), 6.01 (1H, s, H), 7.54 (2H, s, OH); ^1^H- and ^13^C-NMR data of dimethyl ether of **10** (see Figures S43–S48, Supplementary Materials).

*Echinolactone* (7-ethyl-5,6-dihydroxy-2,3-dioxo-2,3-dihydrobenzofuran-4-carboxylic acid, **11**): C_11_H_8_O_7_; UV (ethanol) λ_max_ (log*ε* ): 215 (3.4), 331 (2.8), 385 (2.8) nm; IR ν_max_ (CDCl_3_) 3483, 1838, 1698, 1581 cm^−1^; ESI-MS *m*/*z*: 251 [M − H]^−^; EI-MS *m*/*z*: 252 [M]^+^. ^1^H-NMR (300 MHz, CDCl_3_), δ, ppm: 1.24 (3H, t, *J* = 7.5, CH_3_), 2.78 (2H, dd, *J* = 7.5, CH_2_), 5.24, (1H, s, OH), 12.88 (1H, s, OH). ^13^C-NMR (75 MHz, CD_3_CN), δ, ppm: 13.3, 17.8, 106.9, 108.8, 121.9, 151.7, 158.6, 160.5, 162.2, 171.5, 178.4.

The main crystal data, data collection, and refinement parameters are presented in Table S4 (Supplementary Materials). The selected geometric parameters of **11** crystal forms are given in Table S5 (Supplementary Materials). The hydrogen-bond geometry parameters are listed in Table S6 (Supplementary Materials). Crystallographic data for the structures in this study were deposited to the Cambridge Crystallographic Data Center as supplementary publication No. CCDC 1,822,816 and 1822821; copies of the data can be obtained, free of charge, via an application to CCDC, 12 Union Road, Cambridge CB2 1EZ, UK, (fax: +44 1223 336,033 or e-mail: deposit@ccdc.cam.ac.uk).

### 3.6. In Silico Toxicity Studies

The potential acute toxicity, hepatotoxicity, carcinogenicity, immunotoxicity, mutagenicity, and cytotoxicity of Ech A and its degradation products via oral administration were assessed using ProTox-II [36].

## 4. Conclusions

In this study, Ech A degradation products formed during oxidation by O_2_ in air-equilibrated aqueous solutions were identified, isolated, and structurally characterized. During the oxidation of Ech A, transformation was found to occur only in the quinonoid ring, with the formation of a hydrogenated quinonoid cycle with two carbonyl groups and two pairs of geminal hydroxyl groups. The further oxidation of bis-*gem*-diol occurred with the cleavage of the dihydroquinonoid ring and a chain of subsequent decarboxylations.

The HPLC method with DAD and MS detection was developed and validated to monitor the Ech A degradation process and to identify the appearing compounds. The structural studies and obtained HPLC–MS parameters of the main Ech A oxidation products are of great interest from the point of view of investigation of the chemical properties of drug substances and for developing methods for monitoring the quality of drugs and food additives obtained from sea urchin pigments, in addition to their stability.

In recent years, there has also been a growing interest in the environmental significance of methods to characterize the destruction of pharmaceutical compounds and study their toxic properties in order to avoid the accumulation of these compounds in nature.

The in silico toxicity studies performed using ProTox-II webserver revealed that Ech A oxidative degradation products do not exhibit mutagenic properties, and their toxicity values were much lower than that of Ech A. This means that the spontaneous formation of these degradation products in preparations using Ech A would not be harmful to patients.

## Figures and Tables

**Table 1:** High-resolution electrospray ionization mass spectrometry (HR-ESI-MS) characteristics of the chromatographic peaks of Ech A oxidation products at retention time 7.79 min.

Compound	Peak Intensity Relative to 2, (%)	Formula	Measured *m/z* [M − H]^−^	Calculated *m/z* [M − H]^−^
**2**	100	C_12_H_12_O_9_	299.0399	299.0409
**3**	3	C_12_H_8_O_7_	263.0192	263.0197
**4**	6	C_12_H_10_O_8_	281.0294	281.0303
**5**	2	C_11_H_10_O_6_	237.0397	237.0405
**6**	3	C_11_H_10_O_7_	253.0356	253.0356

**Table 2:** HPLC coupled with diode array detection (DAD) and MS parameters of Ech A (**1**) and its oxidation products **2** and **7**–**10**.

Compound	Rt (min)	Formula	Measured *m/z* [M − H]^−^	Calculated *m/z* [M − H]^−^	λ_max_ (nm)
**1**	10.71	C_12_H_10_O_7_	265.0352	265.0348	254, 338, 471
**2**	7.79	C_12_H_12_O_9_	299.0399	299.0409	256, 321, 391
**7**	5.32	C_11_H_10_O_8_	269.0304	269.0297	219, 271, 320
**8**	5.67	C_10_H_10_O_6_	225.0405	225.0399	270, 320
**9**	6.89	C_9_H_10_O_5_	197.0452	197.0450	228, 251, 333
**10**	8.56	C_8_H_8_O_4_	167.0343	167.0344	287

**Table 3:** NMR data of dimethyl ether of compound **7** (500 MHz for ^1^H and 126 MHz for ^13^C, δ, ppm, *J*/Hz), methyl ether of compound **8**, and of compound **9** (700 MHz for ^1^H and 176 MHz for ^13^C, δ, ppm, *J*/Hz).

No.	Dimethyl Ether of 7 (CDCl_3_)			Methyl Ether of 8 (Acetone-*d*_6_)			9 (Acetone-*d*_6_)		
	δ_C_	δ_H_	HMBC	δ_C_	δ_H_	HMBC	δ_C_	δ_H_	HMBC
1	107.7			107.2			109.4		
2	106.3			109.3			144.4	10.70 (1H, s, OH)	1, 2
3	158.4	11.30 (1H, s, OH)	2, 3, 4, 5	159.7	13.36 (1H, s, OH)	2, 3, 4	144.5	8.01 (1H, s, OH)	
4	124.0			124.2			126.3		
5	150.7	6.53 (1H, s, OH)	3, 4, 5, 6	150.7	6.56 (1H, s, OH)	4, 5, 6	148.2	7.54 (1H, s, OH)	
6	143.3	10.49 (1H, s, OH)	1, 5, 6	144.9	11.35 (1H, s, OH)	1, 5, 6	104.8	6.88 (1H, s, H)	1, 3, 4, 5, 9
7	16.2	2.76 (2H, q, *J* = 7.5, CH_2_)	3, 4, 5, 8	16.5	2.77 (2H, q, *J* = 7.5, CH_2_)	3, 4, 5, 8	17.7	2.73 (2H, q, *J* = 7.5, CH_2_)	3, 4, 5, 8
8	12.6	1.16 (3H, t, *J* = 7.5, CH_3_)	4, 7	12.5	1.17 (3H, t, *J* = 7.5, CH_3_)	4, 7	13.2	1.12 (3H, t, *J* = 7.5, CH_3_)	
9	169.1			170.6			172.7		
10	186.8			195.2	10.43 (1H, s, COH)	2, 3, 4			
11	162.9								
9-OCH_3_	53.0	3.88 (3H, s, OCH_3_)	1, 9	53.02	4.04 (3H, s, OCH_3_)				
11-OCH_3_	52.2	3.82 (3H, s, OCH_3_)	10, 11						

**Table 4:** Oral toxicity prediction results for Ech A and its degradation products. LD_50_, median lethal dose.

Classification and target	Ech A and its Degradation Products						
	1 (Ech-B)	1 (Ech-Q)	2	7	8	9	10
Predicted toxicity class	II	IV	III	IV	IV	IV	V
Predicted LD_50_, mg/kg	16	487	221	2000	2000	1800	2800
**Organ Toxicity**							
Hepatotoxicity	Inactive	Inactive	Inactive	Inactive	Inactive	Inactive	Inactive
**Toxicity Endpoints**							
Carcinogenicity	Inactive	Inactive	Inactive	Inactive	Inactive	Inactive	Inactive
Immunotoxicity	Inactive	Inactive	Inactive	Inactive	Inactive	Inactive	Inactive
Mutagenicity	Active	Active	Inactive	Inactive	Inactive	Inactive	Inactive
Cytotoxicity	Inactive	Inactive	Inactive	Inactive	Inactive	Inactive	Inactive

## Supplementary Materials

The following are available online: Figure S1. The UV-Vis spectrum of Histochrome (blue) and Echinochrome A (black) in ethanol solution containing 1 mM HCl; Figure S2. Change in the concentration of echinochrome A during the oxidation of a 1% histochrome solution; Table S1. Accuracy and reproducibility of the quantification of echinochrome A (1) using HPLC method; Figure S3. HRESIMS (negative mode) data for methyl ethers of Ech A oxidation products obtained by methylation with methyl iodide; Table S2. HRESIMS (negative mode) data for methyl ethers of Ech A oxidation products obtained by methylation with methyl iodide; Table S3. ESIMS (negative mode) data for methyl ethers of Ech A oxidation products obtained by methylation with diazomethane; Figures S4 and S5. IR spectrum (CDCl3) of compound 7 dimethyl ether; Figures S6 and S7. IR spectrum (CDCl3) of compound 8 methyl ether; Figures S8 and S9. IR spectrum (CDCl3) of compound 10; Figures S10 and S11. IR spectrum (CDCl3) of compound 11; Figure S12. 1H NMR spectrum (300 MHz, acetone-d6) of 7-ethyl-2,2,3,3,5,6,8-heptahydroxy-2,3-dihydro-1,4-naphthoquinone (2); Figure S13. 13C NMR spectrum (75 MHz, acetone-d6) of 2; Figure S14. HMBC spectrum (300 MHz, acetone-d6) of 2; Figures S15–S17. HMBC correlations of 2 (enlarged); Figure S18. 1H NMR spectrum (300 MHz, CDCl3) of echinolactone (11); Figure S19. 13C NMR spectrum (75 MHz, CD3CN) of echinolactone (11); Figure S20. HMBC spectrum (300 MHz, CD3CN) of echinolactone (11); Table S4. Selected crystal data and refinement parameters for α- and β- forms of C11H8O7•H2O; Table S5. Selected geometric parameters (Å) for α- and β- forms of C11H8O7•H2O; Table S6. Hydrogen-bond geometry (Å, °) for α- and β- forms of C11H8O7•H2O; Figure S21. Overall packing for α-C11H8O7•H2O viewed along the a-axis direction; Figure S22. A plot of band for β-C11H8O7•H2O; Figure S23. Overall packing for β-C11H8O7•H2O viewed along the a-axis direction; Figure S24. 1H NMR spectrum (500 MHz, CDCl3) of dimethyl ether of compound 7; Figure S25. 13C NMR spectrum (126 MHz, CDCl3) of dimethyl ether of compound 7; Figure S26. HMBC spectrum (500 MHz, CDCl3) of dimethyl ether of compound 7; Figure S27. 1H NMR spectrum (700 MHz, CDCl3) of methyl ether of 4-ethyl-2-formyl-3,5,6-trihydroxybenzoic acid (8); Figure S28. 13C NMR spectrum (175 MHz, CDCl3) of methyl ether of 8; Figure S29. HMBC spectrum (700 MHz, CDCl3) of methyl ether of 8; Figures S30–S34. HMBC correlations of methyl ether of 8 (enlarged); Figure S35. 1H NMR spectrum (700 MHz, acetone-d6) of 4-ethyl-2,3,5-trihydroxybenzoic acid (9); Figure S36. 13C NMR spectrum (175 MHz, aceton-d6) of 9; Figure S37. HMBC spectrum (700 MHz, aceton-d6) of 9; Figures S38 and S39. HMBC correlations of 9 (enlarged); Figure S40. 1H NMR spectrum (700 MHz, CDCl3) of 3-ethyl-2,5-dihydroxy-1,4-benzoquinone (10); Figure S41. 13C NMR spectrum (175 MHz, CDCl3) of 10; Figure S42. HSQC spectrum (700 MHz, CDCl3) of 10; Figure S43. 1H NMR spectrum (700 MHz, CDCl3) of dimethyl ether of 10; Figure S44. 13C NMR spectrum (175 MHz, CDCl3) of dimethyl ether of 10; Figure S45. HMBC spectrum (700 MHz, CDCl3) of dimethyl ether of 10; Figures S46–S48. HMBC correlations of dimethyl ether of 10; Table S7. Histochrome toxicity values (intraperitoneal administration); Table S8. Accounting for chromosomal aberrations in mammalian bone marrow cells; Table S9. The results of a study of the mutagenic effect of the histochrome drug on indicator strains in the Ames test; Table S10. The results of the study of the ability of the histochrome drug to induce dominant lethal mutations in the germ cells of mice.
