# Supplementary material for: Isolation and Structure Determination of Echinochrome A Oxidative Degradation Products
Source: Molecules. 2020 Oct 18;25(20):4778. doi: 10.3390/molecules25204778 (PMC7587531; doi:10.3390/molecules25204778)
Supplement: Supplementary file 1 [file molecules-25-04778-s001.pdf]

molecules

MDPI

Supplementary Material

Isolation and Structure Determination of Echinochrome A Oxidative Degradation Products

Natalia P. Mishchenko [1,*], Elena A. Vasileva [1], Andrey V. Gerasimenko [2], Valeriya P. Grigorchuk [3], Pavel S. Dmitrenok [1] and Sergey A. Fedoreyev [1]

[1] G.B. Elyakov Pacific Institute of Bioorganic Chemistry, Far Eastern Branch of the Russian Academy of Sciences, Vladivostok 690022, Russia; vasilieva_el_an@mail.ru (E.A.V.); paveldmitrenok@mail.ru (P.S.D.); fedoreev-s@mail.ru (S.A.F.)

[2] Institute of Chemistry, Far Eastern Branch of the Russian Academy of Sciences, Vladivostok 690022, Russia; gerasimenko@ich.dvo.ru

[3] Federal Scientific Center of the East Asia Terrestrial Biodiversity, Far-Eastern Branch of Russian Academy of Sciences, Vladivostok 690022, Russia; kera1313@mail.ru

* Correspondence: mischenkonp@mail.ru, Tel.: +8(423) 231-40-50

TABLE OF CONTENTS:

Figure S1. The UV–Vis spectrum of Histochrome (blue) and Echinochrome A (black) in ethanol solution containing 1 mM HCl. 5

Figure S2. Change in the concentration of echinochrome A during the oxidation of a 1% histochrome solution (1 mL in 30 mL of water), at room temperature in the light, 5 days. 6

Table S1. Accuracy and reproducibility of the quantification of echinochrome A (**1**) using HPLC method. 6

Figure S3. HR-ESI-MS (negative mode) data for methyl ethers of Ech A oxidation products obtained by methylation with methyl iodide 7

Table S2. HR-ESI-MS (negative mode) data for methyl ethers of Ech A oxidation products obtained by methylation with methyl iodide 8

Table S3. ESI-MS (negative mode) data for methyl ethers of Ech A oxidation products obtained by methylation with diazomethane. 9

Figure S4. IR spectrum ($CDCl_3$) of compound **7** dimethyl ether (ν 1000–2000 cm^{-1}) 10

Figure S5. IR spectrum ($CDCl_3$) of compound **7** dimethyl ether (ν 2000–4000 cm^{-1}) 11

Figure S6. IR spectrum ($CDCl_3$) of compound **8** methyl ether (ν 1000–2000 cm^{-1}) 12

Figure S7. IR spectrum ($CDCl_3$) of compound **8** methyl ether (ν 2000–4000 cm^{-1}) 13

Figure S8. IR spectrum ($CDCl_3$) of compound **10** (ν 1000–2000 cm^{-1}) 14

Figure S9. IR spectrum ($CDCl_3$) of compound **10** (ν 2000–4000 cm^{-1}) 15

Figure S10. IR spectrum ($CDCl_3$) of compound **11** (ν 1000–2000 cm^{-1}) 16

Figure S11. IR spectrum ($CDCl_3$) of compound **11** (ν 2000–4000 cm^{-1}) 17

Figure S12. ^{1}H-NMR spectrum (300 MHz, acetone-d_6) of 7-ethyl-2,2,3,3,5,6,8-heptahydroxy-2,3-dihydro-1,4-naphthoquinone **(2)** 19

Figure S13. ^{13}C-NMR spectrum (75 MHz, acetone-d_6) of **2** 20

Figure S14. HMBC spectrum (300 MHz, acetone-d_6) of **2** 21

Figure S15. HMBC correlations of **2** (enlarged) 22

Figure S16. HMBC correlations of **2** (enlarged) 23

Figure S17. HMBC correlations of **2** (enlarged) 24

Figure S18. ^{1}H-NMR spectrum (300 MHz, $CDCl_3$) of echinolactone **(11)** 25

Figure S19. ^{13}C-NMR spectrum (75 MHz, CD_3CN) of echinolactone **(11)** 26

Figure S20. HMBC spectrum (300 MHz, CD_3CN) of echinolactone **(11)** 27

Table S4. Selected crystal data and refinement parameters for *α*- and β- forms of $C_{11}H_8O_7{\cdot}H_2O$ 28

Table S5. Selected geometric parameters (Å) for *α*- and β- forms of $C_{11}H_8O_7{\cdot}H_2O$ 29

Table S6. Hydrogen-bond geometry (Å, °) for *α*- and β- forms of $C_{11}H_8O_7{\cdot}H_2O$ 30

Figure S21. Overall packing for *α*-$C_{11}H_8O_7{\cdot}H_2O$ viewed along the *a*-axis direction (hydrogen bonds are shown as dashed lines) 31

Figure S22. A plot of band for β-$C_{11}H_8O_7{\cdot}H_2O$ (hydrogen bonds are shown as dashed lines) 32

Figure S23. Overall packing for β-$C_{11}H_8O_7{\cdot}H_2O$ viewed along the *a*-axis direction (hydrogen bonds are shown as dashed lines) 33

Figure S24. ^{1}H-NMR spectrum (500 MHz, $CDCl_3$) of dimethyl ether of compound **7** 35

Figure S25. ^{13}C-NMR spectrum (126 MHz, $CDCl_3$) of dimethyl ether of compound **7** 36

Figure S26. HMBC spectrum (500 MHz, $CDCl_3$) of dimethyl ether of compound **7** 37

Figure S27. ^{1}H-NMR spectrum (700 MHz, $CDCl_3$) of methyl ether of 4-ethyl-2-formyl-3,5,6-trihydroxybenzoic acid **(8)** 38

Figure S28. ^{13}C-NMR spectrum (175 MHz, $CDCl_3$) of methyl ether of **8**.. 39

Figure S29. HMBC spectrum (700 MHz, $CDCl_3$) of methyl ether of **8** 40

Figure S30. HMBC correlations of methyl ether of **8** (enlarged).. 41

Figure S31. HMBC correlations of methyl ether of **8** (enlarged).. 42

Figure S32. HMBC correlations of methyl ether of **8** (enlarged).. 43

Figure S33. HMBC correlations of methyl ether of **8** (enlarged).. 44

Figure S34. HMBC correlations of methyl ether of **8** (enlarged).. 45

Figure S35. ^{1}H-NMR spectrum (700 MHz, acetone-d_6) of 4-ethyl-2,3,5-trihydroxybenzoic acid **(9)** 46

Figure S36. ^{13}C-NMR spectrum (175 MHz, acetone-d_6) of **9**.. 47

Figure S37. HMBC spectrum (700 MHz, acetone-d_6) of **9**.. 48

Figure S38. HMBC correlations of **9** (enlarged).. 49

Figure S39. HMBC correlations of **9** (enlarged).. 50

Figure S40. ^{1}H-NMR spectrum (700 MHz, $CDCl_3$) of 3-ethyl-2,5-dihydroxy-1,4-benzoquinone **(10)**........................ 51

Figure S41. ^{13}C-NMR spectrum (175 MHz, $CDCl_3$) of **10** 52

Figure S42. HSQC spectrum (700 MHz, $CDCl_3$) of **10** 53

Figure S43. ^{1}H-NMR spectrum (700 MHz, $CDCl_3$) of dimethyl ether of **10**.. 54

Figure S44. ^{13}C-NMR spectrum (175 MHz, $CDCl_3$) of dimethyl ether of **10**.. 55

Figure S45. HMBC spectrum (700 MHz, $CDCl_3$) of dimethyl ether of **10**.. 56

Figure S46. HMBC correlations of dimethyl ether of **10** 57

Figure S47. HMBC correlations of dimethyl ether of **10** 58

Figure S48. HMBC correlations of dimethyl ether of **10** 59

Table S7. Histochrome toxicity values (intraperitoneal administration).. 60

Table S8. Accounting for chromosomal aberrations in mammalian bone marrow cells. 61

Table S9. The results of a study of the mutagenic effect of the histochrome drug on indicator strains in the Ames test. Histochrome in concentrations of 0.1–1000 μg/plate does not exhibit a mutagenic effect in the Ames test. 62

Table S10. The results of the study of the ability of the histochrome drug to induce dominant lethal mutations in the germ cells of mice. Histochrome does not induce dominant lethal mutations in mature sperm or late and early mouse spermatids.. 62

Echinochrome A and Histochrome

Drug substance echinochrome A and drug product Histochrome were produced by G.B. Elyakov Pacific Institute of Bioorganic Chemistry (Vladivostok, Russia).

Echinochrome A (Reg. No. P N002362/02) is a dark-red crystalline powder, melting point (m.p.) 219–221.5 °C, moderately (slowly) soluble in 95% alcohol, very slightly soluble in chloroform, and practically insoluble in water. The UV–Vis spectrum of a 0.002% solution of echinochrome A in EtOH·1mM HCl has λ_{max} at 342 ± 2 and 468 ± 2 nm, λ_{min} at 295 ± 2 and 392 ± 2 nm, and two shoulders from 485 to 500 nm and from 515 to 537 nm. The specific absorption rate of echinochrome $A_{1\,cm}^{1\%}$ at λ_{468} is 270–274.

The substance echinochrome A contains no more than 2% of natural impurities, involving other spinochromes of the sea urchin [Mishchenko N. P., Fedoreev S. A., Glazunov V. P., Denisenko V. A., Krasovskaya N. P., Glebko L. I., Maslov L. G., Dmitrenok P. S., Bagirova V. L. Isolation and identification of impurities in the parent substance of echinochrome and in the drug histochrome. Pharmaceutical Chemistry Journal Vol. 38, No. 1, 2004, 50-53].

Histochrome solution for intravenous administration, 10 mg/mL (Reg. No. P N002362/01) is a dark-brown solution, pH 7.0–8.0, containing a sodium derivative of echinochrome A obtained in inert conditions; there are no other components.

Histochrome for injection, 0.2 mg/mL (Reg. No. P N002362/02) is a transparent solution of red-brown color, pH 6.5–7.5, containing the sodium derivative of echinochrome A and 0.9% solution sodium chloride; there are no other components.

The UV–Vis spectrum of Histochrome (10 mg/mL diluted 400 times) and Histochrome (0.2 mg/mL diluted 12.5 times) in ethanol containing 1 mM HCl should be identical to the UV–Vis spectrum of the echinochrome A substance solution (Figure S1).

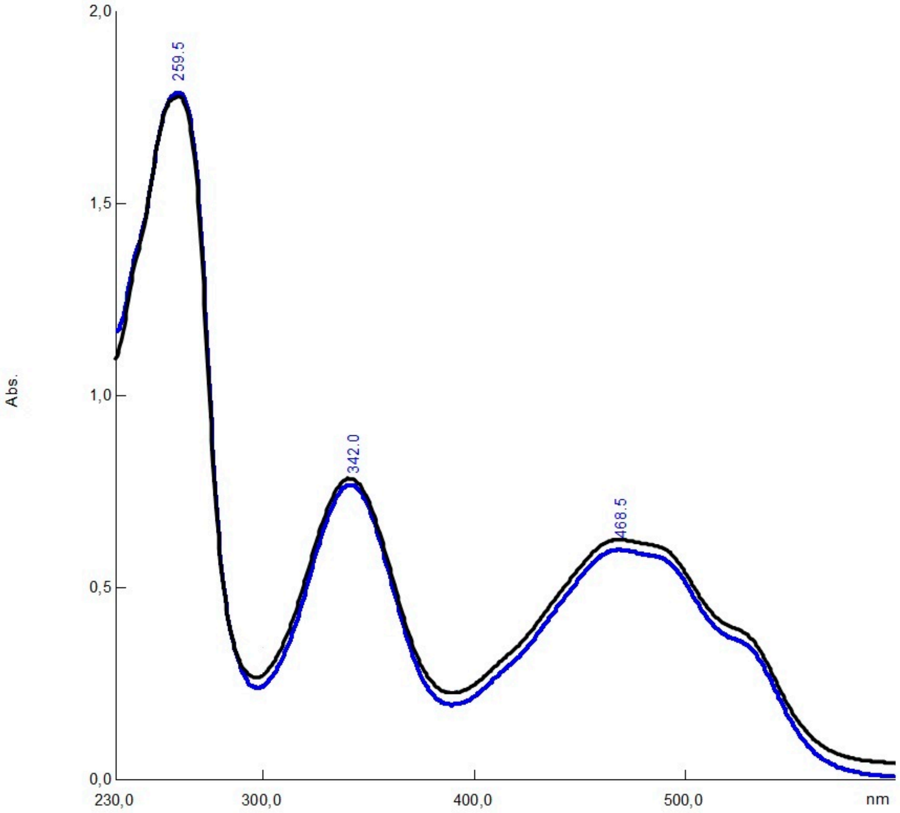

Figure S1. The ultraviolet–visible light (UV–Vis) spectrum of Histochrome (blue) and echinochrome A (black) in ethanol solution containing 1 mM HCl.

The concentration of echinochrome A in histochrome can be determined spectrophotometrically in acidified ethanol at a wavelength of 468 nm according to the following formula:

$$C = \frac{A}{A_{1\,cm}^{1\%} \times b},$$

where C is the echinochrome A concentration in g/100 mL, A is the optical density of the Histochrome solution, $A_{1\,cm}^{1\%}$ is the specific absorption rate of echinochrome A, and b is the optical path length or layer thickness, in cm.

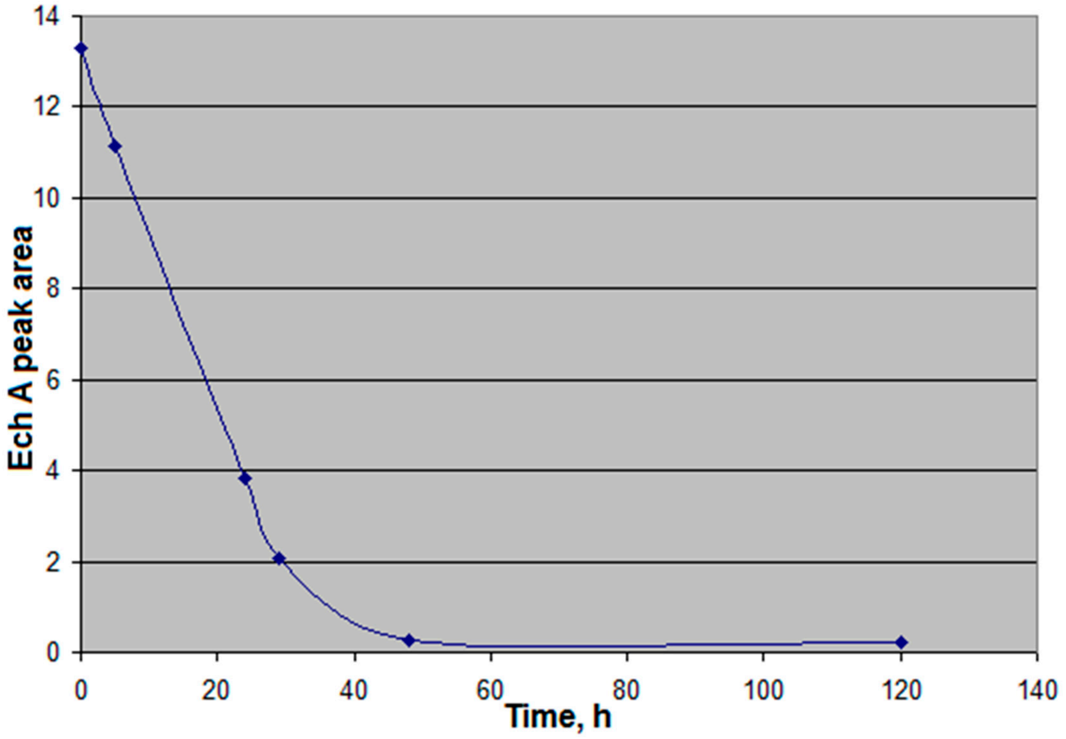

Figure S2. Change in the concentration of echinochrome A during the oxidation of a 1% Histochrome solution (1 mL in 30 mL of water), at room temperature in the light, 5 days.

Table S1. Accuracy and reproducibility of the quantification of echinochrome A (**1**) using HPLC method.

**Echinochrome A (1)**	**Level 1 (100 ng/mL)**	**Accuracy, %**	**Level 2 (250 ng/mL)**	**Accuracy, %**	**Level 3 (500 ng/mL)**	**Accuracy, %**
Sample 1	105	105.0	238	95.2	512	102.4
Sample 2	100	100.0	248	99.2	518	103.6
Sample 3	98	98.0	253	101.2	503	100.6
Sample 4	103	103.0	262	104.8	509	101.8
$\bar{X}$ *	101.5		250.3		510.5	
S **	3.1		10.2		6.2	
S_r ***, %	3.06		4.0		1.2	

* $\bar{X}$, mean; ** S, standard deviation; *** S_r, relative standard deviation, %

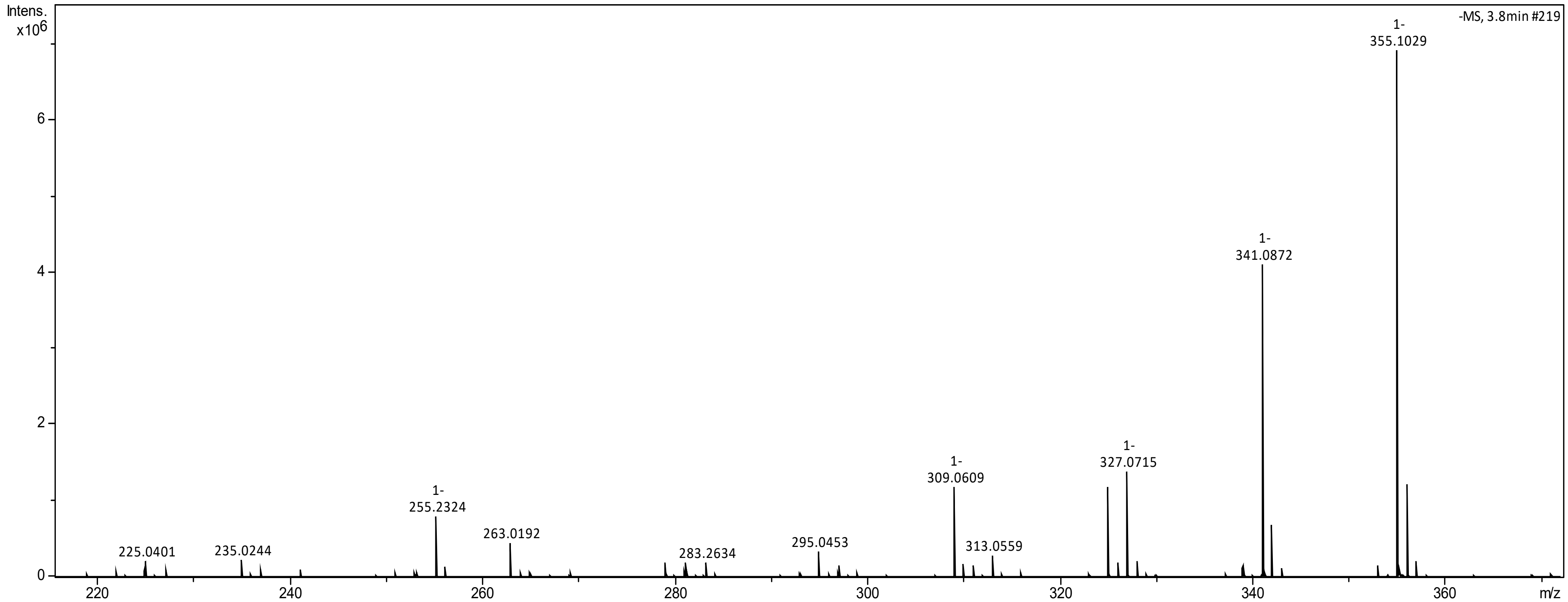

Figure S3. HR-ESI-MS (negative mode) data for methyl ethers of Ech A oxidation products obtained by methylation with methyl iodide.

Table S2. HR-ESI-MS (negative mode) data for methyl ethers of Ech A oxidation products obtained by methylation with methyl iodide.

Structure	Formula	Molecular mass	$[M-H]^-$ calculated	$[M-H]^-$ measured
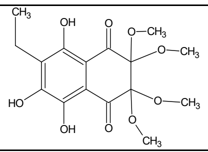	$C_{16}H_{20}O_9$	356	355.1035	355.1029
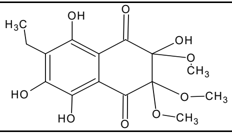	$C_{15}H_{18}O_9$	342	341.0878	341.0872
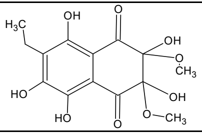	$C_{14}H_{16}O_9$	328	327.0722	327.0715
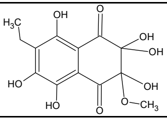	$C_{13}H_{14}O_9$	314	313.0565	313.0559
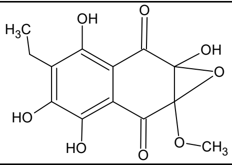	$C_{13}H_{12}O_8$	296	295.0459	295.0453
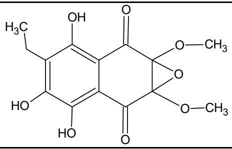	$C_{14}H_{14}O_8$	310	309.0616	309.0609

Table S3. ESI-MS (negative mode) data for methyl ethers of Ech A oxidation products obtained by methylation with diazomethane.

Structure	Formula	Molecular mass	[M − H]⁻ measured
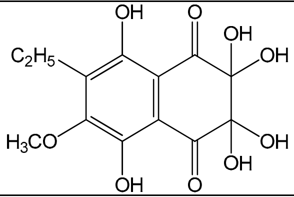	$C_{13}H_{14}O_9$	314	313
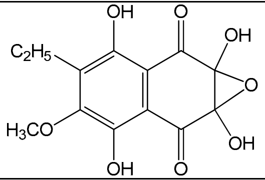	$C_{13}H_{12}O_8$	296	295
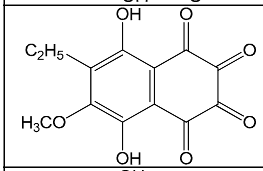	$C_{13}H_{10}O_7$	278	277
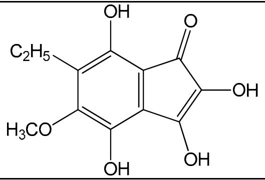	$C_{12}H_{12}O_6$	252	251

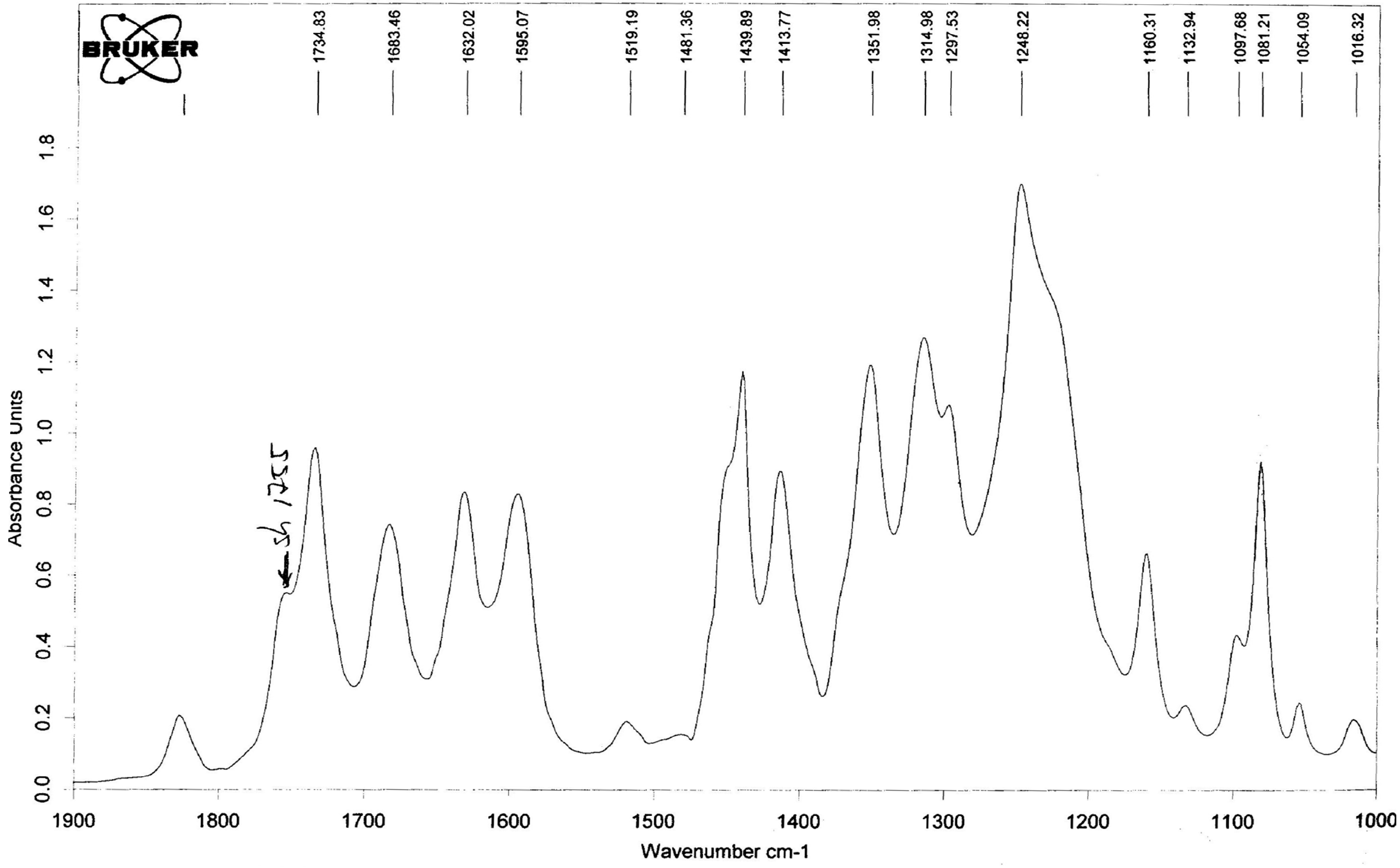

Figure S4. Infrared (IR) spectrum ($CDCl_3$) of compound **7** dimethyl ether (ν 1000–2000 cm^{-1})

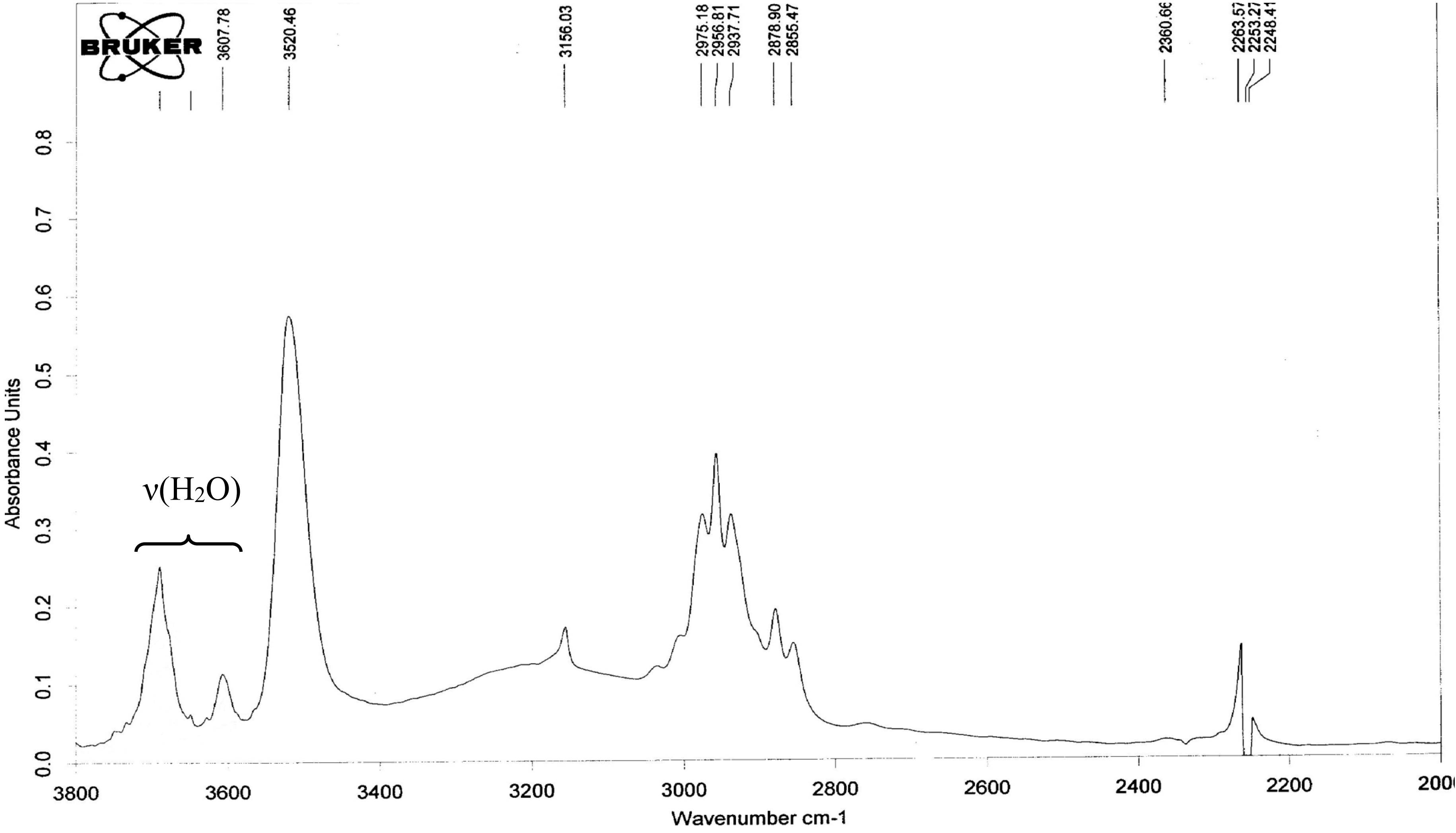

Figure S5. IR spectrum ($CDCl_3$) of compound **7** dimethyl ether (ν 2000–4000 cm^{-1})

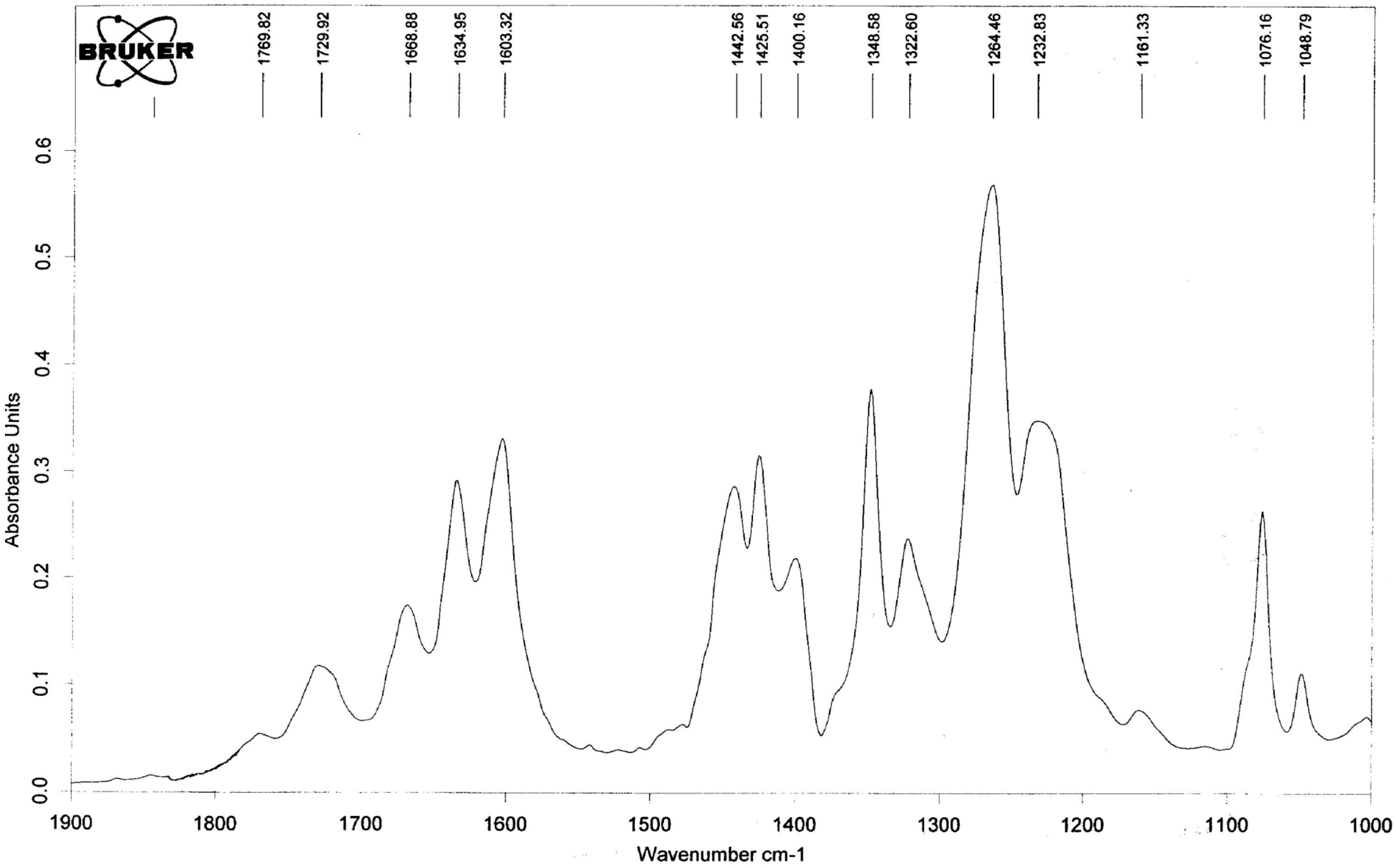

Figure S6. IR spectrum ($CDCl_3$) of compound **8** methyl ether (ν 1000–2000 cm^{-1})

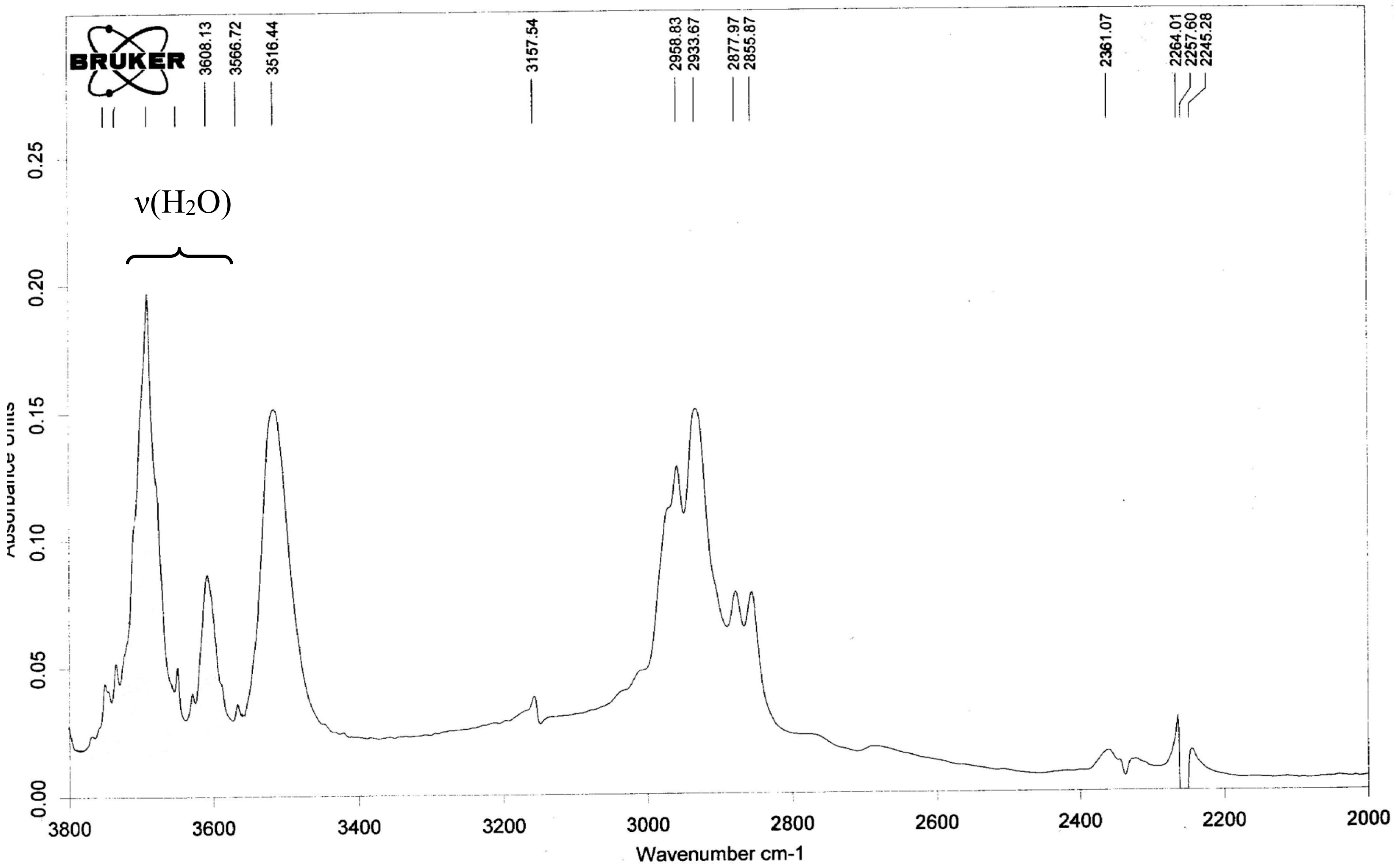

Figure S7. IR spectrum ($CDCl_3$) of compound **8** methyl ether (ν 2000–4000 cm^{-1})

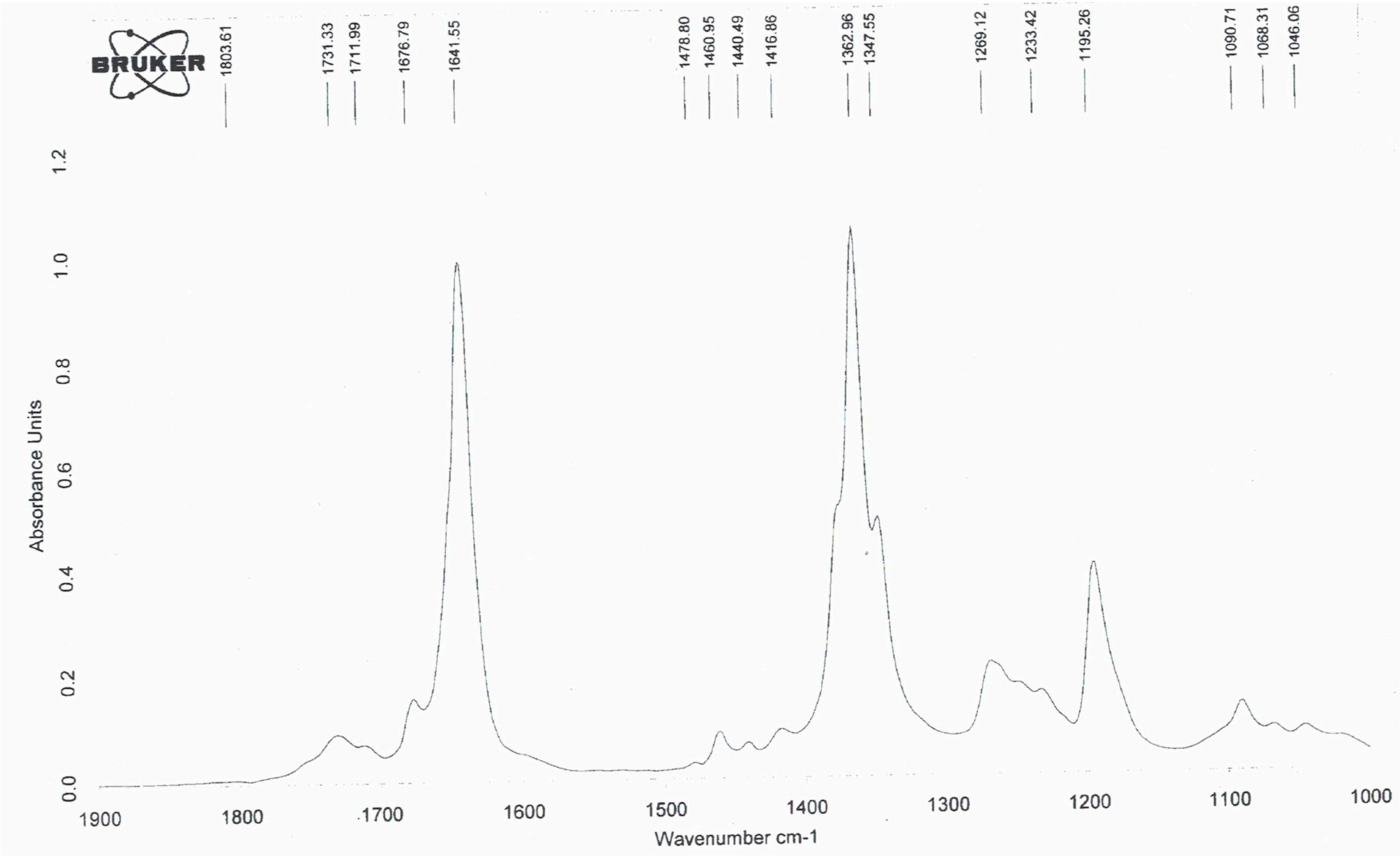

Figure S8. IR spectrum ($CDCl_3$) of compound **10** (ν 1000–2000 cm^{-1})

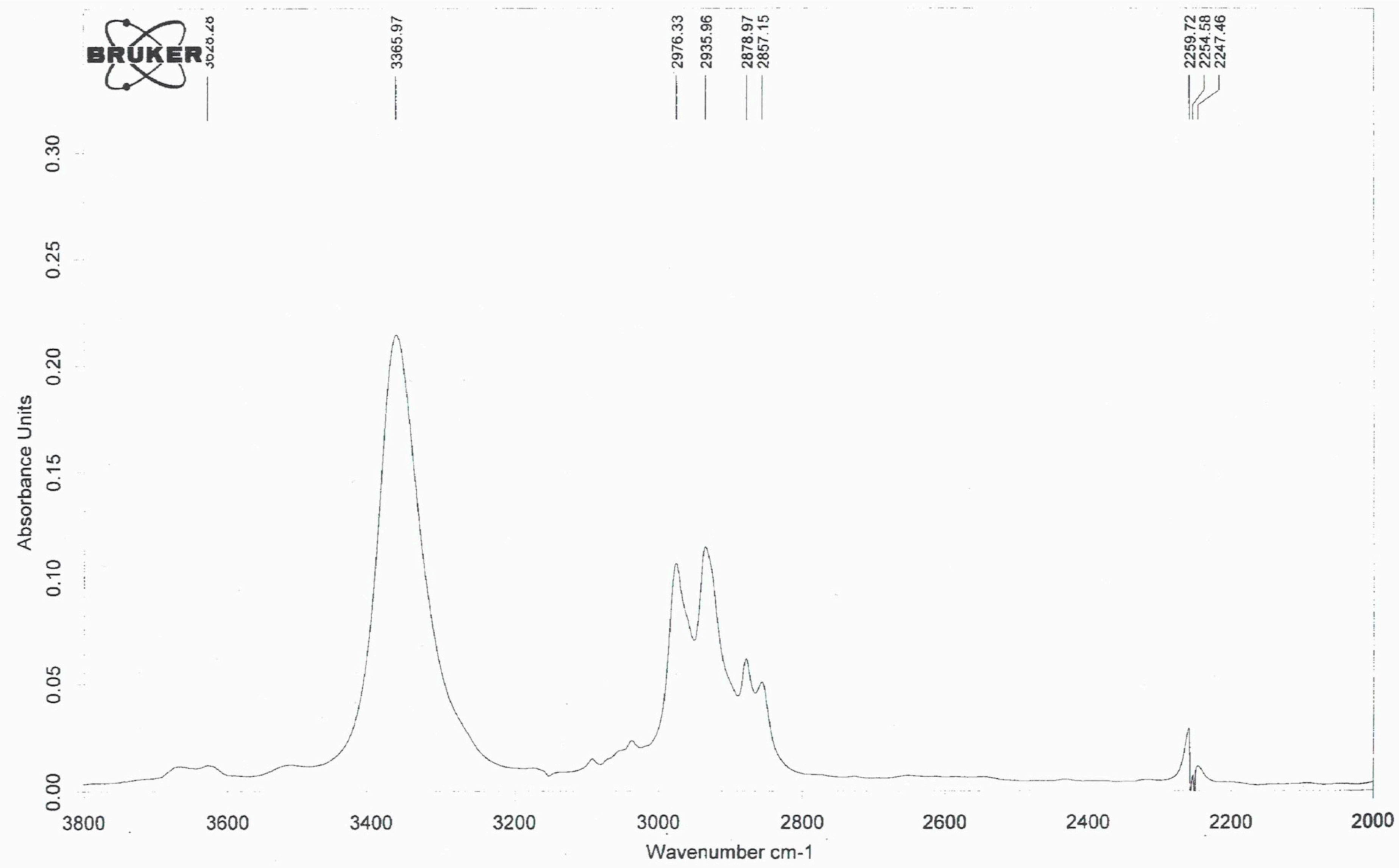

Figure S9. IR spectrum ($CDCl_3$) of compound **10** (ν 2000–4000 cm^{-1})

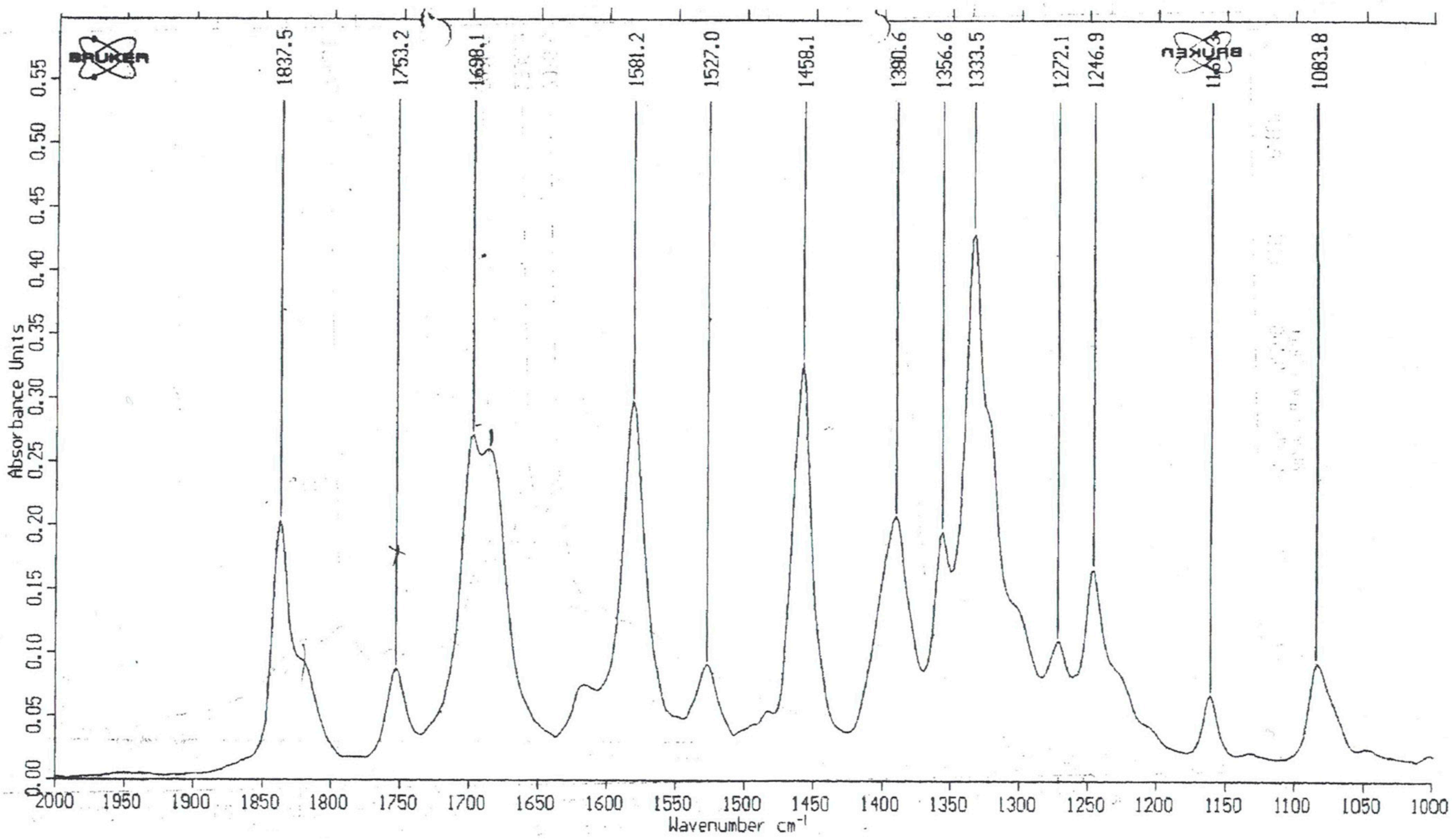

Figure S10. IR spectrum ($CDCl_3$) of compound **11** (ν 1000–2000 cm^{-1})

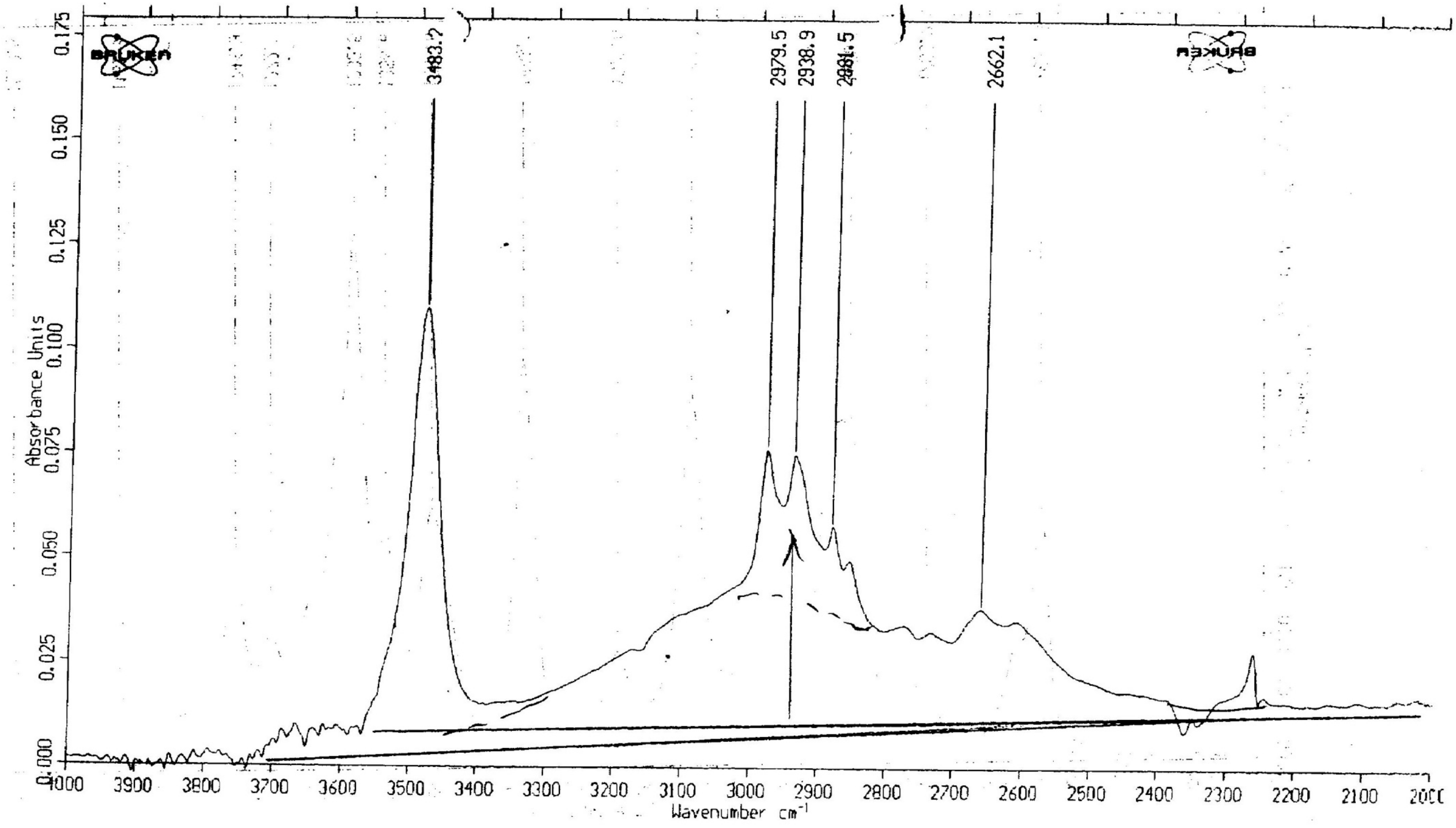

Figure S11. IR spectrum ($CDCl_3$) of compound **11** (ν 2000–4000 cm⁻¹)

7-Ethyl-2,2,3,3,5,6,8-heptahydroxy-2,3-dihydro-1,4-naphthoquinone (**2**)

IR spectrum, ν_{max}, cm^{-1}: 3520 (ν(2 C(2)–OH, 2 C(3)–OH, C(6)–OH)), 3160 (ν(C(5)–OH, C(8)–OH)), 2975 ($\nu_{as}(CH_2)$), 2939($\nu_{as}(CH_3)$), 2879(ν_s(CH3)), 1662 (ν(C=C), ν(C(4)=O)), 1652 (ν(C(1)=O), ν(C=C)), 1606 (ν(C=C), δ(*α*-OH)), 1589 (δ(*α*-OH, ν(C=C)),1457, 1404, 1352, 1300, 1191, 1157, 1124, 1066, 1050.

^{1}H-NMR spectrum ($CDCl_3$), δ: 1.18 (t, 3 H, Me, *J* = 7.5 Hz); 2.78 (q, 2 H, CH2, *J* = 7.5 Hz); 4.44 (br.s, 2 H, C(2)OH, C(3)OH); 4.51 (br.s, 2 H, C(2)OH, C(3) OH); 6.75 (br.s, 1 H, C(6)OH); 11.07 (s, 1 H, C(5) OH); 11.58 (s, 1 H, C(8)OH).

^{1}H-NMR spectrum (acetone-d_6), δ: 1.16 (t, 3 H, Me, *J* = 7.5 Hz); 2.78 (q, 2 H, CH_2, *J* = 7.5 Hz); 5.91, 5.96 (both br.s, 2 H each, C(2)OH, C(3)OH); 9.50 (br.s, 1 H, C(6)OH); 11.28 (br.s, 1 H, C(5)OH); 11.88 (s, 1 H, C(8)OH).

^{13}C-NMR spectrum (acetone-d_6), δ: 12.69 (Me); 17.02 (CH_2); 94.59 (C(2)); 94.70 (C(3)); 105.45 (C(8a)); 111.38 (C(4a)); 127.01 (C(7)); 145.40 (C(5)); 152.77 (C(6)); 157.59 (C(8)); 197.00 (C(1)); 198.88 (C(4)).

The ^{1}H-NMR spectrum of **2** recorded in $CDCl_3$ showed signals for the protons of OH groups at positions 2 and 3 as two broadened singlets with 2 H integral intensity each at δ 4.44 and 4.51.

In acetone-d_6, this chemical shift difference decreased (δ 5.91, br.s, 2 H, and δ 5.96, br.s, 2 H); in DMSO-d_6, these signals coincided (δ 6.75, br.s, 4 H).

The physical and spectroscopic data agree with those reported in the literature [V.L. Novikov, O.P. Shestak, N.P. Mishchenko, et al., Oxidation of 7-ethyl-2,3,5,6,8-pentahydroxy-1,4naphthoquinone (echinochrome A) by atmospheric oxygen. 1. Structure of dehydroechinochrome, Russ. Chem. Bull. 67 (2018) 282–290. DOI: 10.1007/s11172-018-2071-1].

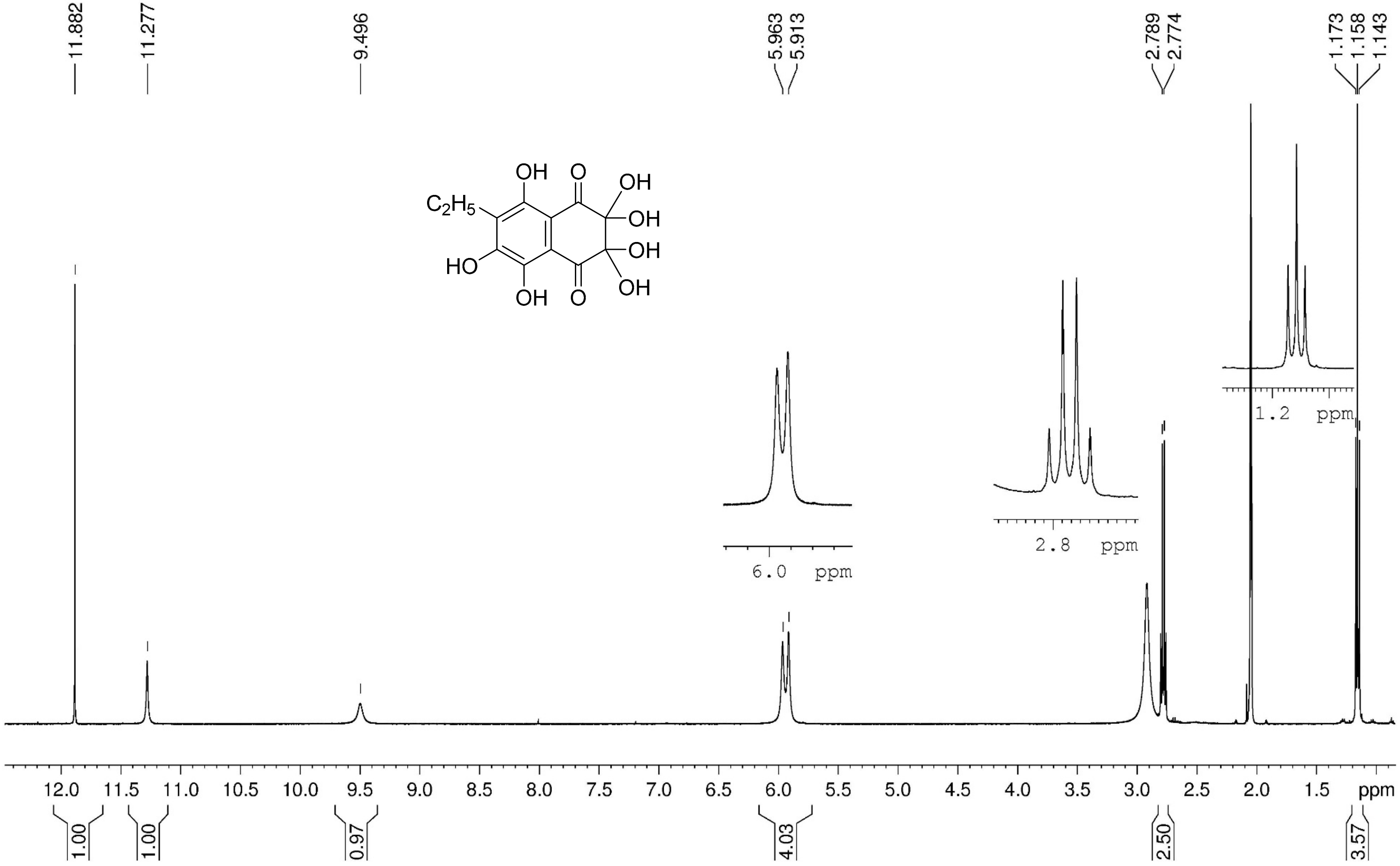

Figure S12. ^{1}H-NMR spectrum (300 MHz, acetone-d_6) of 7-ethyl-2,2,3,3,5,6,8-heptahydroxy-2,3-dihydro-1,4-naphthoquinone **(2).**

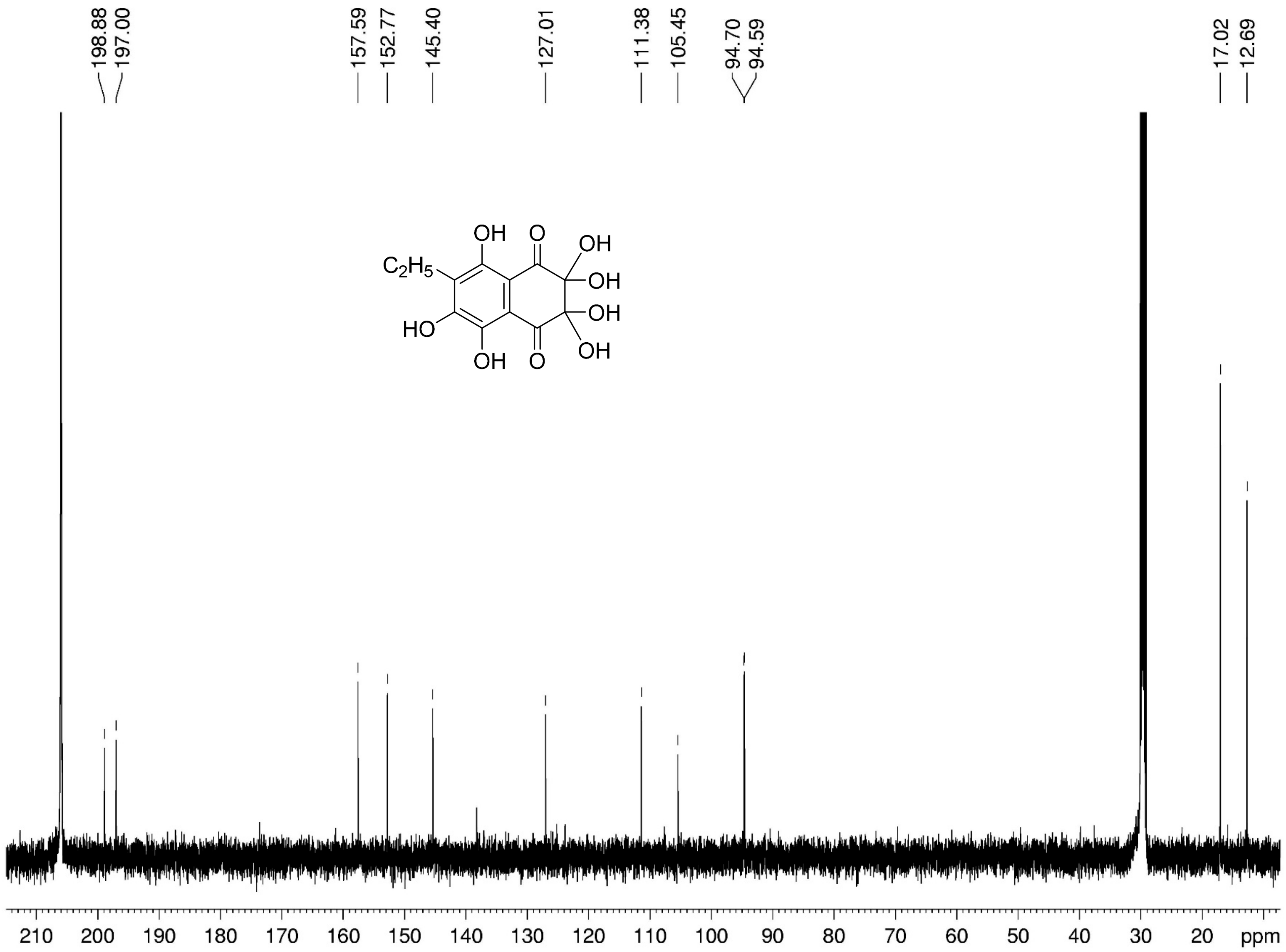

Figure S13. ^{13}C-NMR spectrum (75 MHz, acetone-d6) of **2.**

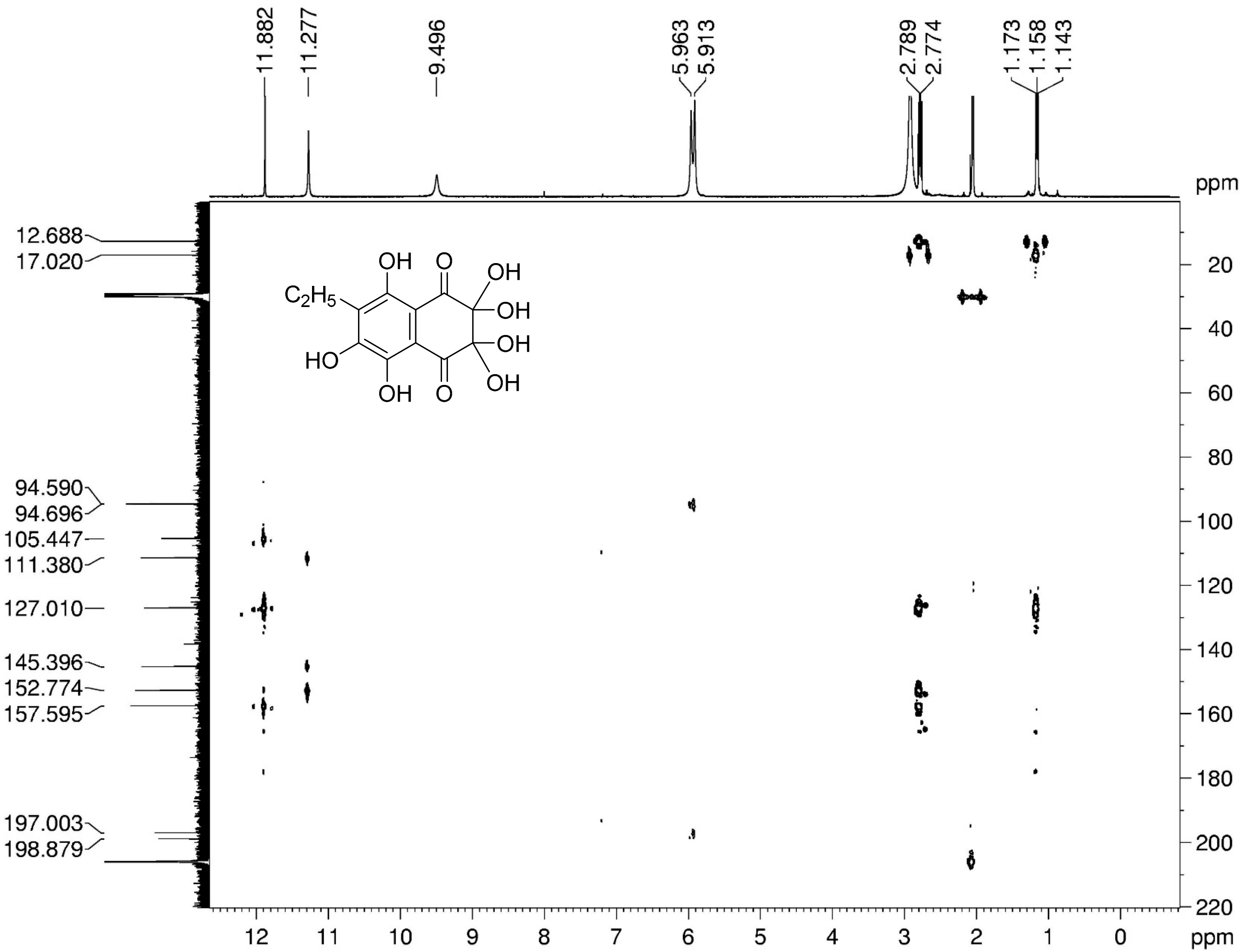

Figure S14. HMBC spectrum (300 MHz, acetone-d6) of **2.**

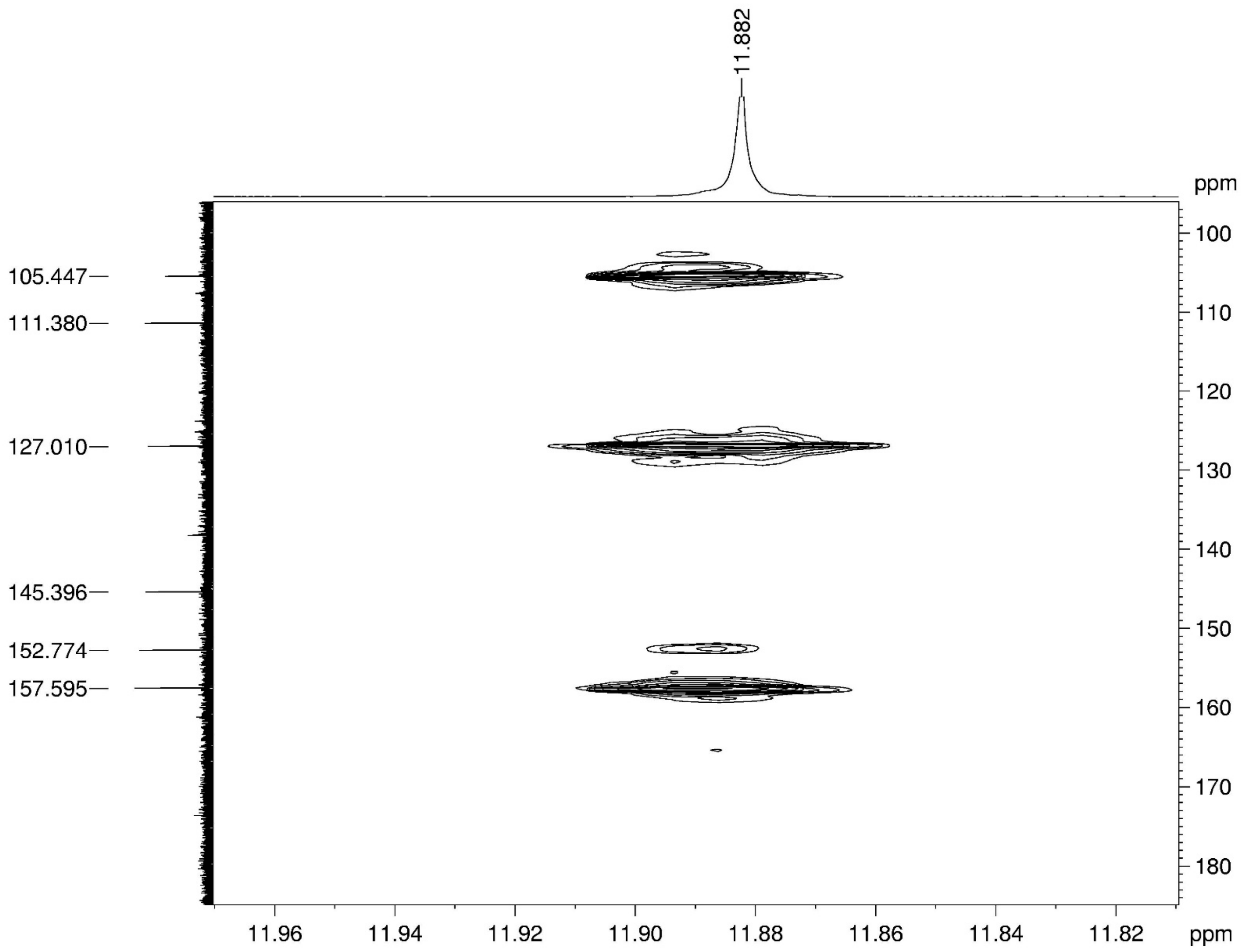

Figure S15. HMBC correlations of **2** (enlarged).

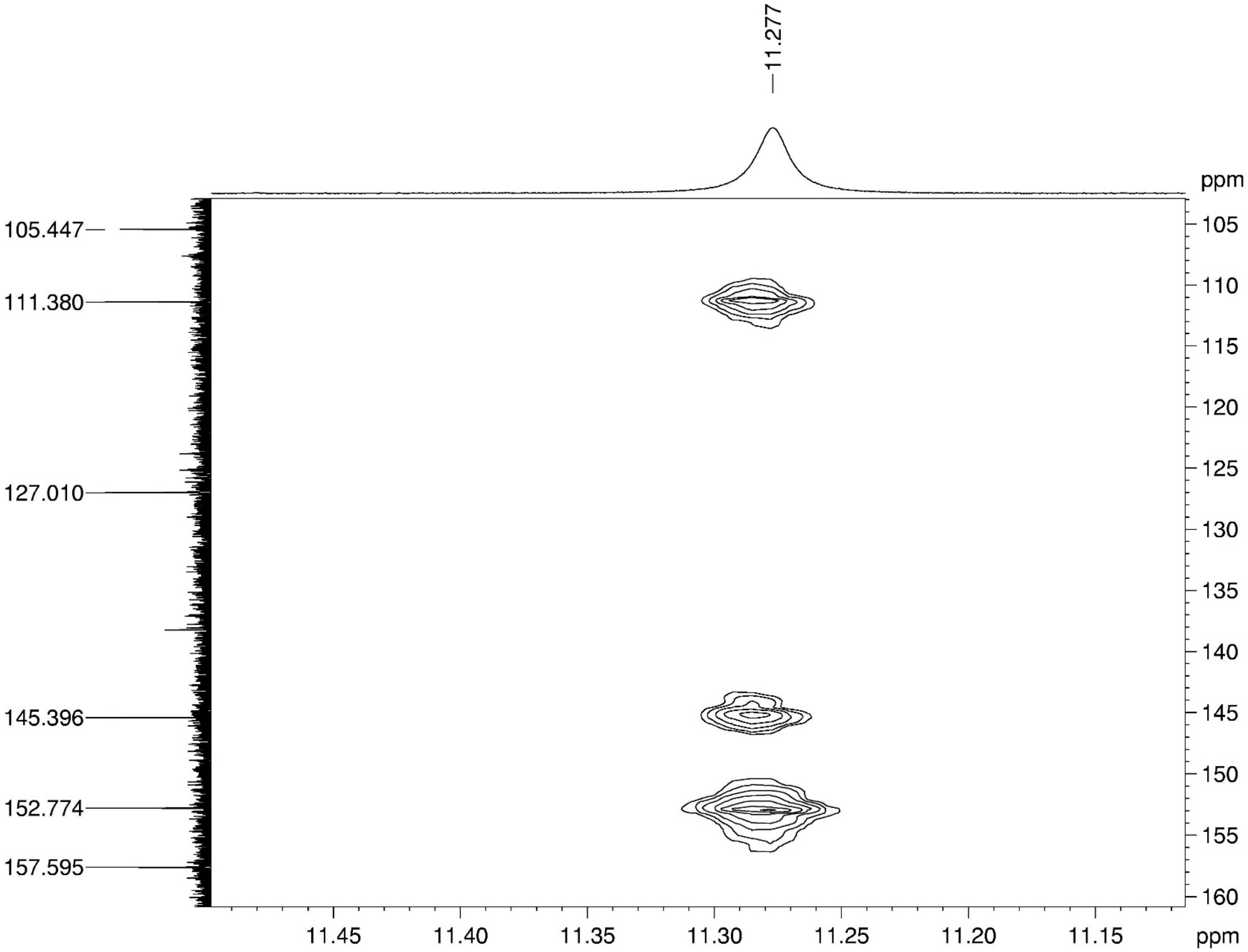

Figure S16. HMBC correlations of **2** (enlarged).

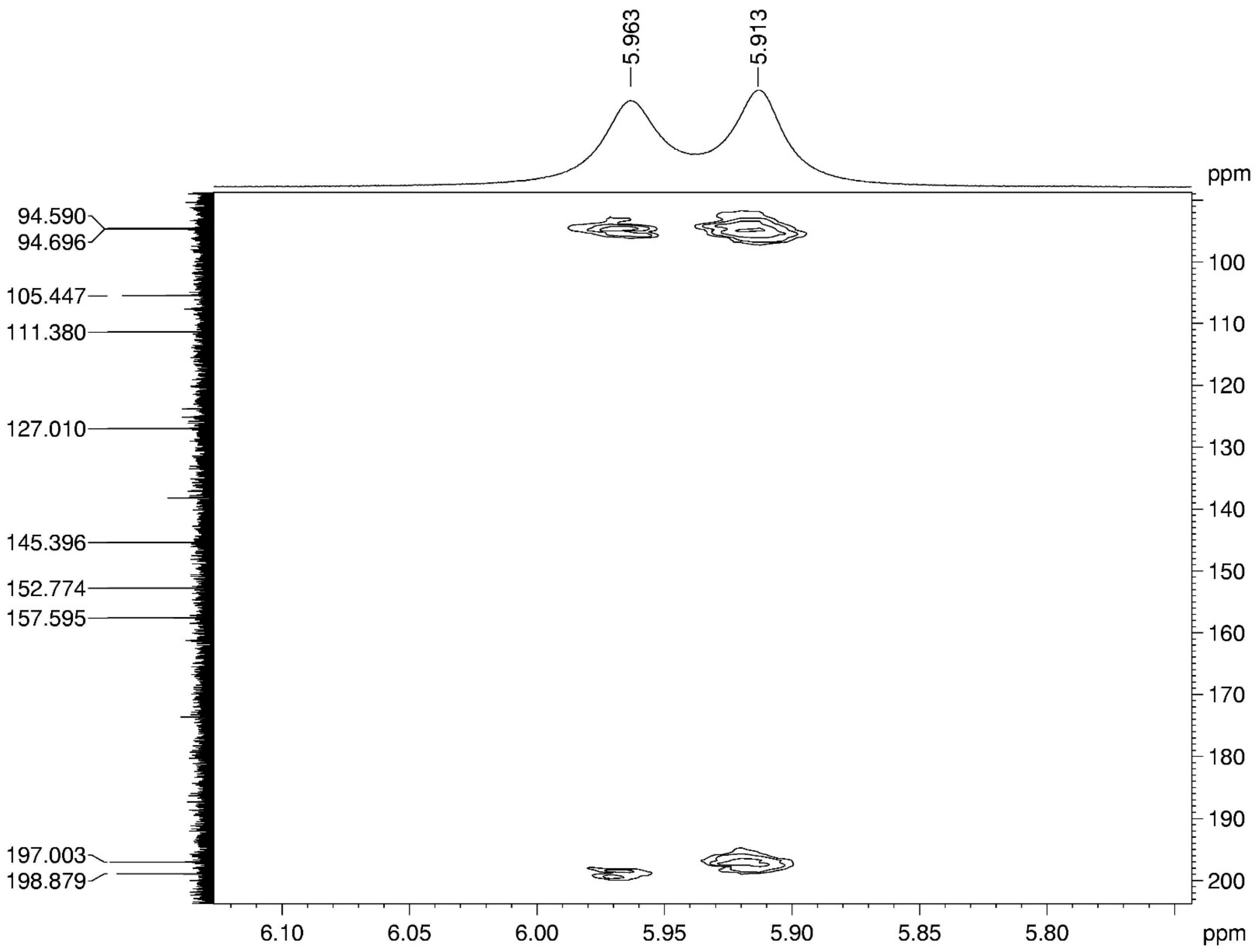

Figure S17. HMBC correlations of **2** (enlarged).

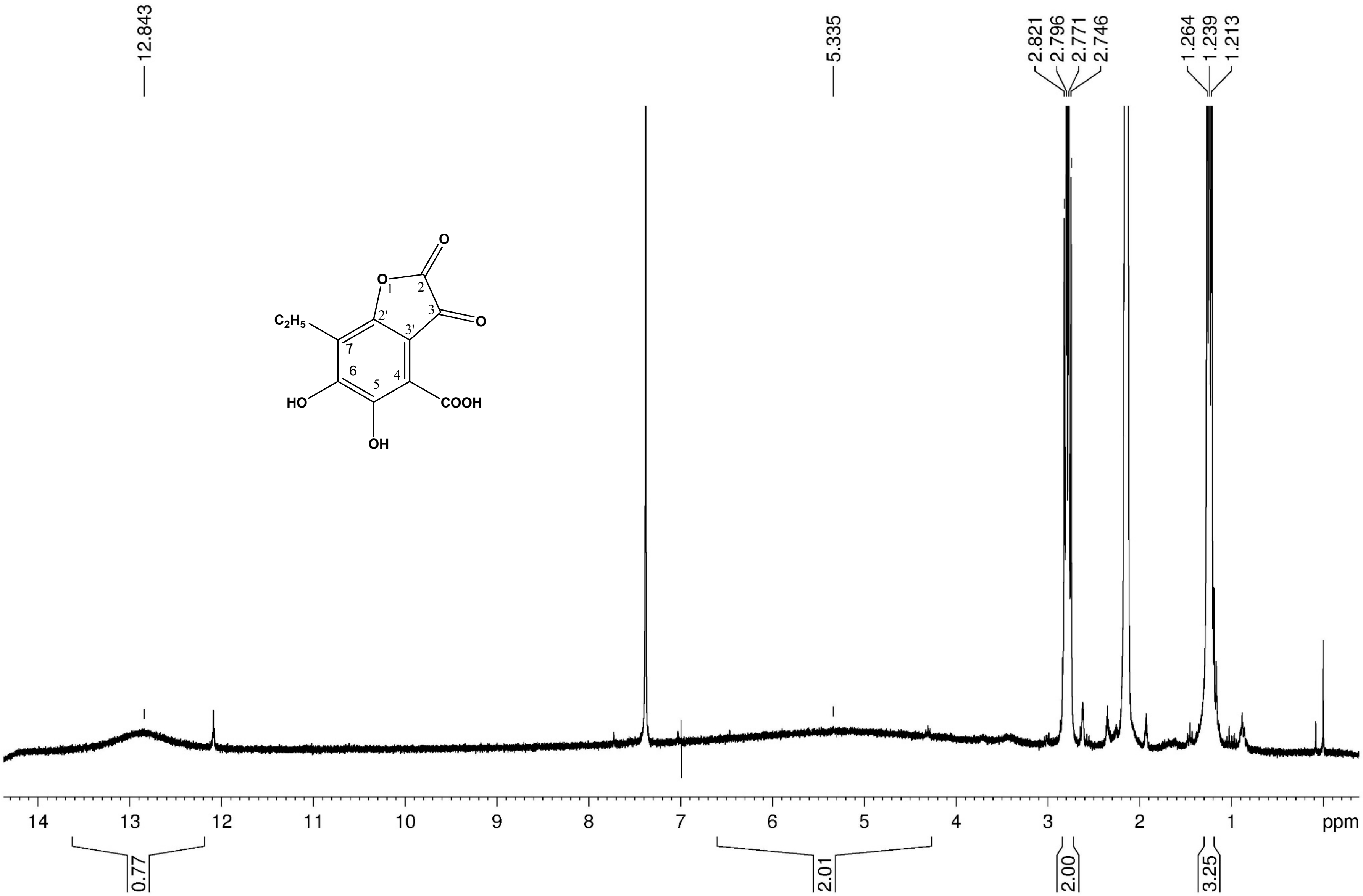

Figure S18. ^{1}H-NMR spectrum (300 MHz, $CDCl_3$) of echinolactone **(11).**

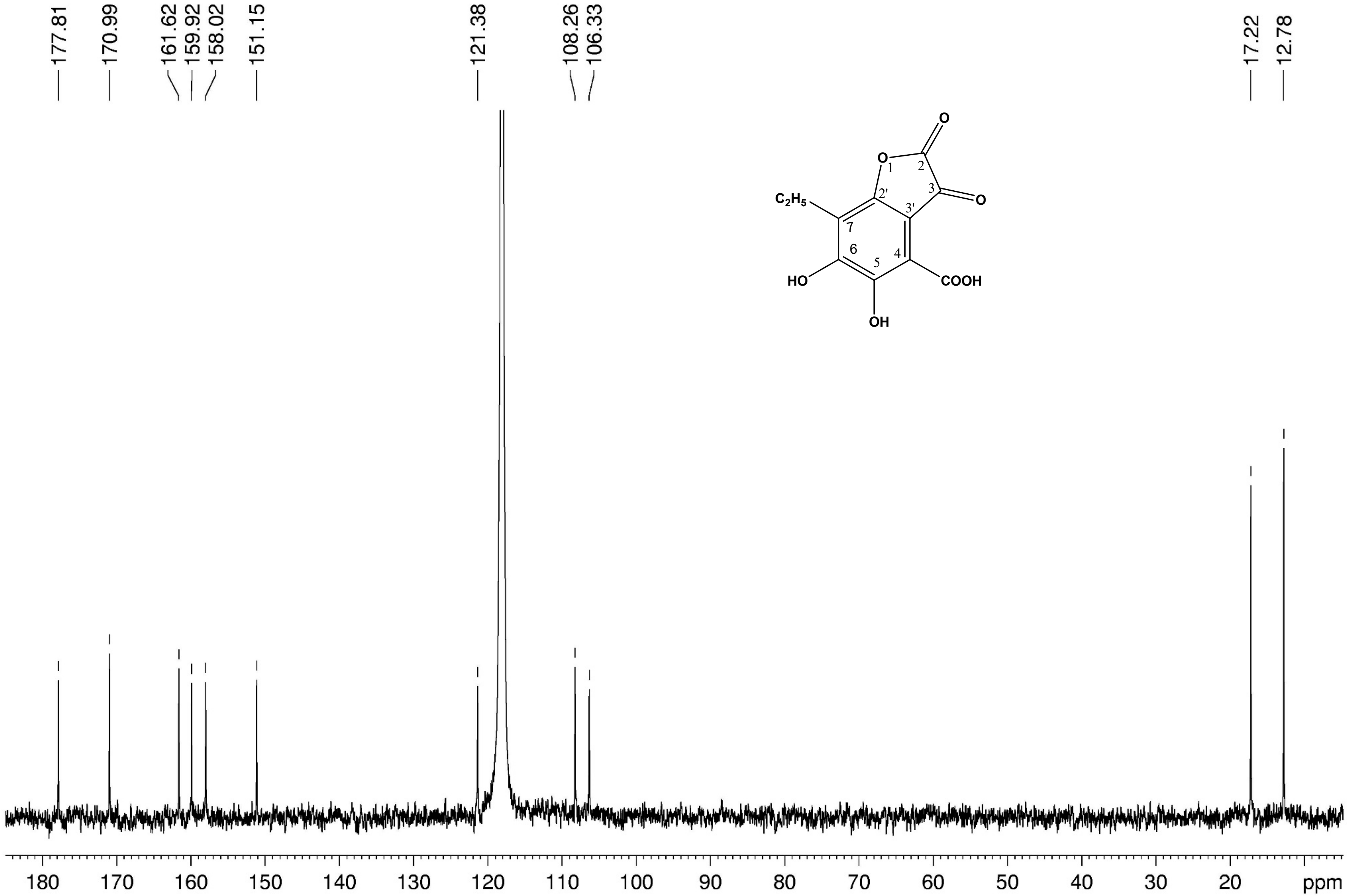

Figure S19. ^{13}C-NMR spectrum (75 MHz, CD_3CN) of echinolactone **(11).**

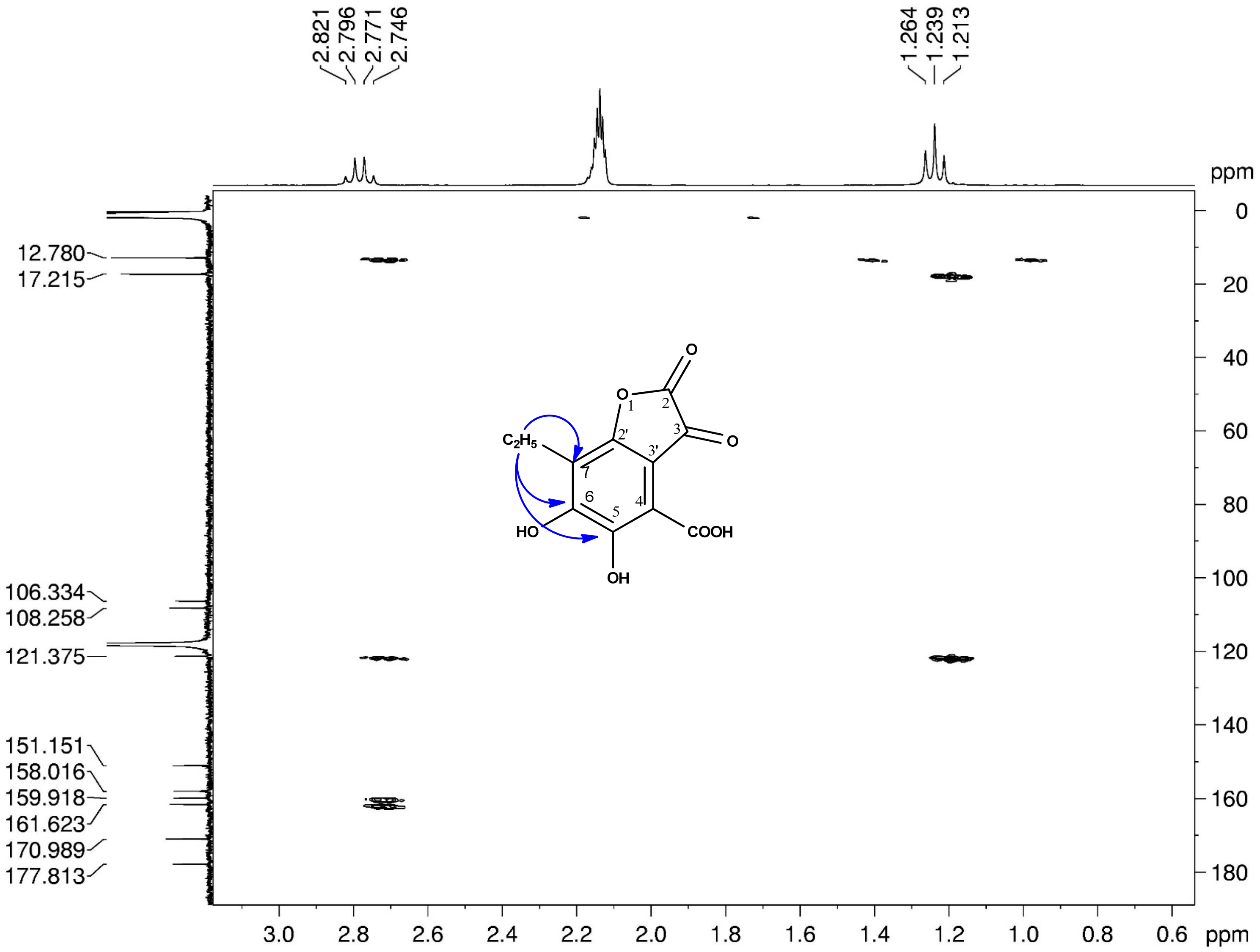

Figure S20. HMBC spectrum (300 MHz, CD_3CN) of echinolactone **(11).**

Table S4. Selected crystal data and refinement parameters for α- and β- forms of $C_{11}H_8O_7 \cdot H_2O$

Compound	α-$C_{11}H_8O_7 \cdot H_2O$	β-$C_{11}H_8O_7 \cdot H_2O$
Formula weight	270.19	270.19
Temperature (K)	173(2)	173(2)
Radiation type	Mo *Kα*	Mo *Kα*
Space group	$P2_1/c$	$P\bar{1}$
Color/specimen shape (size (mm))	Dark red/plate (0.38 × 0.09 × 0.02)	Orange/prism (0.18 × 0.13 × 0.08)
Unit cell dimentions (Å, °)	*a* =15.650(4), *b* =13.482(3), *c* =5.1857(12), β = 92.781(6)	*a* =4.7823(6), *b* =7.9520(9), *c* =14.4705(17), *α* =86.271(3), β =87.082(2), γ =79.301(3)
V ($Å^3$)/Z	1092.9(5)/4	539.16(11)/2
D_{calc} (g/cm^3)	1.642	1.664
θ range (°)	3.992–28.049	3.954–28.012
Range of *h*, *k* and *l*	−19/20, −17/17, −5/6	−5/6, −10/8, −14/19
F(000)	560	280
Reflections (measured/unique/with $I > 2\sigma(I)$)	6555/2598/1689 R_{int} = 0.0426	3324/2490/1595 R_{int} = 0.0466
Number of parameters	188	188
GooF	1.000	0.890
R_1, wR_2 ($F^2 > 2\sigma(F^2)$)	0.0512, 0.1271	0.0406, 0.0930
R_1, wR_2 (all data)	0.0886, 0.1467,	0.0693, 0.1012
$\Delta\varrho_{min}$, $\Delta\varrho_{max}$ (e/$Å^3$)	−0.292, 0.356	−0.260, 0.315

Table S5. Selected geometric parameters (Å) for α- and β- forms of $C_{11}H_8O_7 \cdot H_2O$.

α-$C_{11}H_8O_7 \cdot H_2O$					
O1–C2	1.388(3)	O7–C10	1.309(3)	C5–C6	1.432(3)
O1–C8	1.396(2)	C2–C3	1.533(3)	C6–C7	1.397(3)
O2–C2	1.179(3)	C3–C9	1.419(3)	C7–C8	1.382(3)
O3–C3	1.232(3)	C4–C5	1.386(3)	C7–C11	1.507(3)
O4–C5	1.342(2)	C4–C9	1.421(3)	C8–C9	1.391(3)
O5–C6	1.336(3)	C4–C10	1.480(3)	C11–C12	1.525(3)
O6–C10	1.232(3)				
β-$C_{11}H_8O_7 \cdot H_2O$					
O1–C2	1.370(2)	O7–C10	1.305(2)	C5–C6	1.423(3)
O1–C8	1.400(2)	C2–C3	1.542(3)	C6–C7	1.398(3)
O2–C2	1.188(2)	C3–C9	1.447(2)	C7–C8	1.383(2)
O3–C3	1.219(2)	C4–C5	1.388(2)	C7–C11	1.505(2)
O4–C5	1.349(2)	C4–C9	1.430(2)	C8–C9	1.398(2)
O5–C6	1.330(2)	C4–C10	1.470(3)	C11–C12	1.524(3)
O6–C10	1.232(2)				

Table S6. Hydrogen-bond geometry (Å, °) for α- and β- forms of $C_{11}H_8O_7{\cdot}H_2O$.

Bond O–H···O	Distance, Å			Angle, ° O-H···O
	O–H	H···O	O···O	
α-$C_{11}H_8O_7{\cdot}H_2O$ *				
O4-H4···O6	0.87(3)	1.79(3)	2.548(2)	145(3)
O5-H5···O8^{i}	0.91(3)	1.82(3)	2.704(2)	162(3)
O7-H7···O3	0.99(3)	1.64(3)	2.612(2)	166(3)
O8-H8A···O6	0.81(3)	2.14(3)	2.941(2)	172(3)
O8-H8B···O8^{ii}	0.91(3)	2.05(3)	2.9518(17)	169(3)
β-$C_{11}H_8O_7{\cdot}H_2O$ **				
O4-H4···O6	0.86(2)	1.77(2)	2.5605(19)	152(2)
O5-H5···O8^{ii}	0.81(2)	1.82(2)	2.6003(19)	159(2)
O7-H7···O3^{i}	0.90(3)	1.74(3)	2.6362(19)	172(2)
O8-H8A···O6	0.93(3)	2.01(3)	2.929(2)	172(2)
O8-H8B···O2^{i}	0.77(3)	2.27(3)	2.9929(19)	158(2)

Symmetry codes:

* (^{i}) $-x + 1, -y + 2, -z + 2$; (^{ii}) $x, -y + 3/2, z - 1$.

** (^{i}) $-x - 1, -y + 1, -z + 1$; (^{ii}) $-x + 1, -y, -z + 1$.

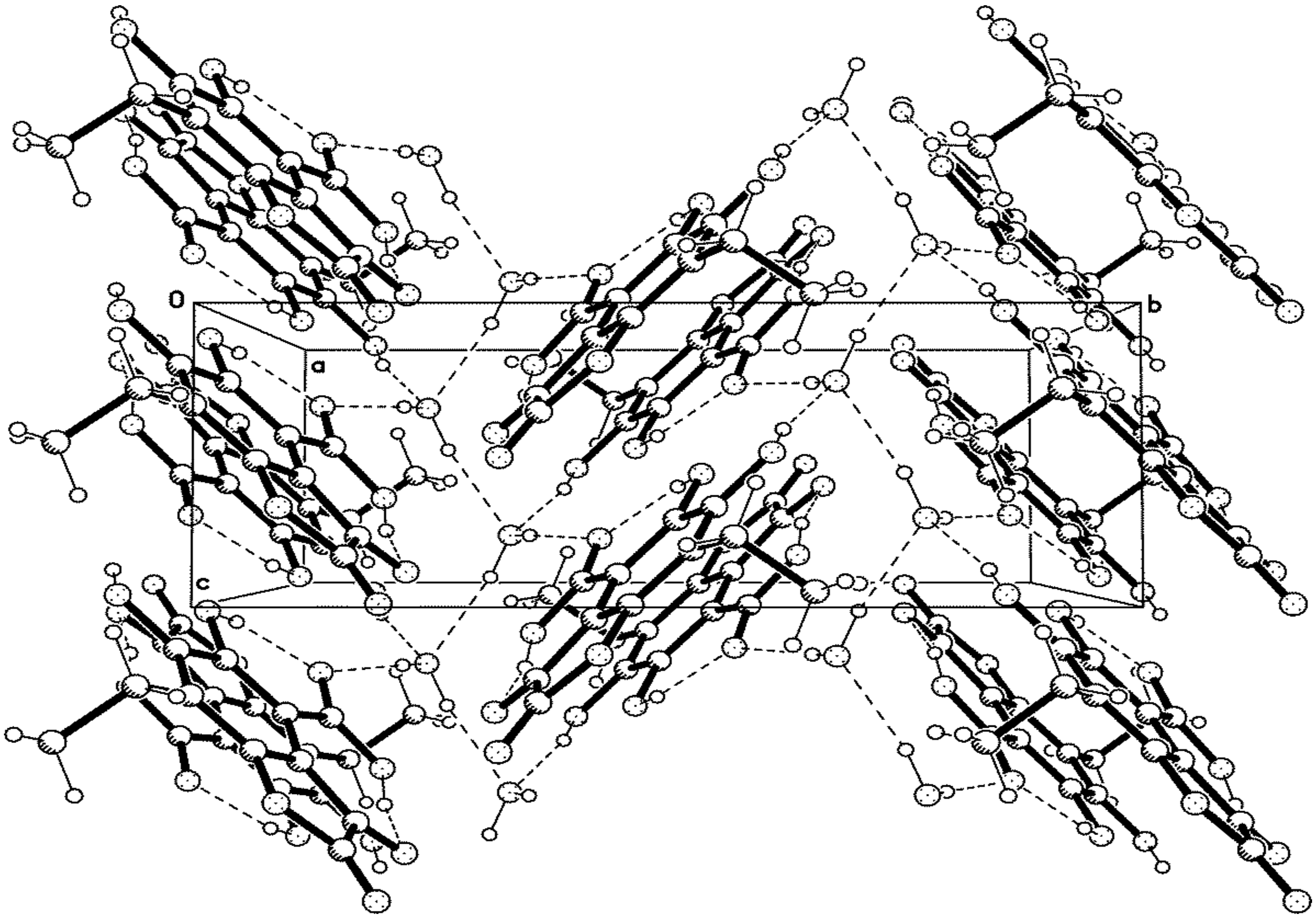

Figure S21. Overall packing for α-$C_{11}H_8O_7 \cdot H_2O$ viewed along the *a*-axis direction (hydrogen bonds are shown as dashed lines).

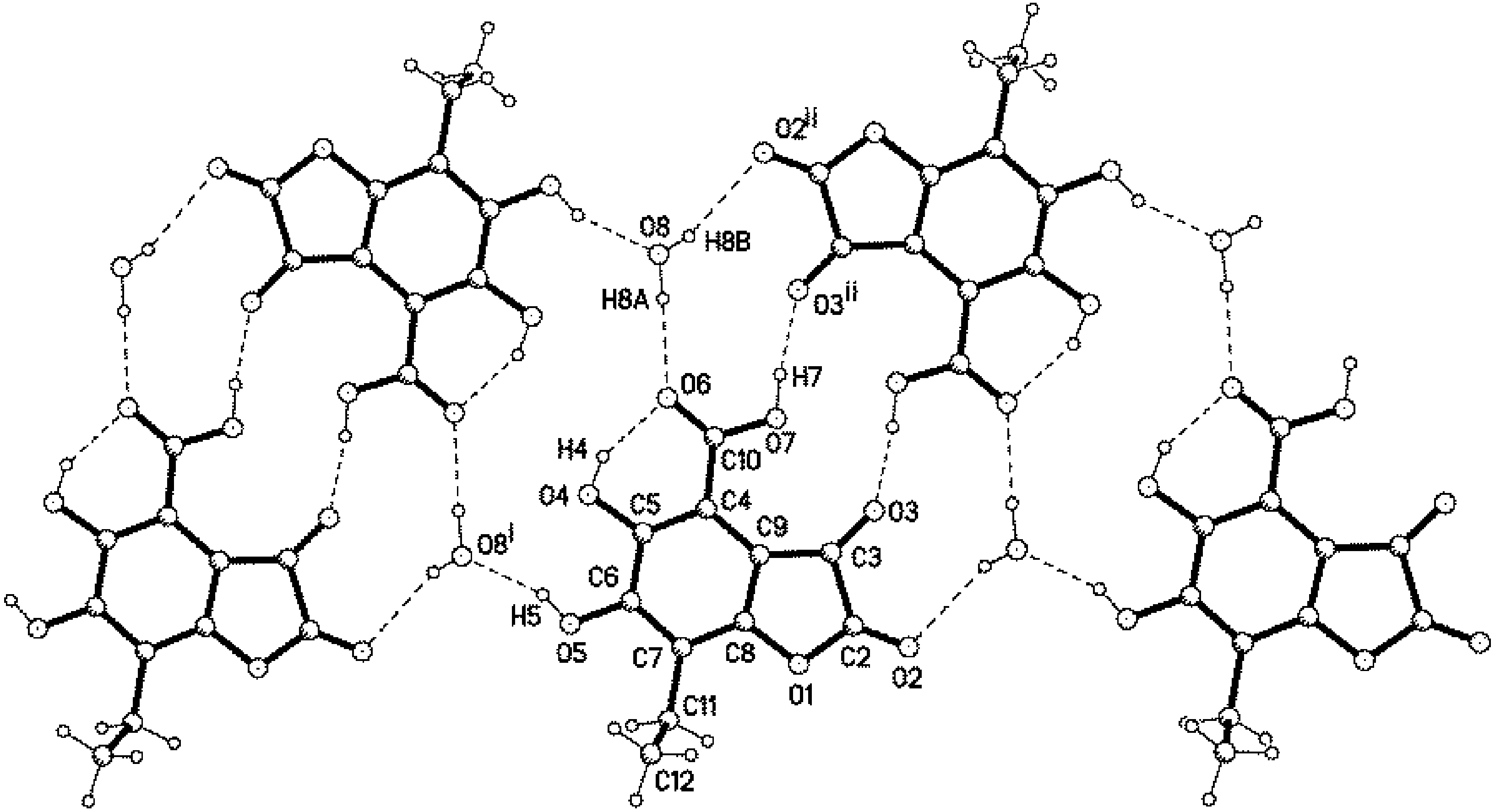

Figure S22. A plot of bonds for β-$C_{11}H_8O_7 \cdot H_2O$ (hydrogen bonds are shown as dashed lines).

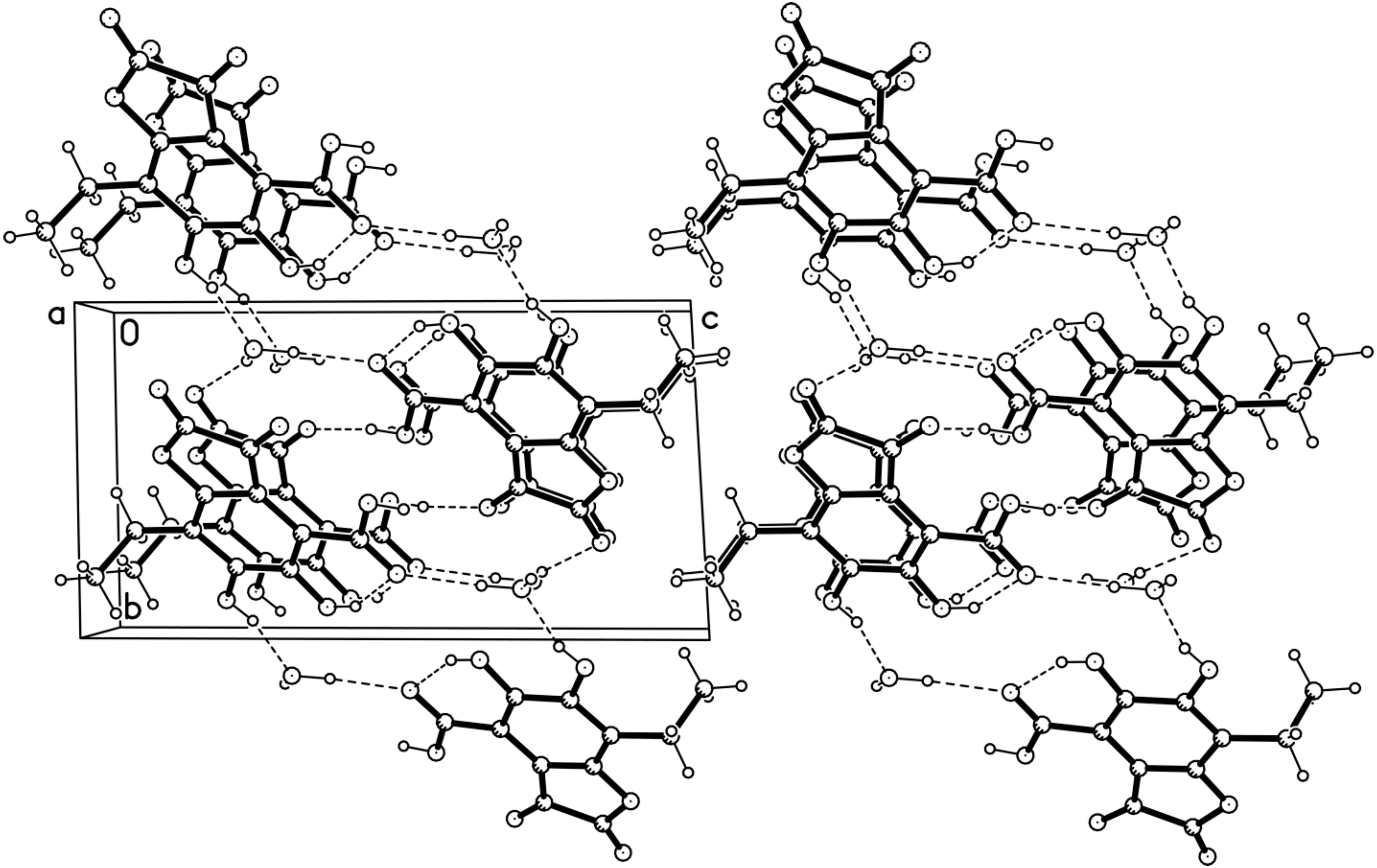

Figure S23. Overall packing for β-$C_{11}H_8O_7 \cdot H_2O$ viewed along the *a*-axis direction (hydrogen bonds are shown as dashed lines).

The key NMR acquisition parameters shown in the spectra below are as follows: NS, number of scans; DS, number of dummy scans; FIDRES, FID resolution; SWH, spectral width in Hz; AQ, acquisition time in s; RG, receiver gain; DW, dwell time; DE, pre-scan delay; TE, demand temperature on the temperature unit; D1, array of delays; TD, time domain; number of raw data points; P (μsec), array of pulse lengths; PLW (W), array of power levels; SFO1, irradiation (carrier) frequencies; CNST, array of constants used in pulse programs.

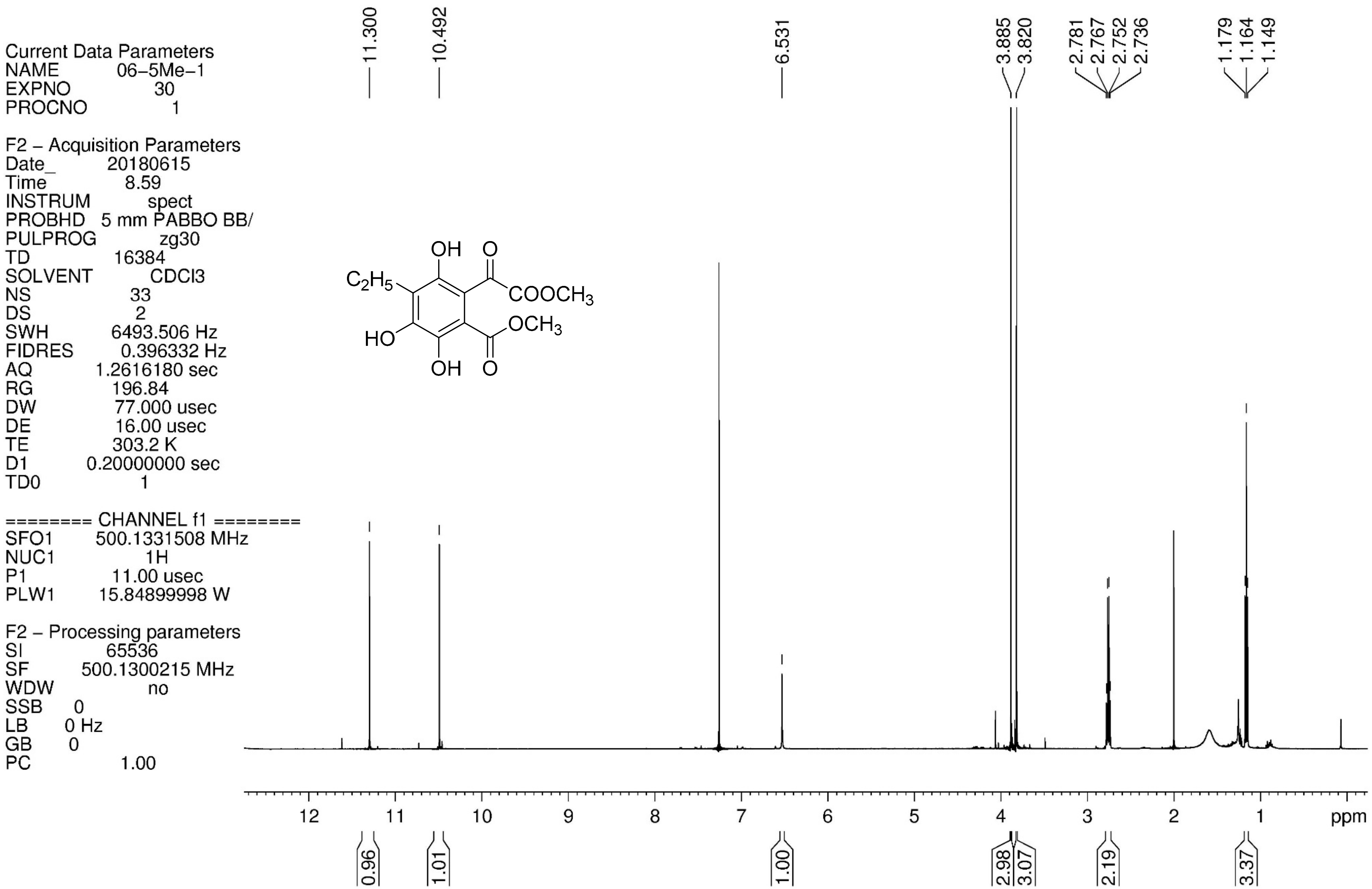

Figure S24. ^{1}H-NMR spectrum (500 MHz, $CDCl_3$) of dimethyl ether of compound **7.**

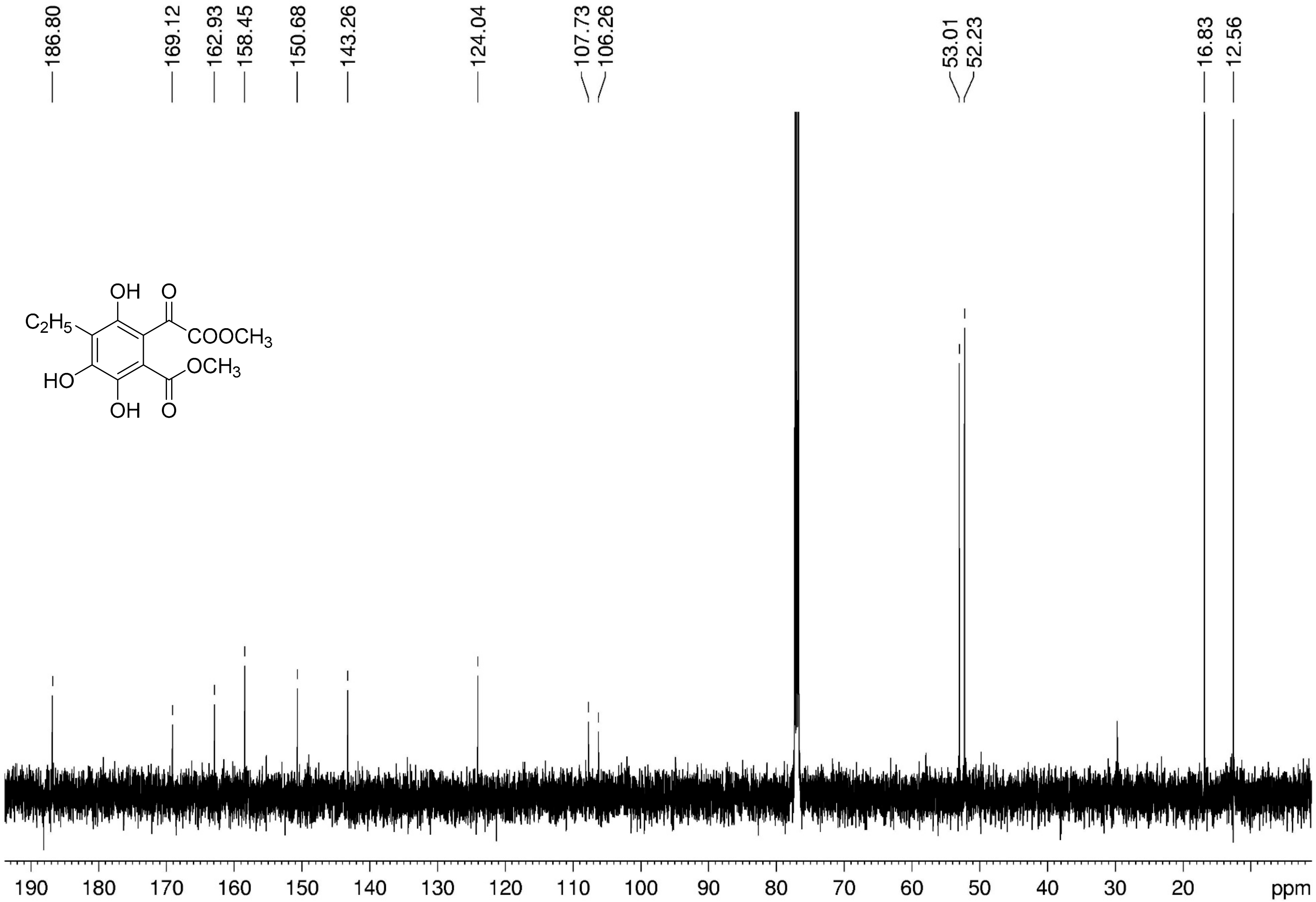

Figure S25. ^{13}C-NMR spectrum (126 MHz, $CDCl_3$) of dimethyl ether of compound **7.**

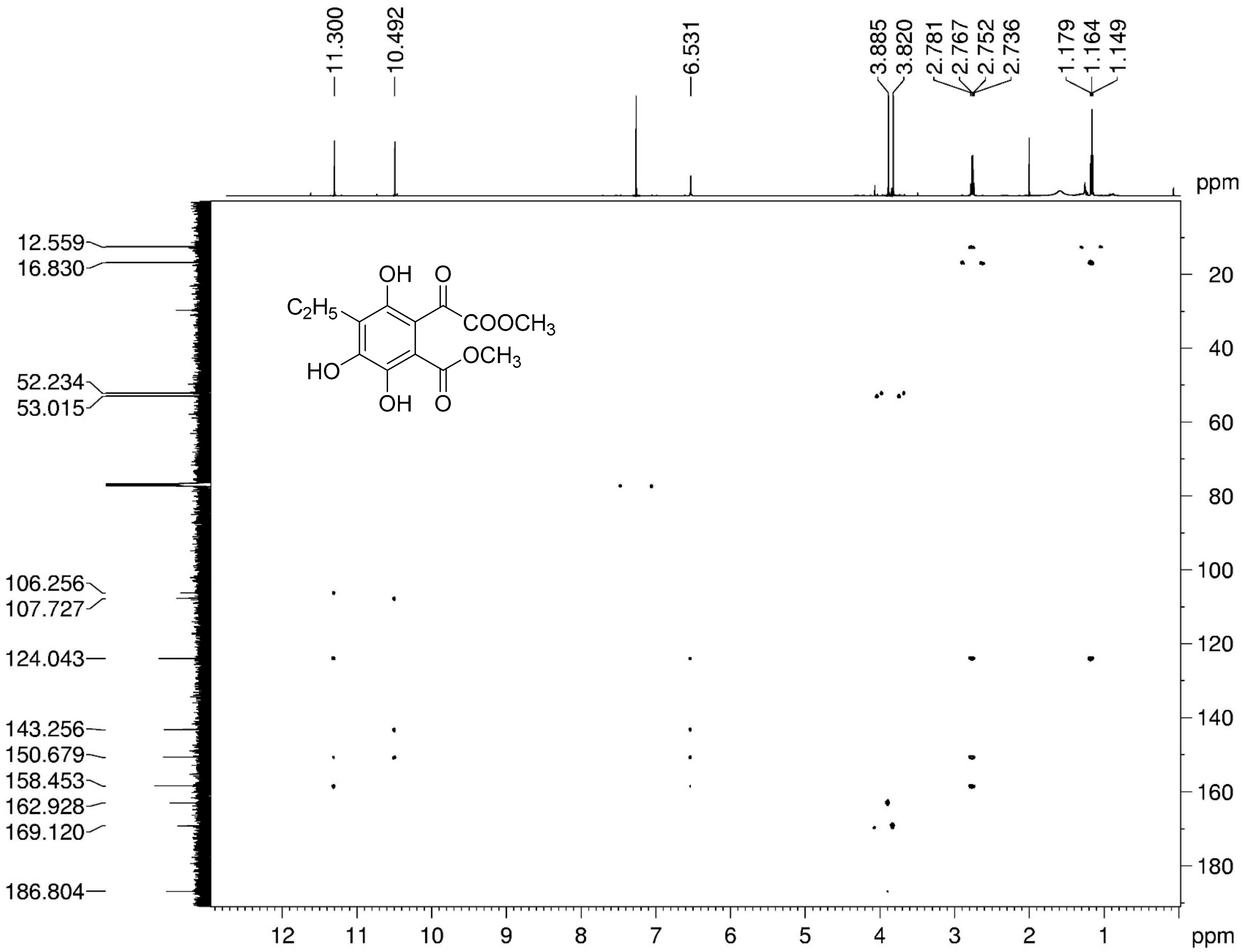

Figure S26. HMBC spectrum (500 MHz, $CDCl_3$) of dimethyl ether of compound **7.**

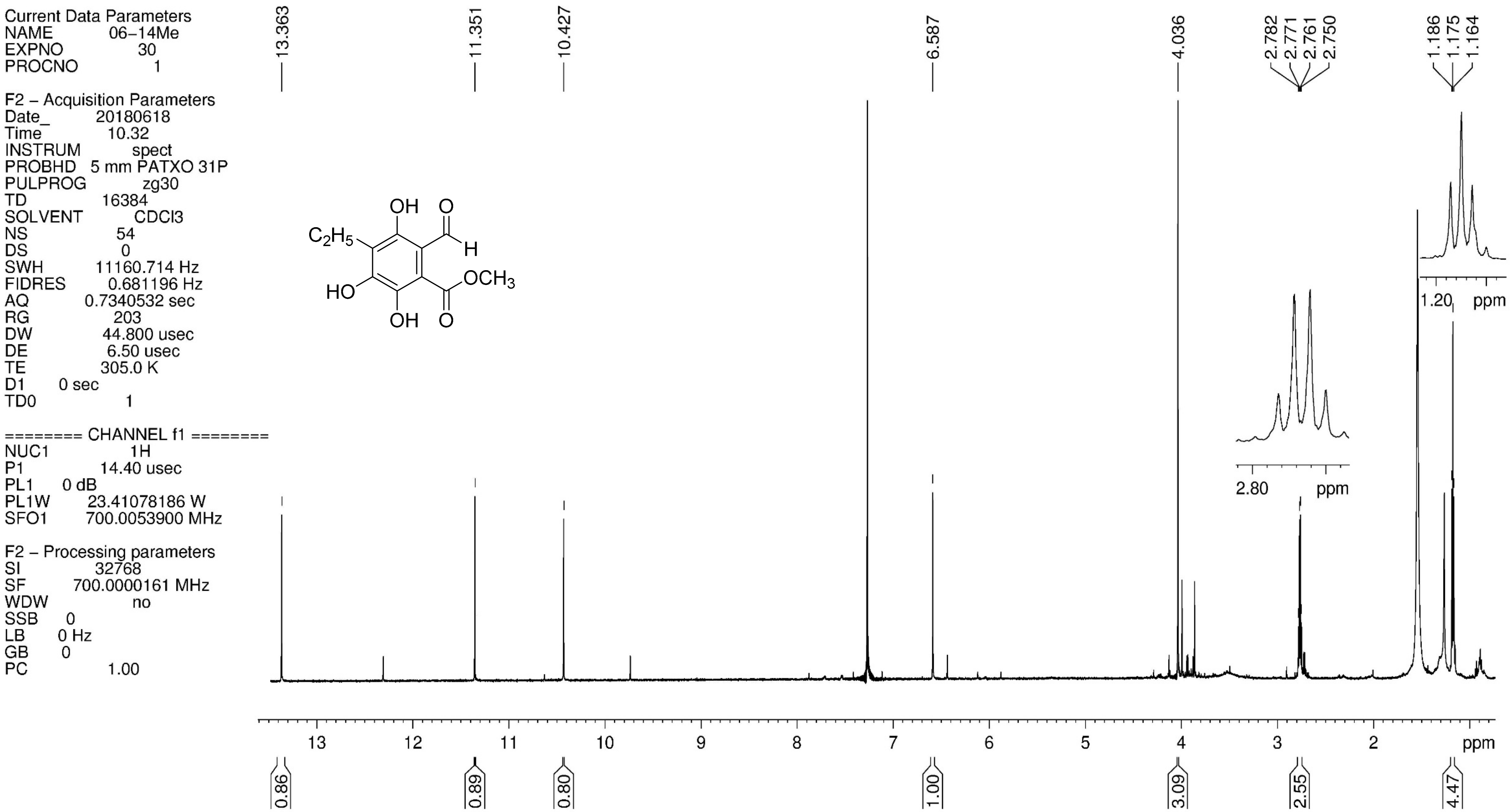

Figure S27. ¹H-NMR spectrum (700 MHz, $CDCl_3$) of methyl ether of 4-ethyl-2-formyl-3,5,6-trihydroxybenzoic acid **(8).**

Current Data Parameters
NAME 06–14Me
EXPNO 3730
PROCNO 1

F2 – Acquisition Parameters
Date_ 20180618
Time 10.42
INSTRUM spect
PROBHD 5 mm PATXO 31P
PULPROG zgpg
TD 65536
SOLVENT CDCl3
NS 25024
DS 2
SWH 42613.637 Hz
FIDRES 0.650232 Hz
AQ 0.7690057 sec
RG 203
DW 11.733 usec
DE 6.50 usec
TE 305.9 K
D1 2.00000000 sec
D11 0.03000000 sec
TD0 4096

======== CHANNEL f1 ========
NUC1 13C
P1 9.10 usec
PL1 0 dB
PL1W 106.75517273 W
SFO1 176.0353807 MHz

======== CHANNEL f2 ========
CPDPRG2 waltz16
NUC2 1H
PCPD2 71.90 usec
PL2 0 dB
PL12 13.05 dB
PL13 17.00 dB
PL2W 23.41078186 W
PL12W 1.15988755 W
PL13W 0.46710649 W
SFO2 700.0035000 MHz

F2 – Processing parameters
SI 65536
SF 176.0151325 MHz
WDW EM
SSB 0
LB 1.00 Hz
GB 0
PC 1.40

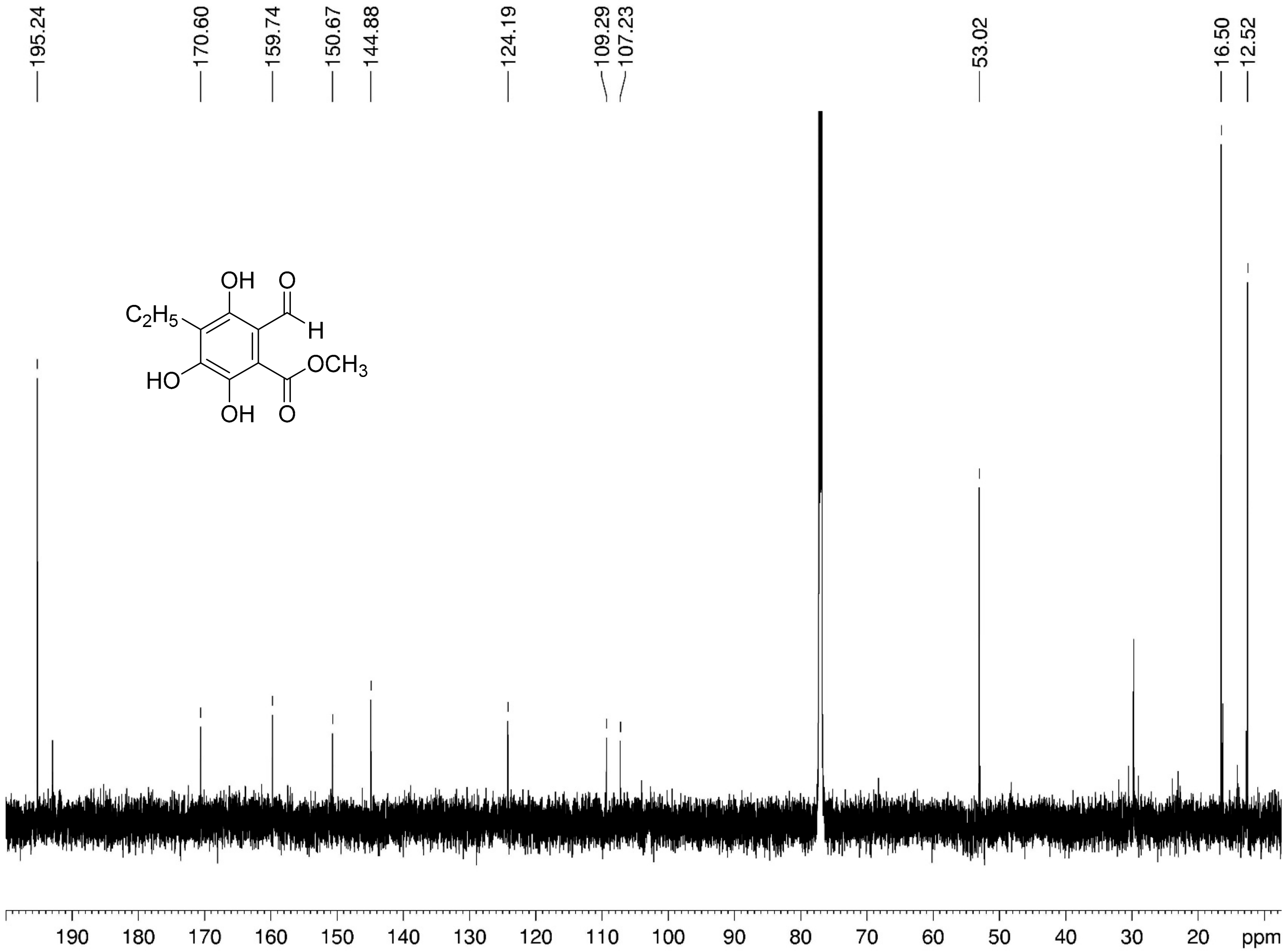

Figure S28. ^{13}C-NMR spectrum (175 MHz, $CDCl_3$) of methyl ether of **8.**

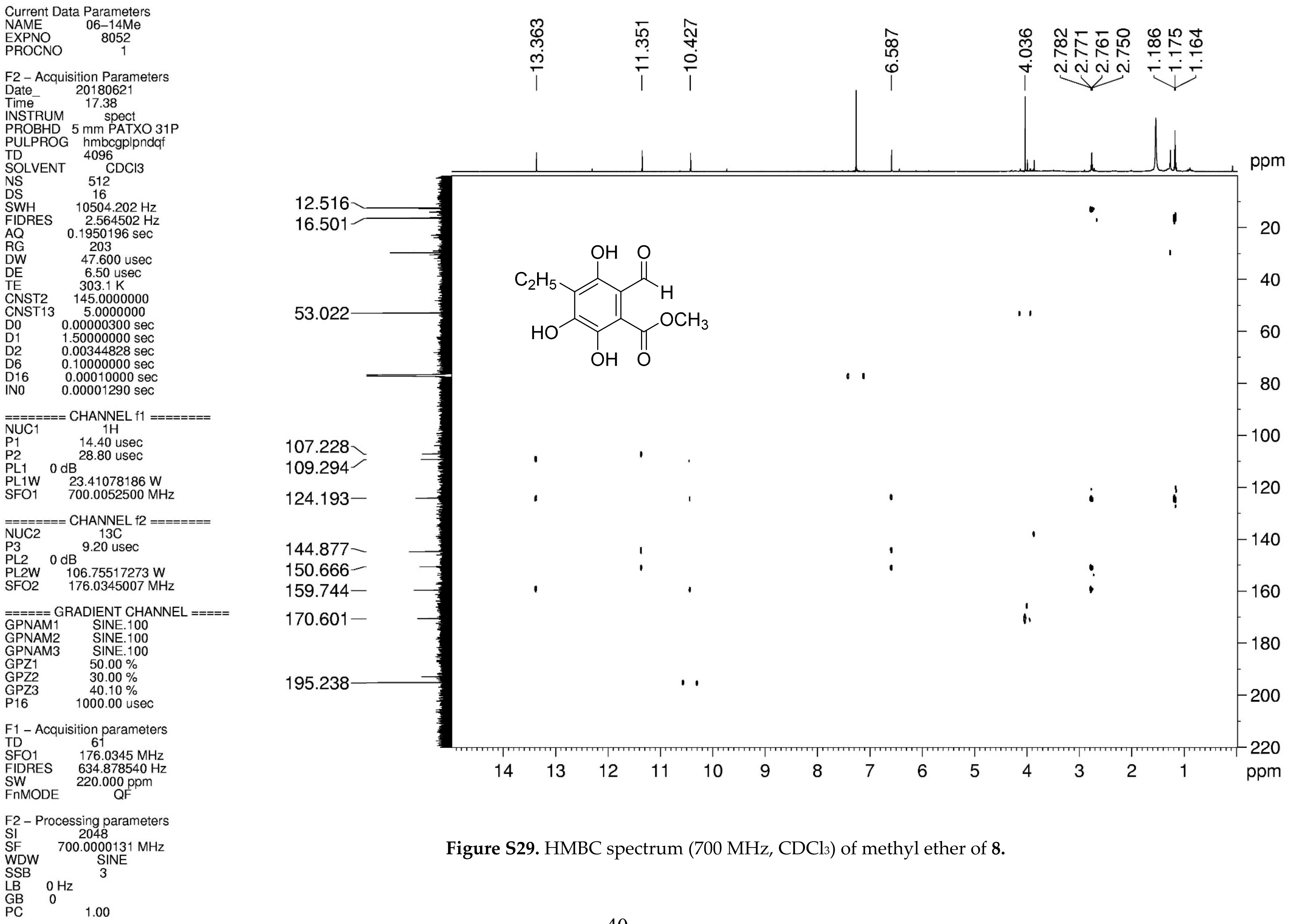

Figure S29. HMBC spectrum (700 MHz, CDCl$_3$) of methyl ether of **8.**

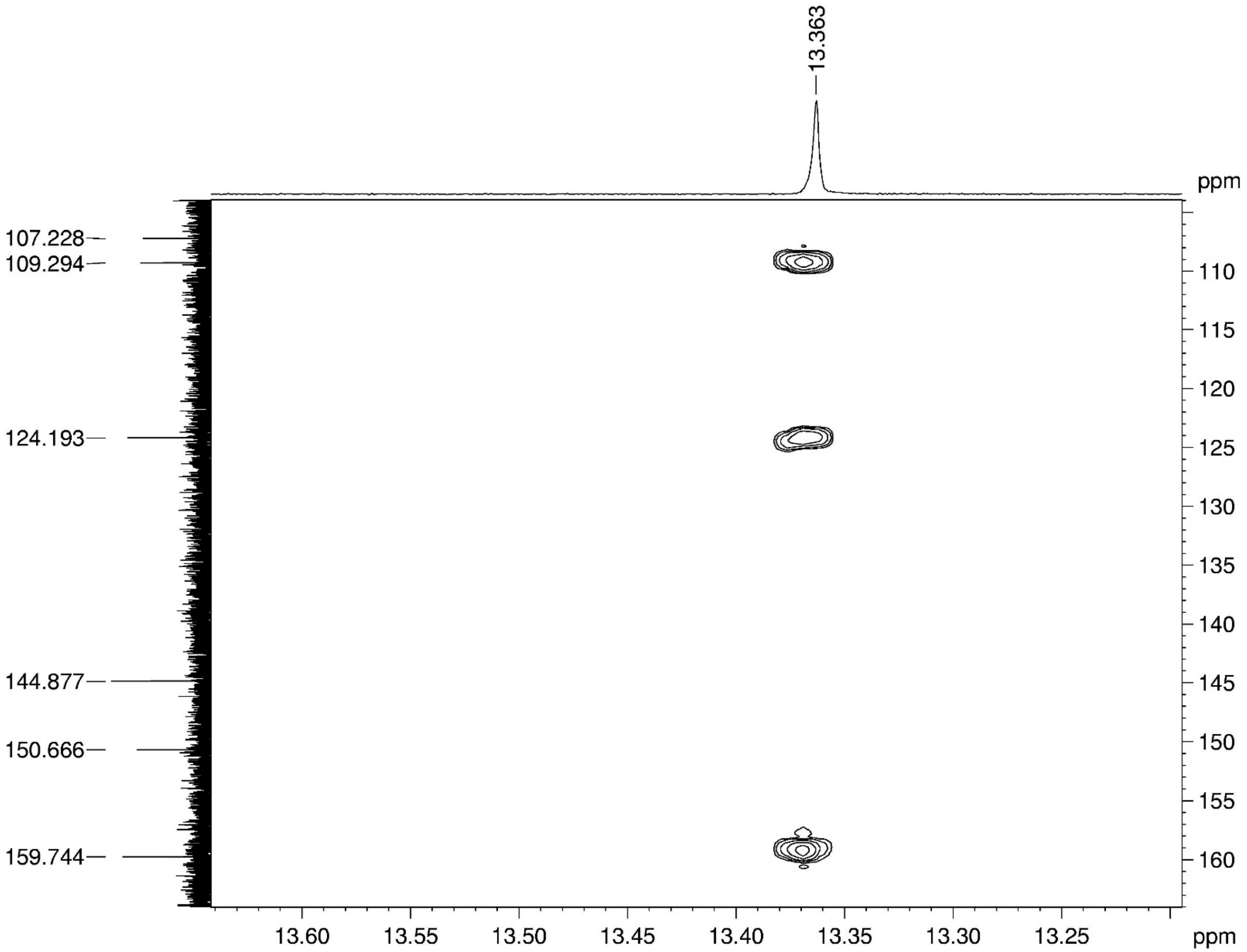

Figure S30. HMBC correlations of methyl ether of **8** (enlarged).

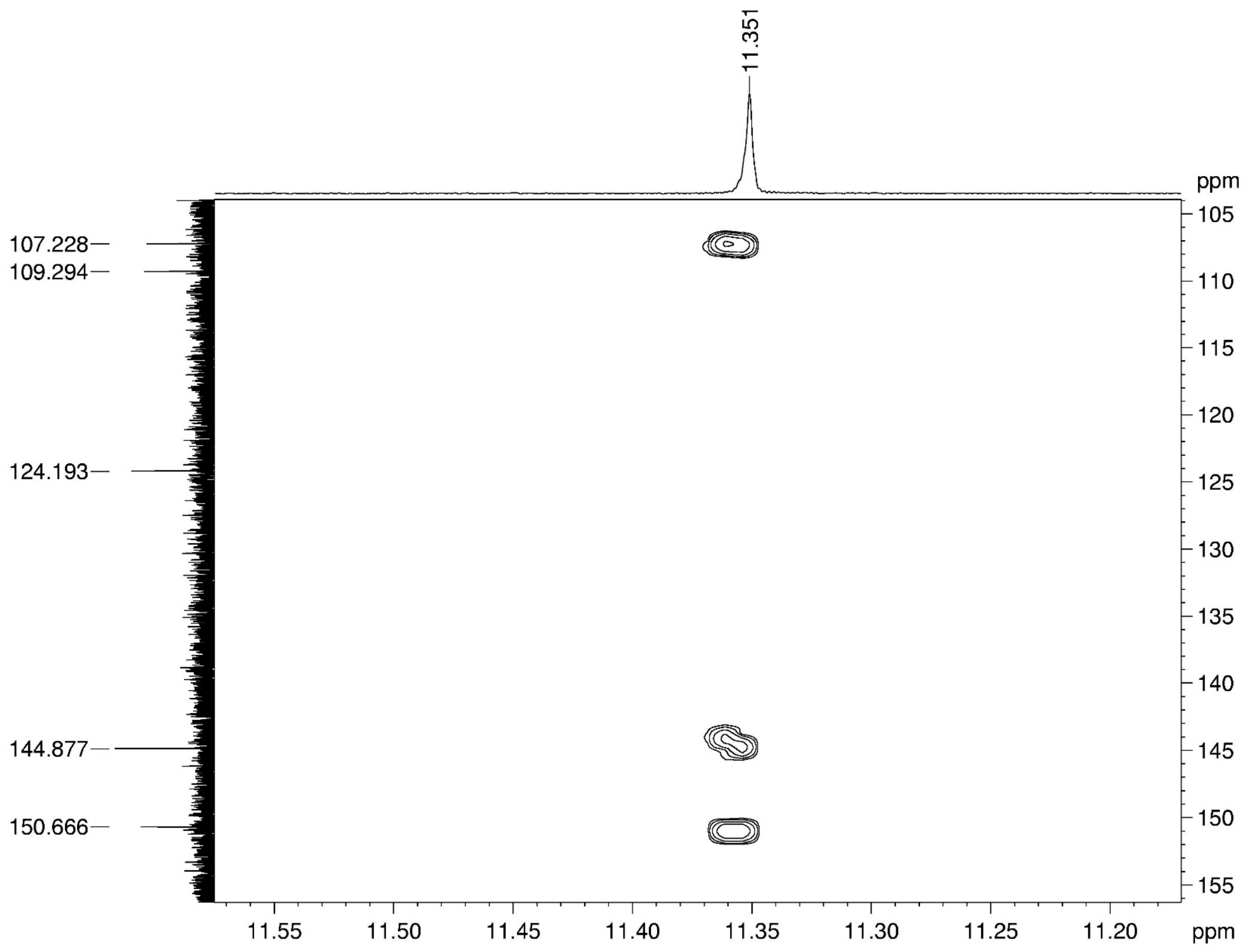

Figure S31. HMBC correlations of methyl ether of **8** (enlarged).

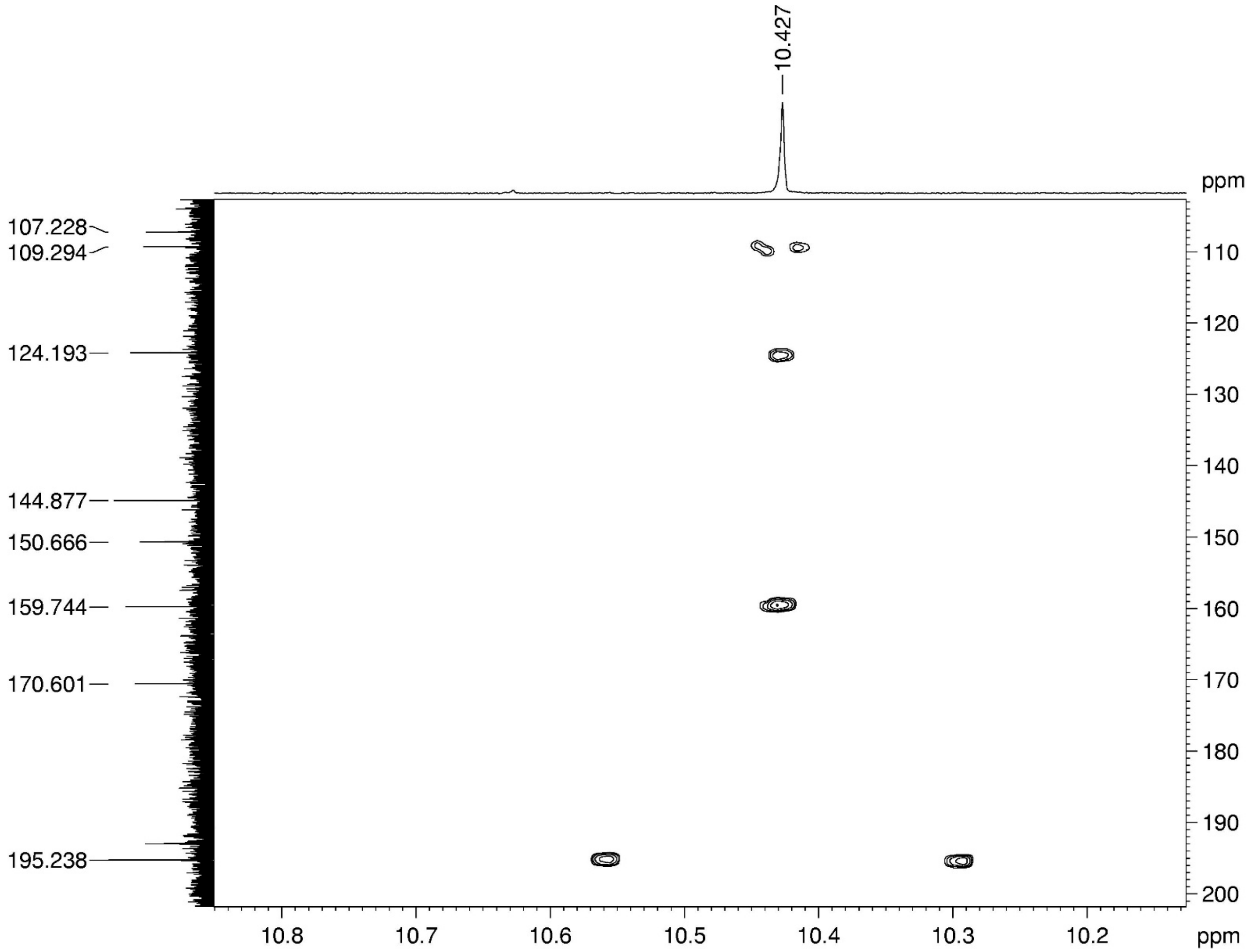

Figure S32. HMBC correlations of methyl ether of **8** (enlarged).

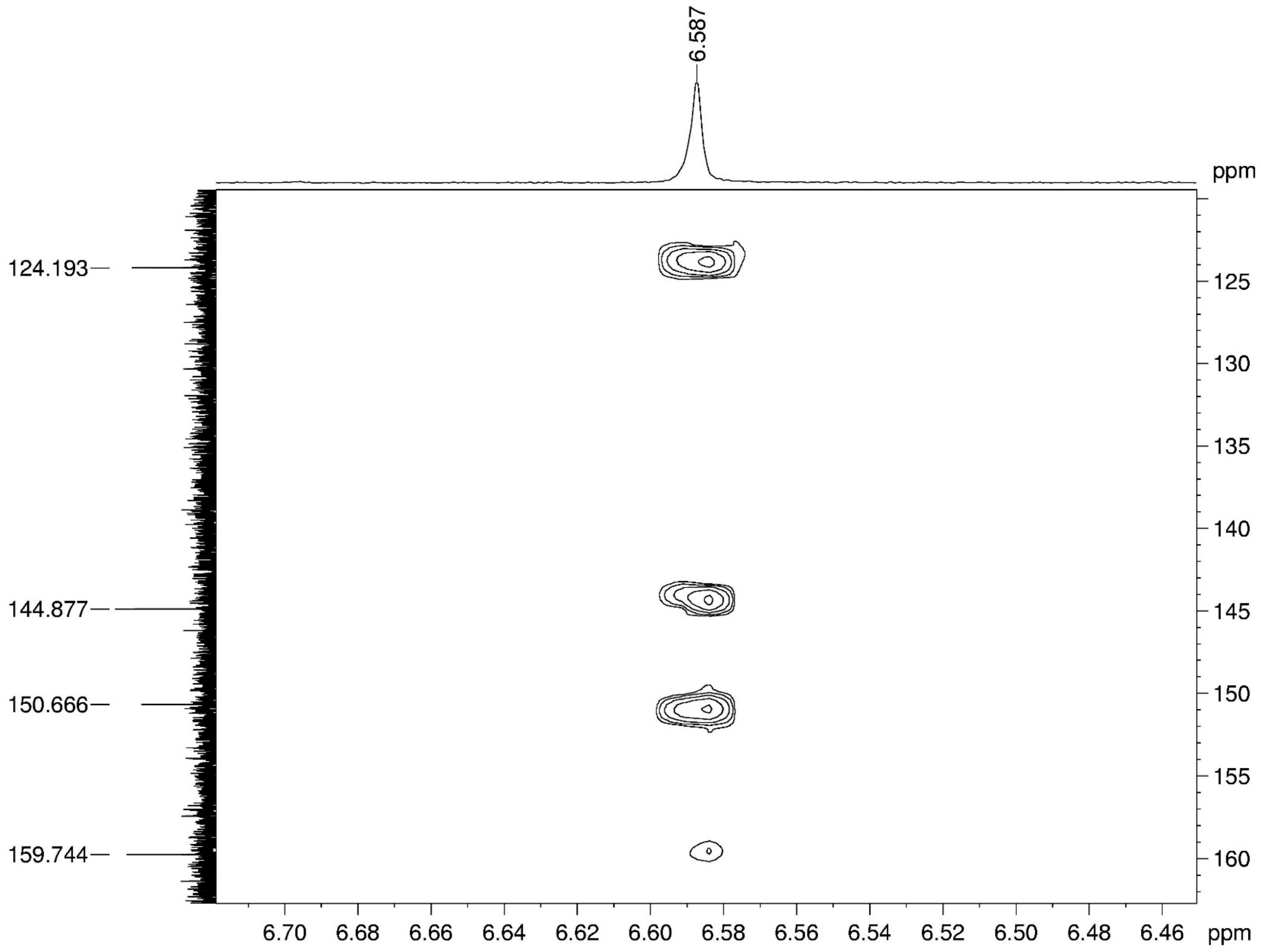

Figure S33. HMBC correlations of methyl ether of **8** (enlarged).

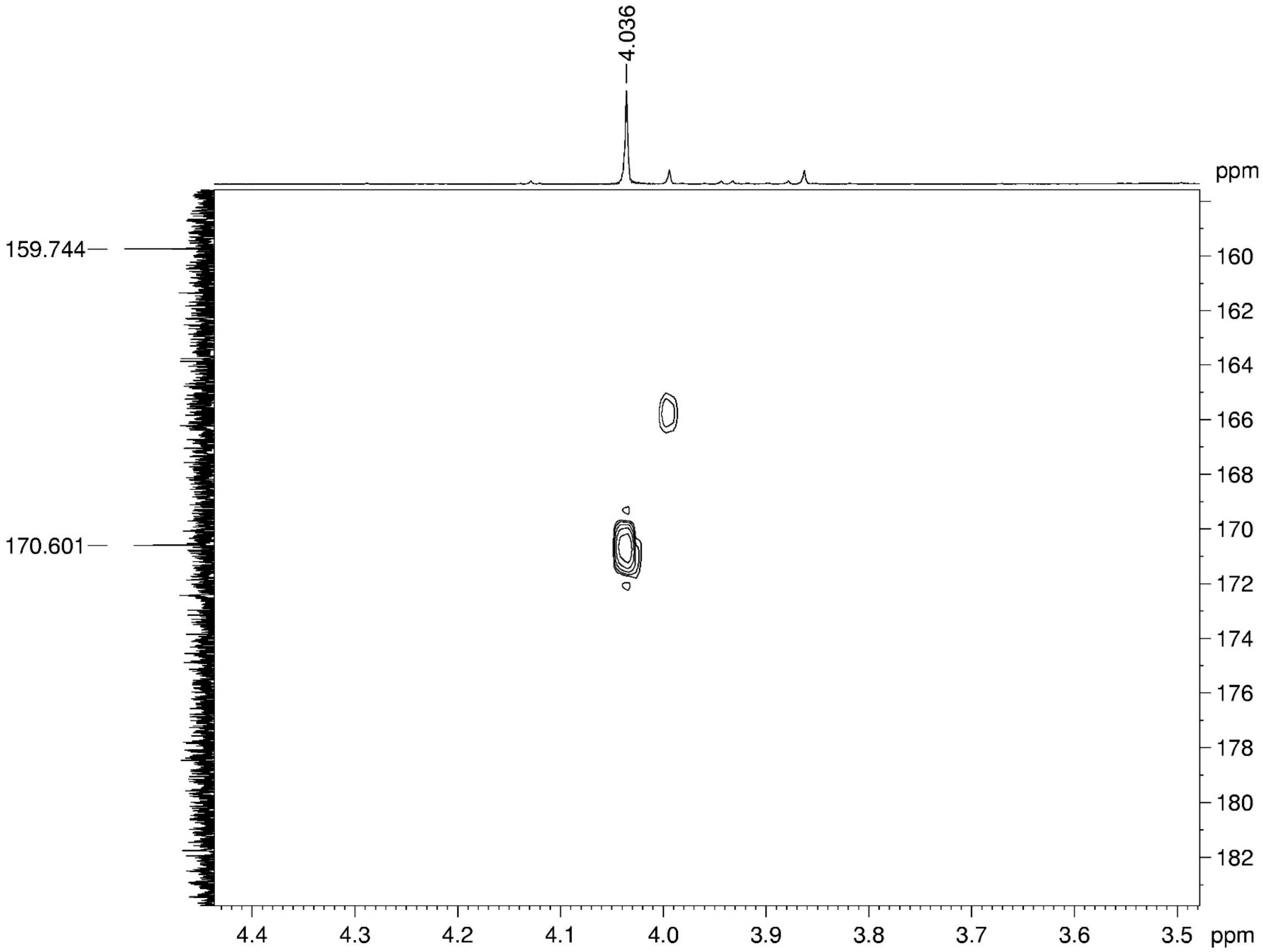

Figure S34. HMBC correlations of methyl ether of **8** (enlarged).

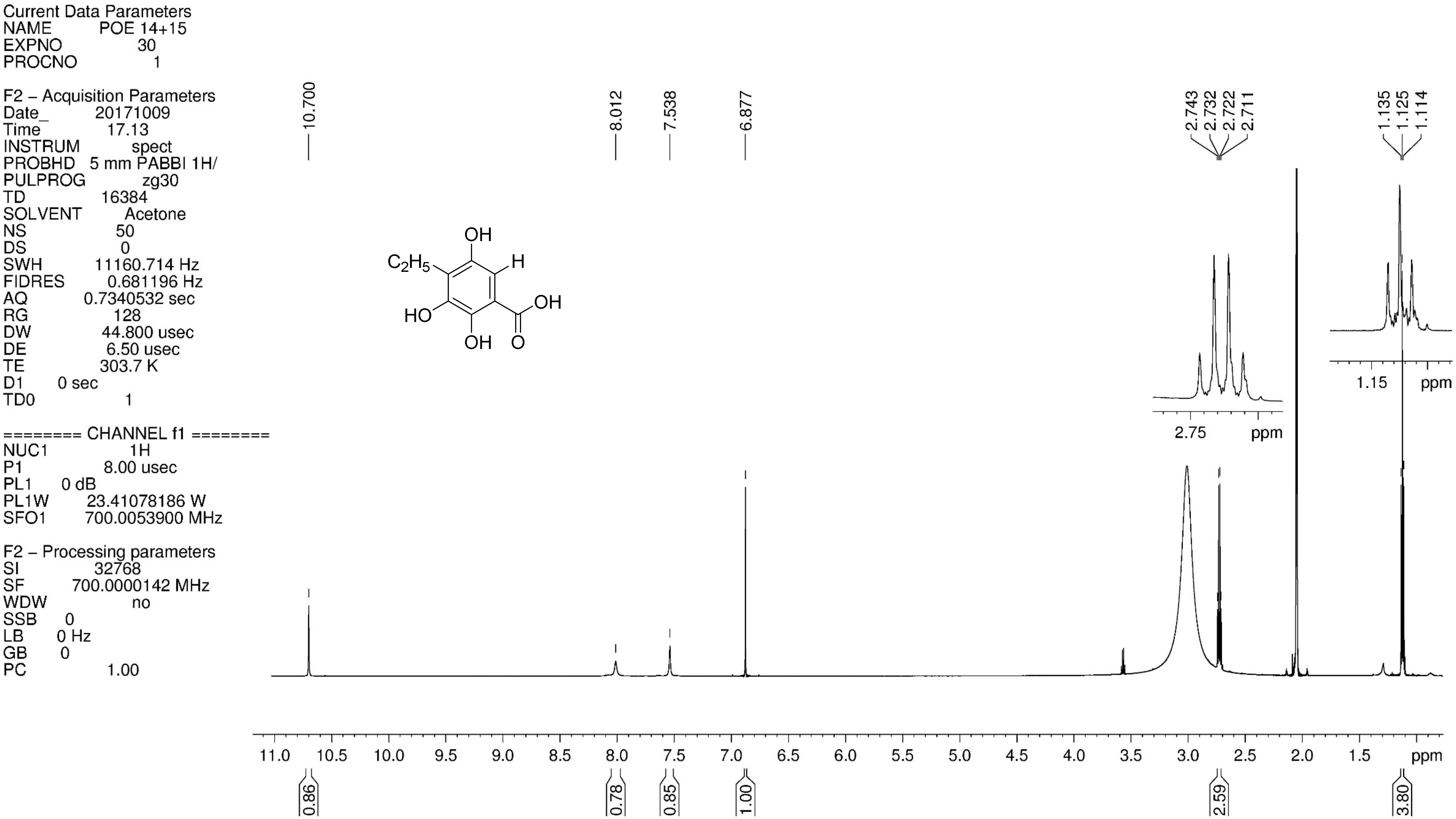

Figure S35. ^{1}H-NMR spectrum (700 MHz, acetone-d_6) of 4-ethyl-2,3,5-trihydroxybenzoic acid **(9).**

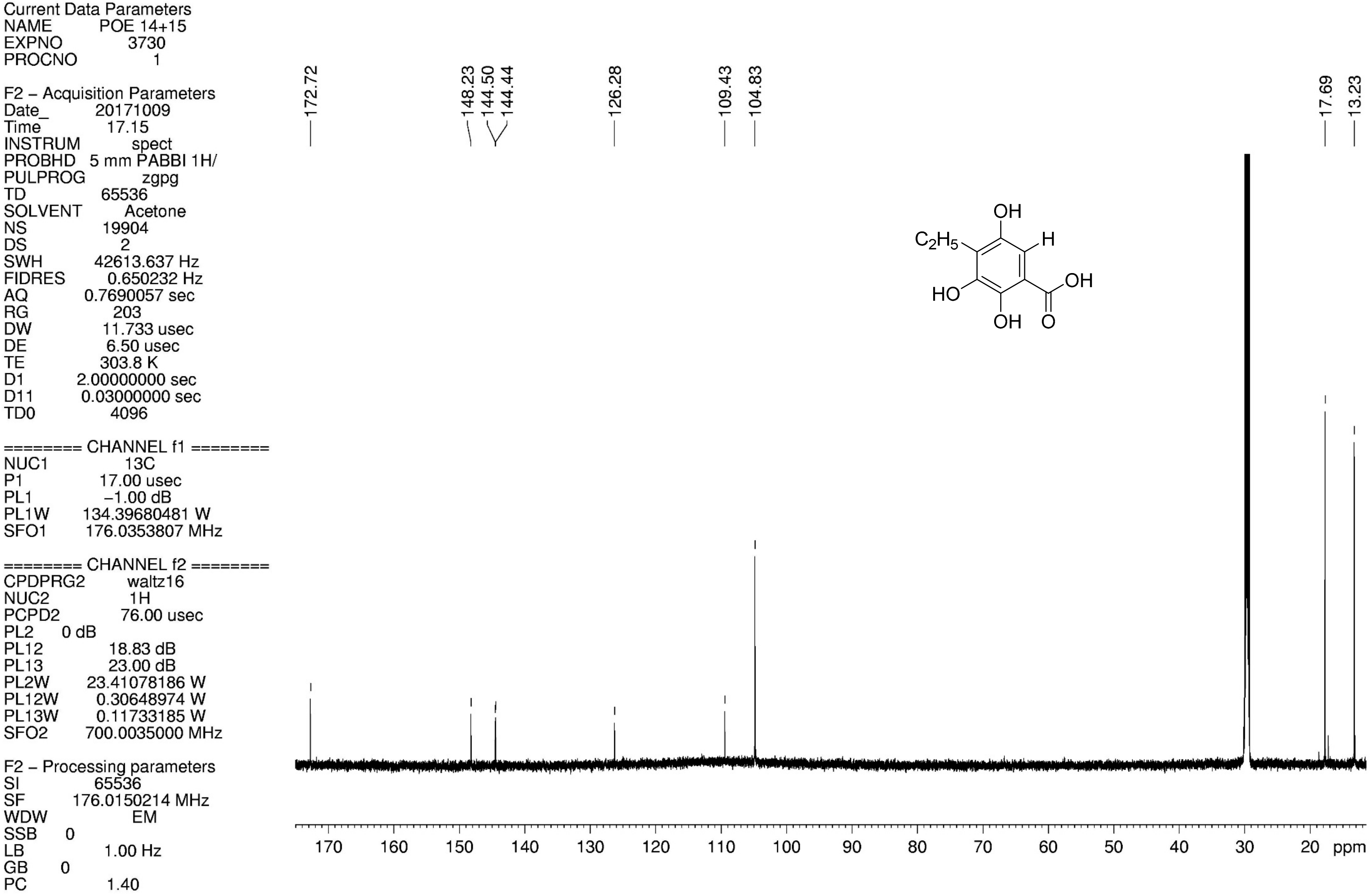

Figure S36. ^{13}C NMR spectrum (175 MHz, acetone-d_6) of **9.**

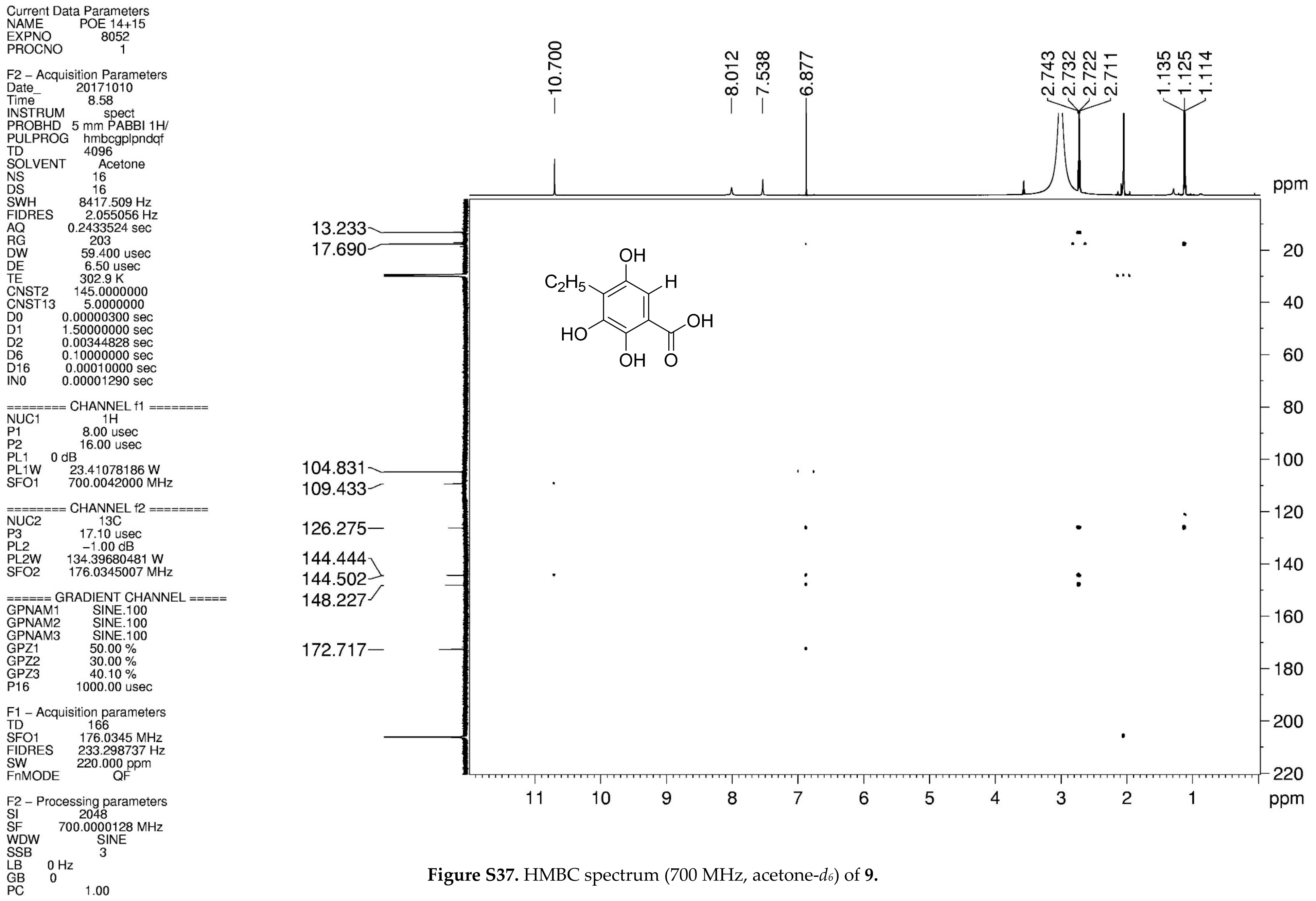

Figure S37. HMBC spectrum (700 MHz, acetone-d_6) of **9.**

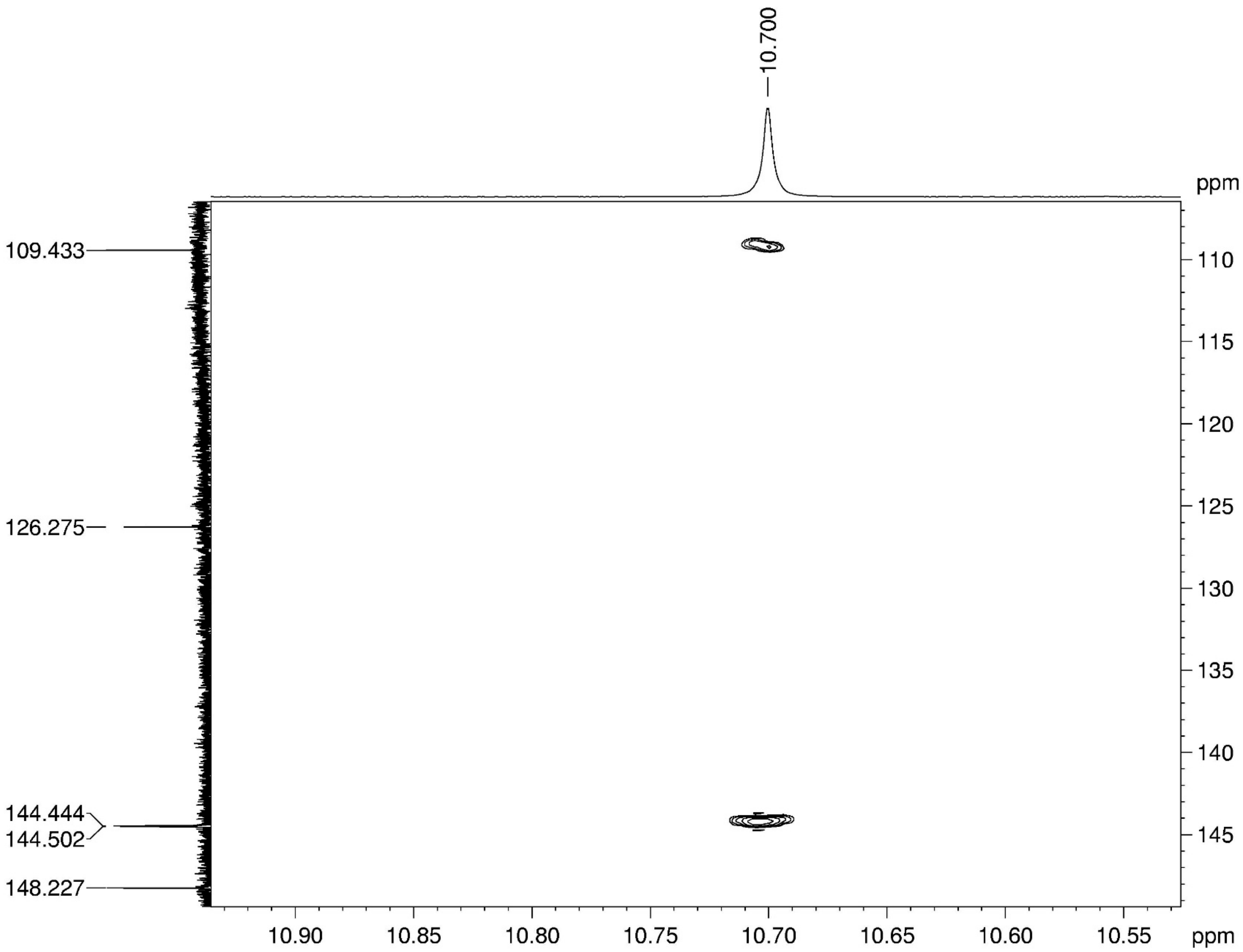

Figure S38. HMBC correlations of **9** (enlarged).

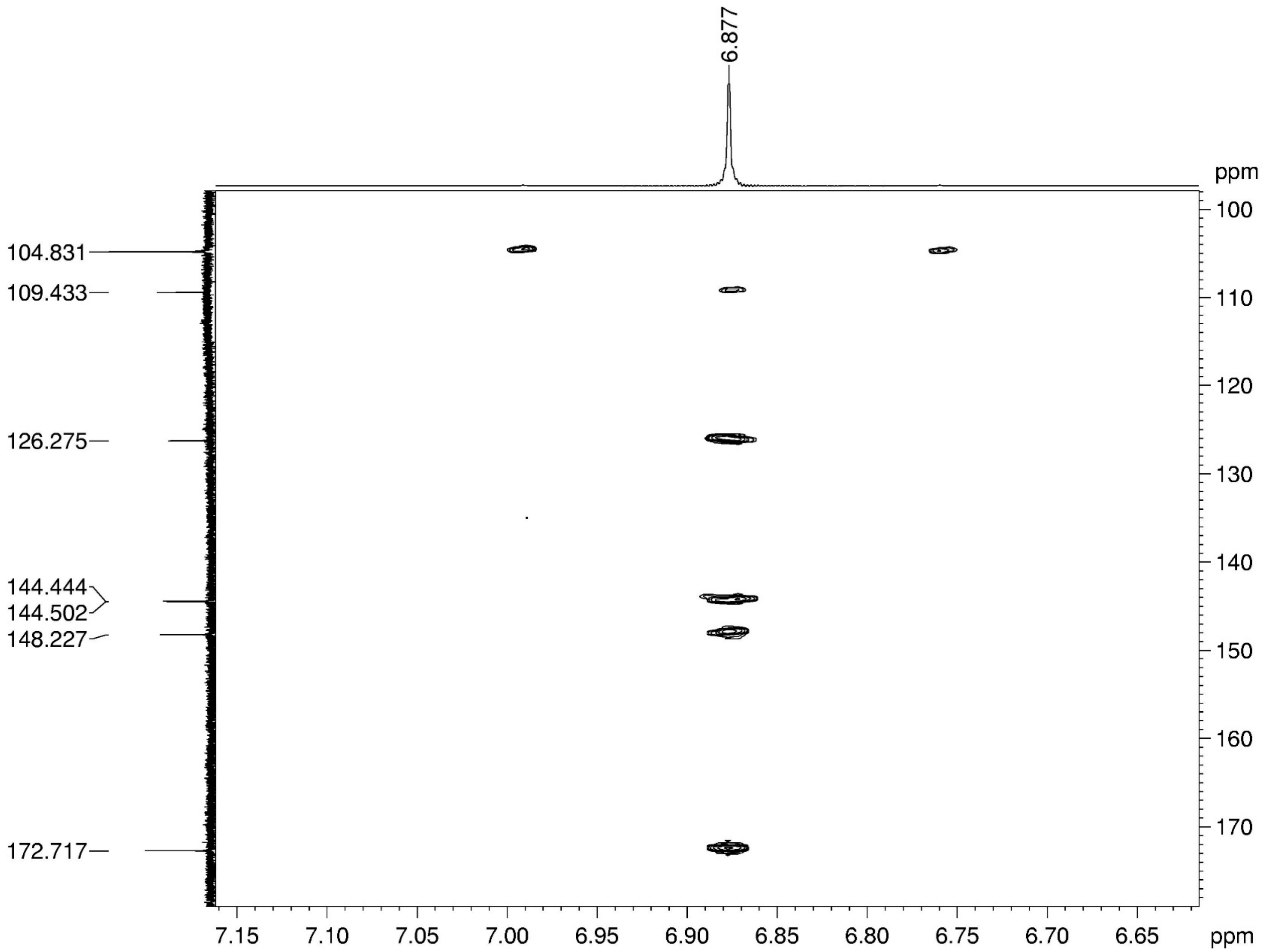

Figure S39. HMBC correlations of **9** (enlarged).

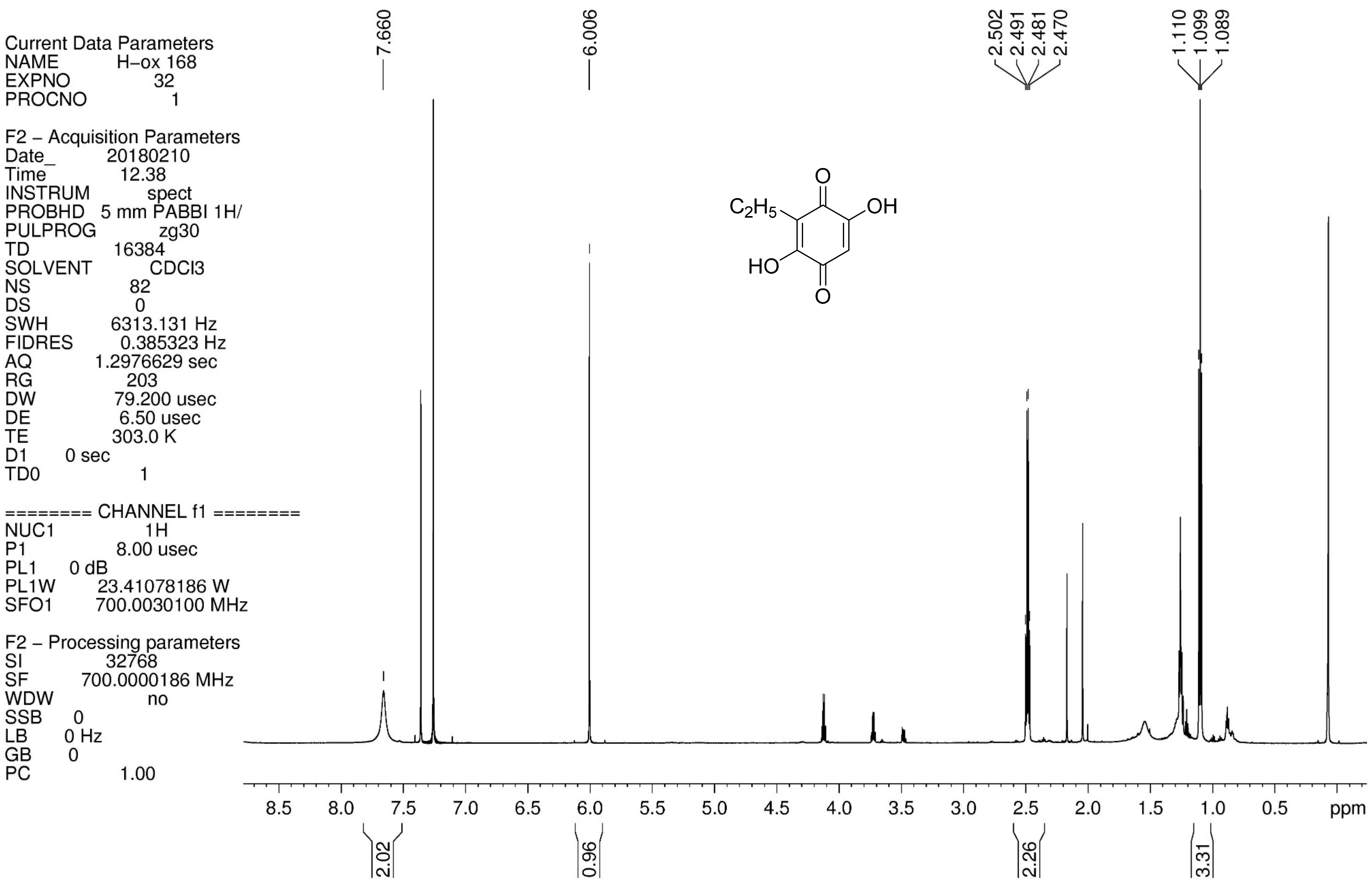

Figure S40. ^{1}H-NMR spectrum (700 MHz, $CDCl_3$) of 3-ethyl-2,5-dihydroxy-1,4-benzoquinone **(10).**

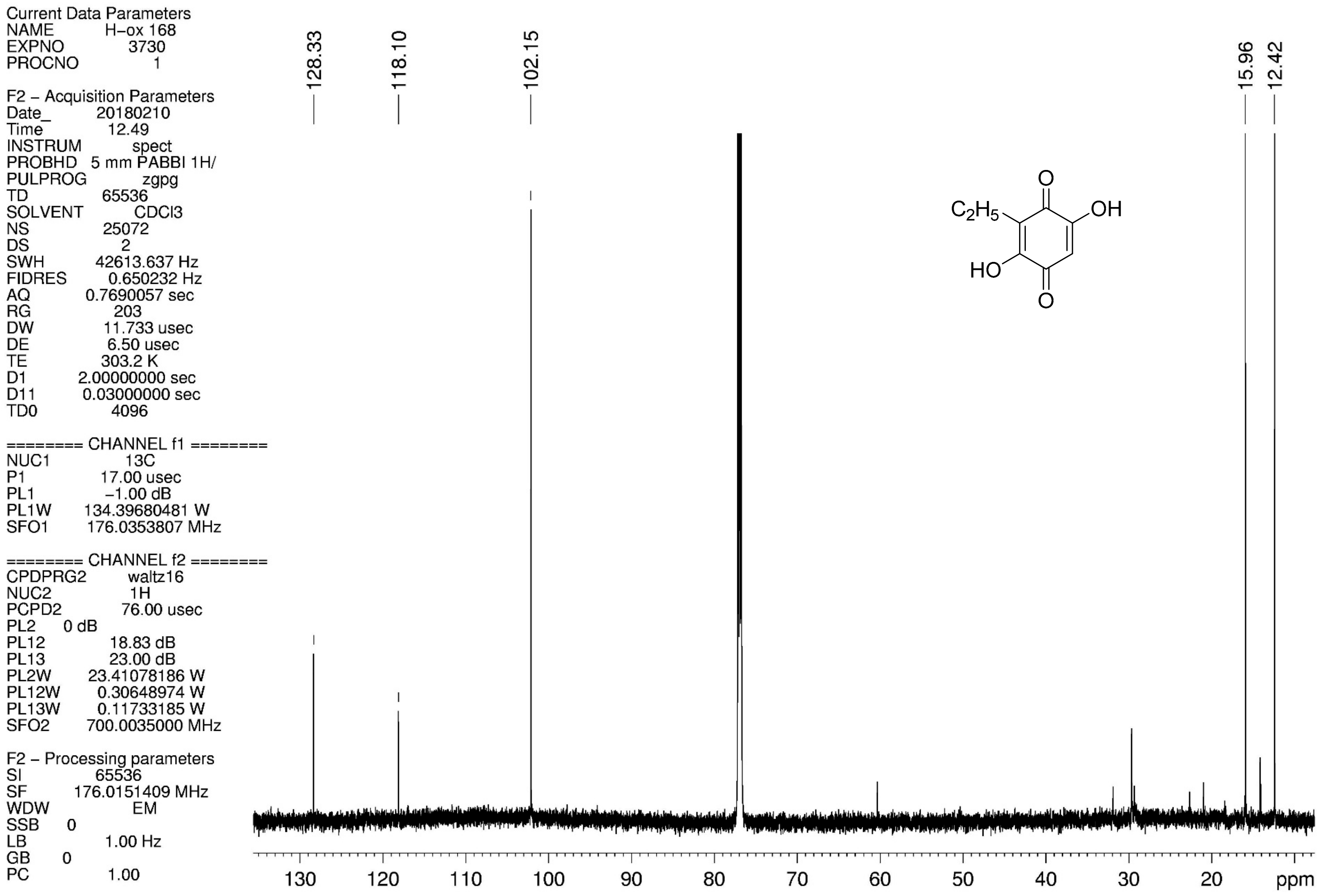

Figure S41. ^{13}C-NMR spectrum (175 MHz, $CDCl_3$) of **10.**

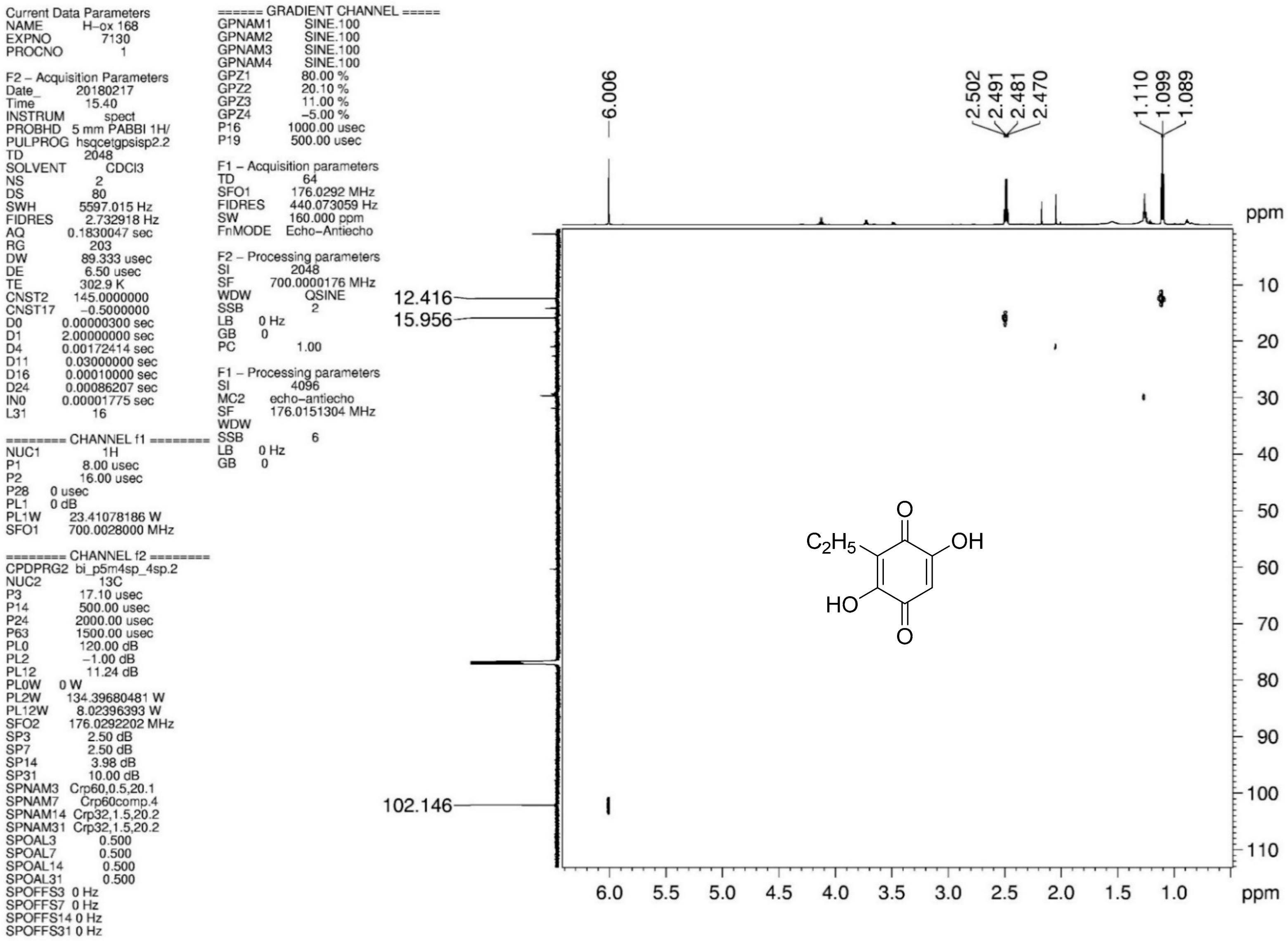

Figure S42. HSQC spectrum (700 MHz, $CDCl_3$) of **10.**

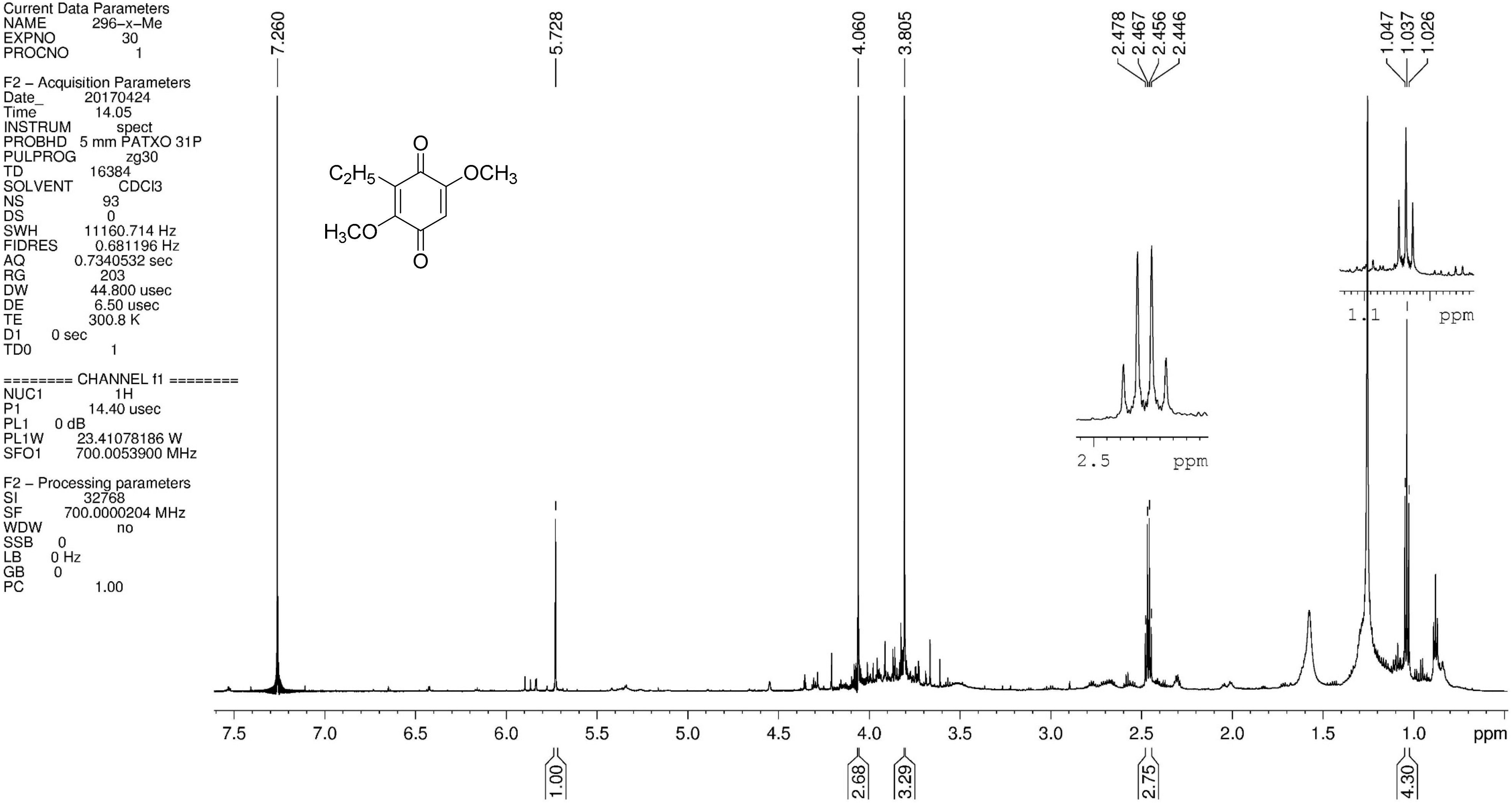

Figure S43. ^{1}H NMR spectrum (700 MHz, $CDCl_3$) of dimethyl ether of **10.**

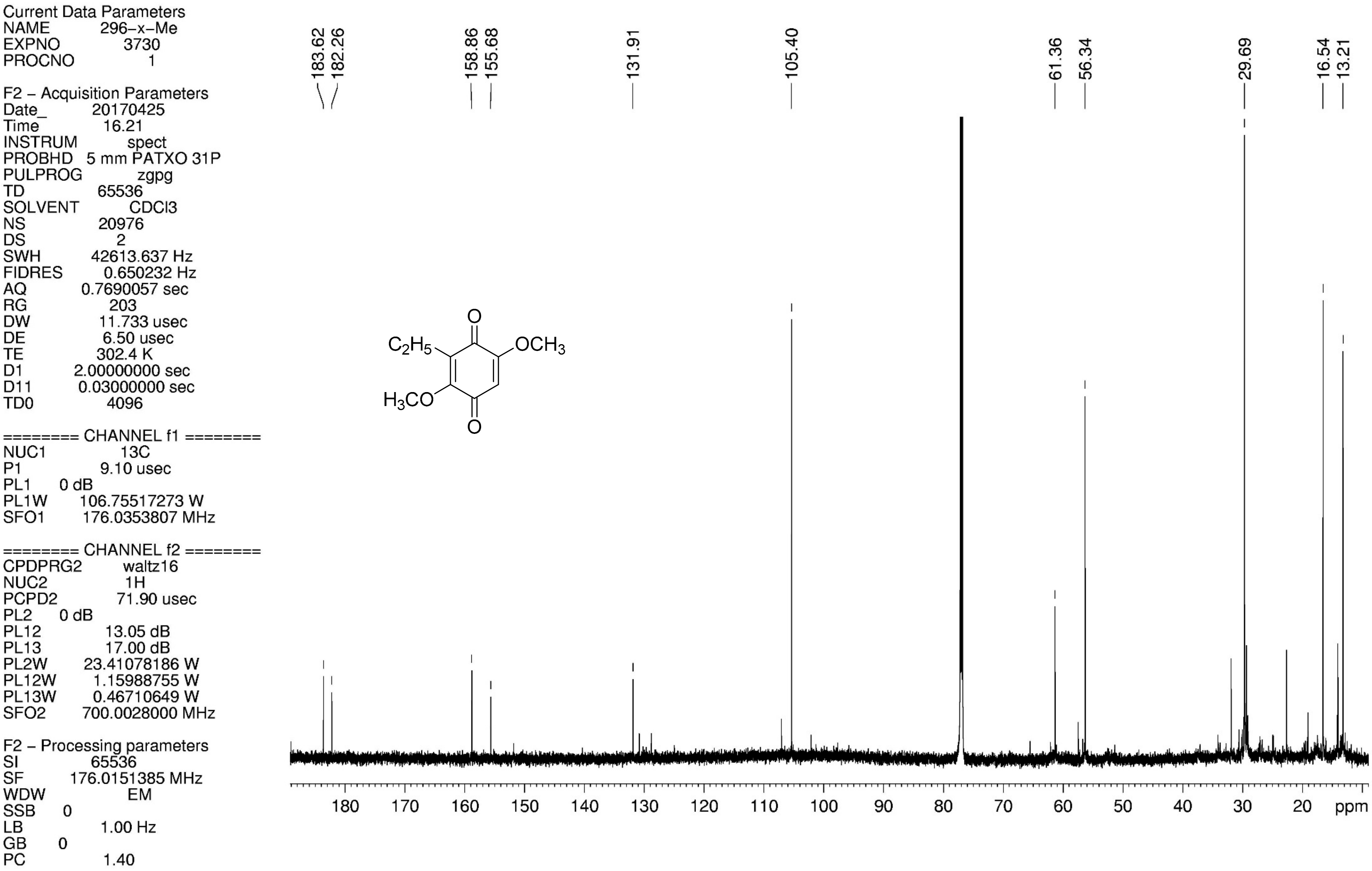

Figure S44. ^{13}C-NMR spectrum (175 MHz, $CDCl_3$) of dimethyl ether of **10.**

Current Data Parameters
NAME 296–x–Me
EXPNO 8052
PROCNO 1

F2 – Acquisition Parameters
Date_ 20170426
Time 16.58
INSTRUM spect
PROBHD 5 mm PATXO 31P
PULPROG hmbcgplpndqf
TD 4096
SOLVENT CDCl3
NS 160
DS 16
SWH 5597.015 Hz
FIDRES 1.366459 Hz
AQ 0.3659593 sec
RG 203
DW 89.333 usec
DE 6.50 usec
TE 302.0 K
CNST2 145.0000000
CNST13 5.0000000
D0 0.00000300 sec
D1 1.20000005 sec
D2 0.00344828 sec
D6 0.10000000 sec
D16 0.00010000 sec
IN0 0.00001420 sec

======== CHANNEL f1 ========
NUC1 1H
P1 14.40 usec
P2 28.80 usec
PL1 0 dB
PL1W 23.41078186 W
SFO1 700.0028000 MHz

======== CHANNEL f2 ========
NUC2 13C
P3 9.20 usec
PL2 0 dB
PL2W 106.75517273 W
SFO2 176.0327405 MHz

====== GRADIENT CHANNEL =====
GPNAM1 SINE.100
GPNAM2 SINE.100
GPNAM3 SINE.100
GPZ1 50.00 %
GPZ2 30.00 %
GPZ3 40.10 %
P16 1000.00 usec

F1 – Acquisition parameters
TD 210
SFO1 176.0327 MHz
FIDRES 167.650223 Hz
SW 200.000 ppm
FnMODE QF

F2 – Processing parameters
SI 2048
SF 700.0000164 MHz
WDW SINE
SSB 3
LB 0 Hz
GB 0
PC 1.00

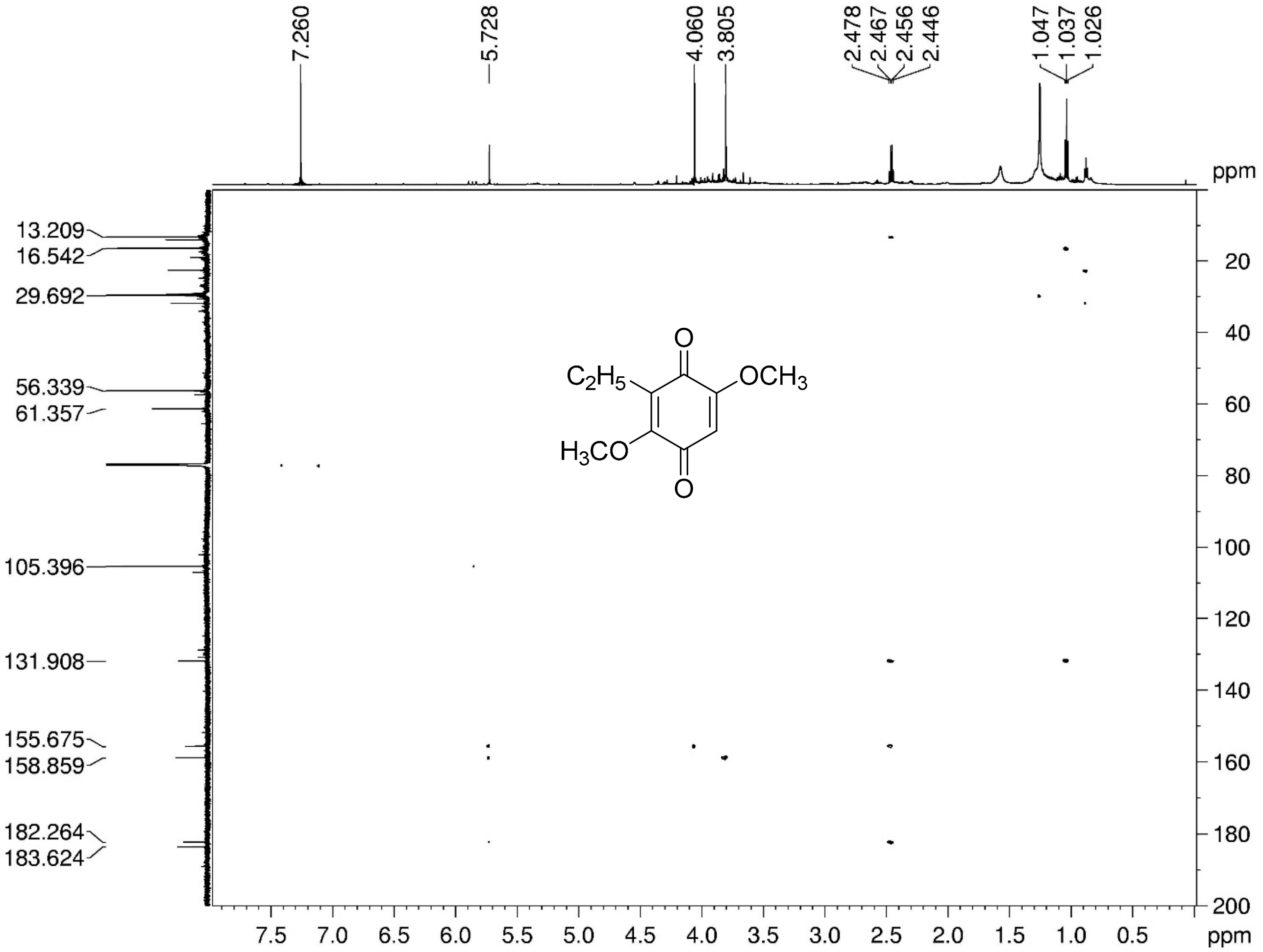

Figure S45. HMBC spectrum (700 MHz, $CDCl_3$) of dimethyl ether of **10.**

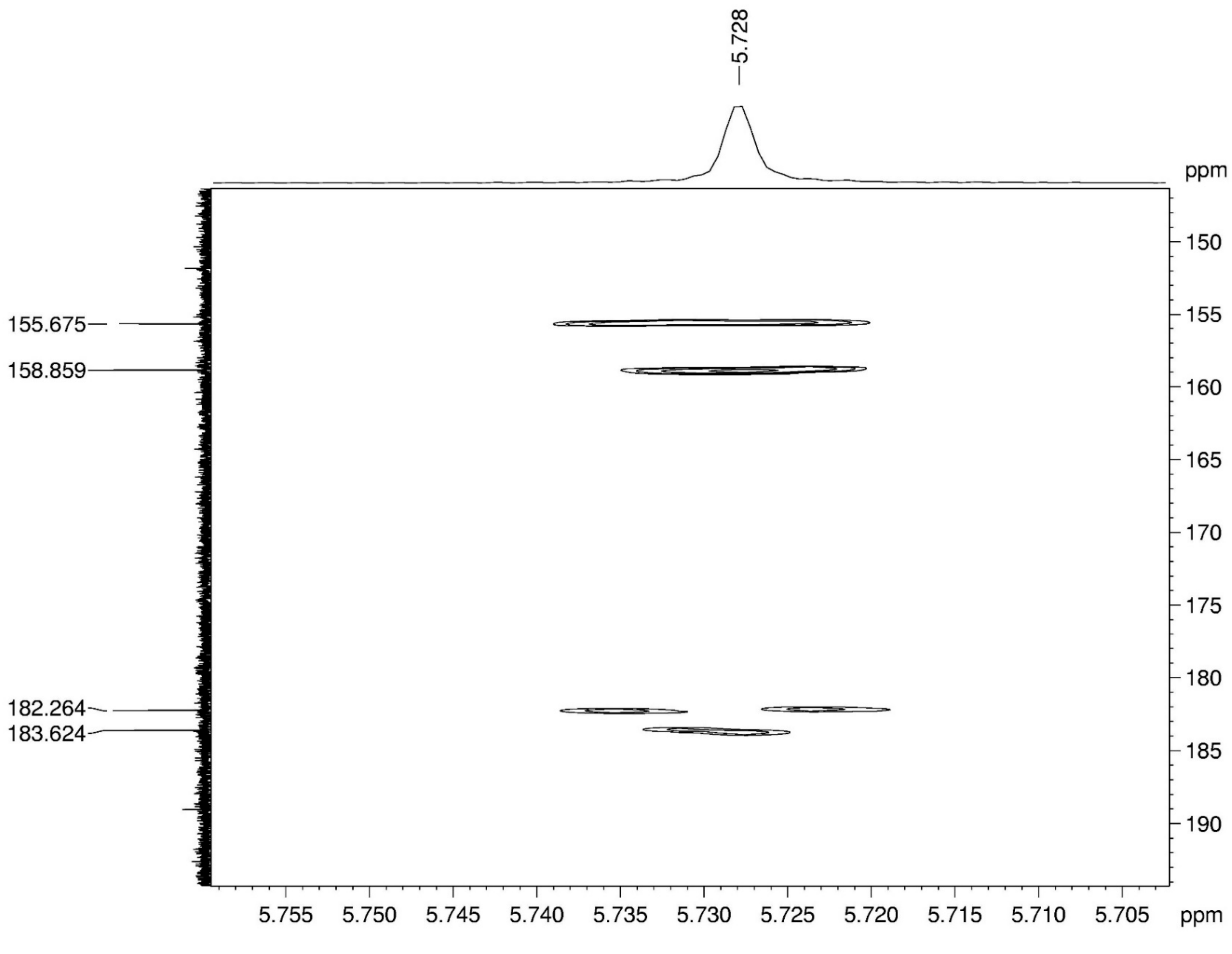

Figure S46. HMBC correlations of dimethyl ether of **10.**

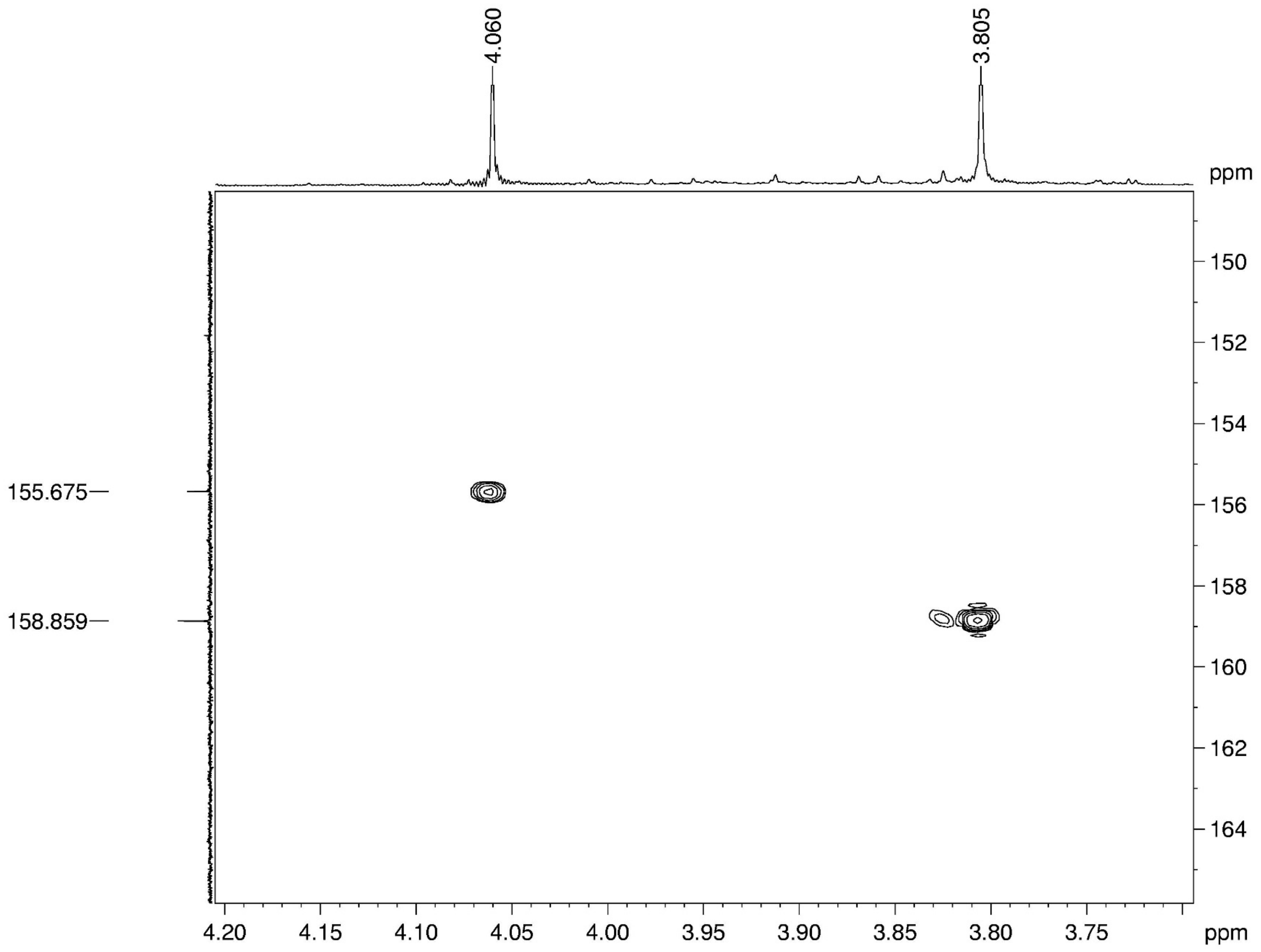

Figure S47. HMBC correlations of dimethyl ether of **10.**

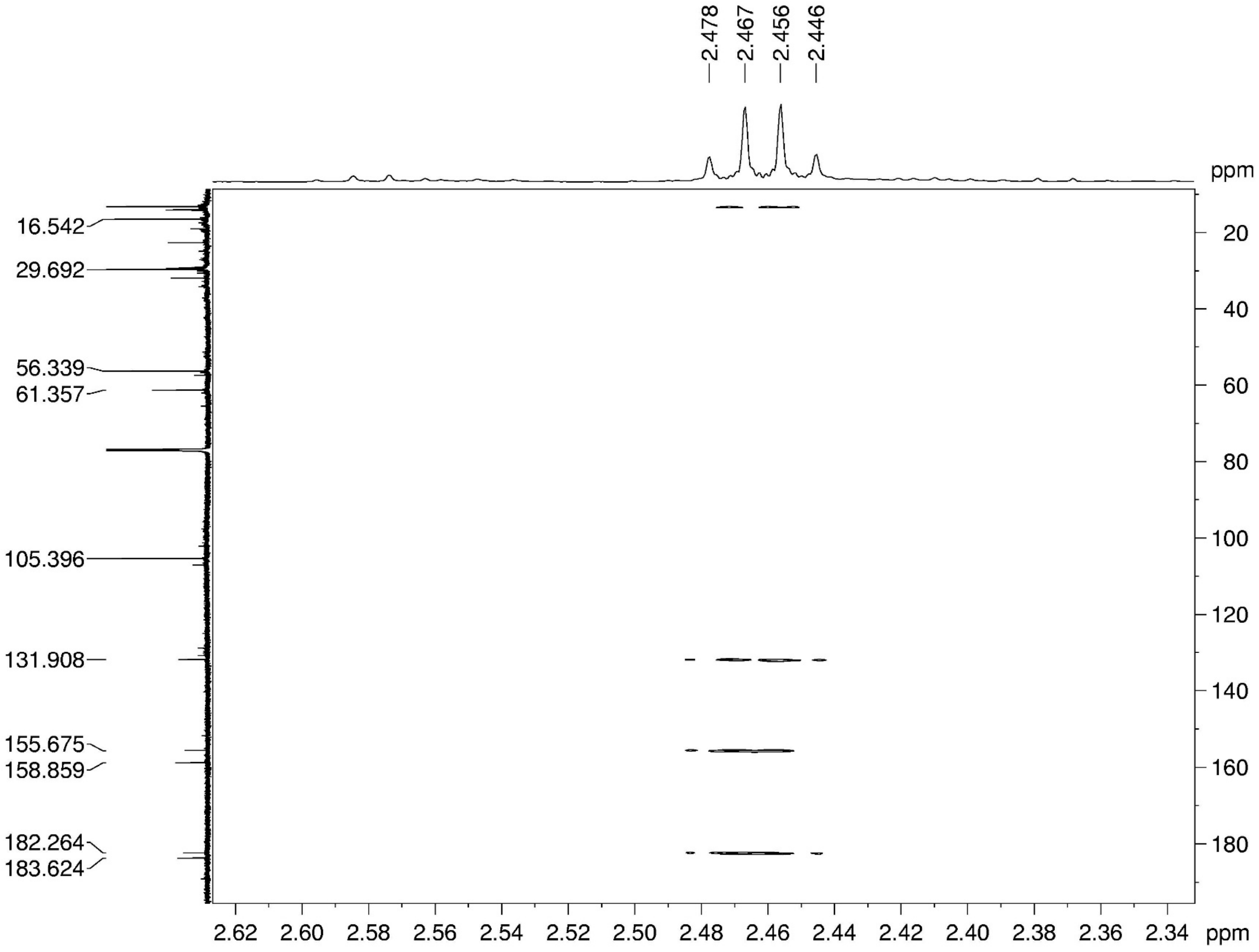

Figure S48. HMBC correlations of dimethyl ether of **10.**

Histochrome (Echinochrome A) Toxicity

The toxicity studies of the drug were carried out in the laboratory of drug toxicology of the National Medical Research Center for Cardiology of the Ministry of Health of the Russian Federation. The value of the median lethal dose (LD_{50}) of echinochrome A was determined as 153.7 mg/kg, whereas LD_{10} was equal to 122.7 mg/kg. A test dose was established for the Histochrome toxicity tests, which was 50 mg/kg or 1 mg per mouse.

Table S7. Histochrome toxicity values (intraperitoneal administration).

Animal	LD_{50} ± SD, mg/kg
Outbred mice, ♂	148 ± 22
White outbred mice	153,7
Wistar rats, ♂	87 ± 15
Wistar rats, ♀	69 ± 12
Chinchilla rabbits	125

Histochrome (Echinochrome A) Mutagenicity

Mutagenicity tests of Histochrome were carried out in the laboratory of drug toxicology of the National Medical Research Center for Cardiology of the Ministry of Health of the Russian Federation and in the Zakusov Institute of Pharmacology. The following criteria were considered:

1. Chromosomal aberrations in mammalian bone marrow cells;
2. Gene mutations in microorganisms in the Ames test;
3. Dominant lethal mutations in the germ cells of mice;
4. DNA damaging effects in the SOS-chromotest.

Table S8. Accounting for chromosomal aberrations in mammalian bone marrow cells.

Dose	Number of cells	on 100 cells					Total Damaged Metaphases (%)
		Gaps	Single fragments	Paired fragments	Changes	Cells with MD*	
male C57Bl/6							
Control	500	0.2	1.6	-	-	-	1.8 ± 0.6
Histochrome 1 mg/kg once	500	0.2	1.4	-	-	-	1.6 ± 0.5
Histochrome 10 mg/kg once	500	0.2	1.2	-	-	-	1.6 ± 0.5
Histochrome 1 mg/kg 5 days	500	0.4	1.2	-	-	-	1.4 ± 0.5
female C57B1/6							
Control	500	0.2	1.6	-	-	-	1.6 ± 0.5
Histochrome 1 mg/kg 5 days	500	0.4	1.6	-	-	-	1.8 ± 0.6

* MD, multiple damages (more than five chromosomal aberrations in the cell).

Table S9. The results of a study of the mutagenic effect of the Histochrome drug on indicator strains in the Ames test. Histochrome in concentrations of 0.1–1000 μg/plate does not exhibit a mutagenic effect in the Ames test.

Dose, mkg/plate	The geometric mean number of revertants per plate					
	TA98		TA1537		TA100	
	IMAM *	CMAM **	IMAM	CMAM	IMAM	CMAM
Histochrome						
0,1	37.9	39.6	19.8	19.7	127.6	134.2
1,0	38.8	40.0	19.9	19.9	130.8	147.3
10,0	40.3	37.9	20.8	23.2	132.2	135.4
100,0	38.6	41.4	21.9	23.0	99.3	135.7
1000,0	36.4	39.2	18.9	21.2	135.6	148.6
Control						
H_2O	38.8	41.9	20.5	25.2	138.3	144.6
2-aminoantracene, 10		1265.7		544.7		1664.2
2-nitroflurene, 0,2	376.1					
NaN, 1,5					1817.2	
9-aminoacridine, 10			863.3			

* IMAM, incomplete microsomal activating mixture; ** CMAM, complete microsomal activating mixture.

Table S10. The results of the study of the ability of the histochrome drug to induce dominant lethal mutations in the germ cells of mice. Histochrome does not induce dominant lethal mutations in mature sperm or late and early mouse spermatids.

Animal groups	Spermatogenesis stages	Number of analyzed females	Postimplantation mortality, %
Control	mature sperm	43	8.6
Histochrome 65 mg/kg		42	8.3
Control	late spermatids	42	8.9
Histochrome 65 mg/kg		42	8.2
Control	early spermatids	42	8.5
Histochrome 65 mg/kg		42	8.6

Histochrome in concentrations up to 10 mg/mL does not cause activation of the DNA repair system in *Escherichia coli* PQ 37, i.e., it does not have a DNA-damaging effect.
